# Supplementary material for: Modeling a hot, dry future: Substantial range reductions in suitable environment projected under climate change for a semiarid riparian predator guild
Source: PLoS One. 2024 May 6;19(5):e0302981. doi: 10.1371/journal.pone.0302981 (PMC11073737; doi:10.1371/journal.pone.0302981)
Supplement: S1 Fig — Ellipses represent the central tendencies (mean center ±1 standard deviation in each XY coordinate) of the data. Three time periods are categorized to reflect historical change. (PDF) [file pone.0302981.s001.pdf]

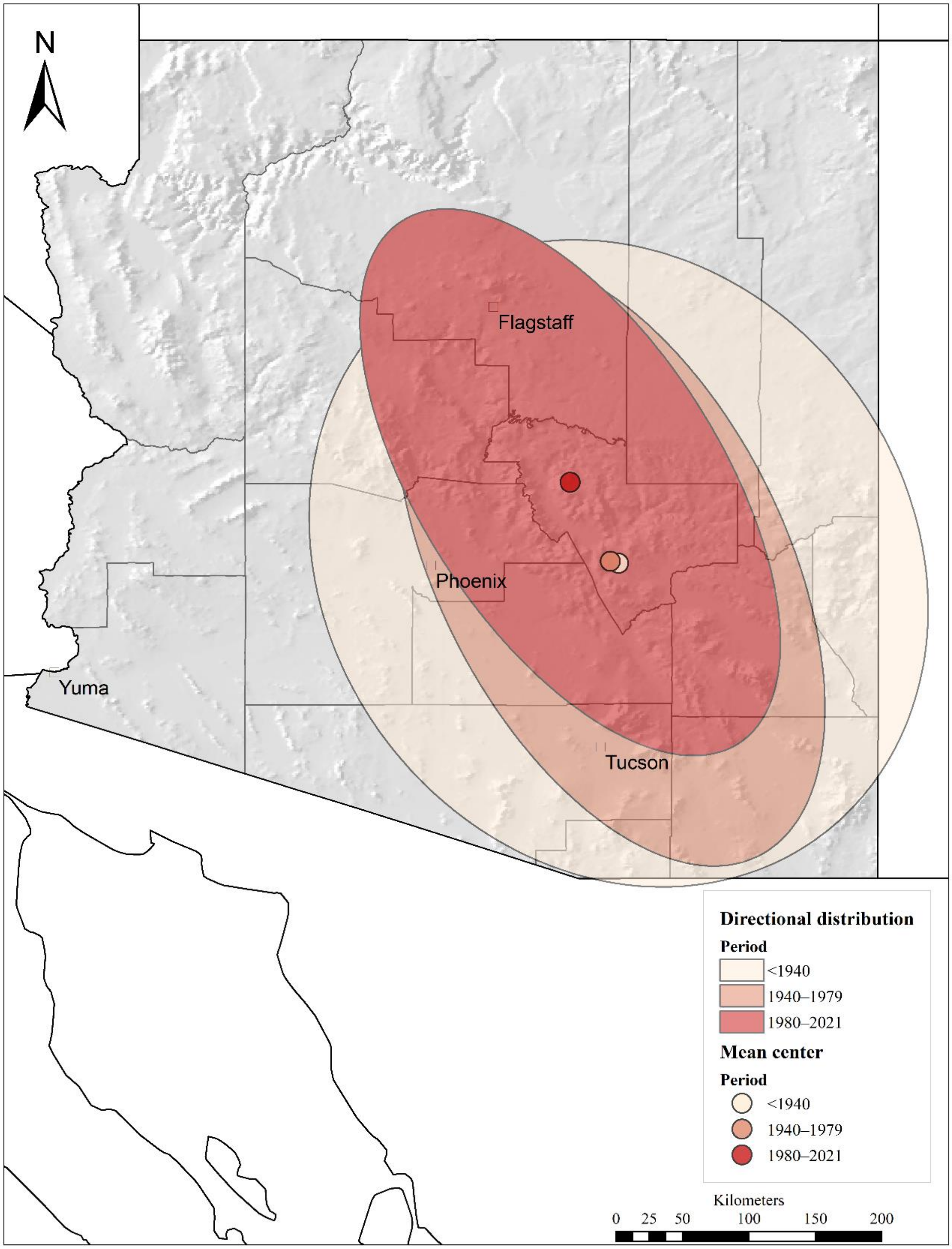

Fig S1. Directional distribution trends of *Thamnophis* gartersnake occurrences in Arizona. Ellipses represent the central tendencies (mean center ±1 standard deviation in each XY coordinate) of the data. Three time periods are categorized to reflect historical change.
