# Supplementary material for: Modeling a hot, dry future: Substantial range reductions in suitable environment projected under climate change for a semiarid riparian predator guild
Source: PLoS One. 2024 May 6;19(5):e0302981. doi: 10.1371/journal.pone.0302981 (PMC11073737; doi:10.1371/journal.pone.0302981)
Supplement: S2 Fig — Data are derived from weighted-mean algorithms of ensemble species distribution models. Bars represent means ± standard error. Pathways include the most optimistic (SSP126) and pessimistic (SSP585) emissions-limiting models. (PDF) [file pone.0302981.s002.pdf]

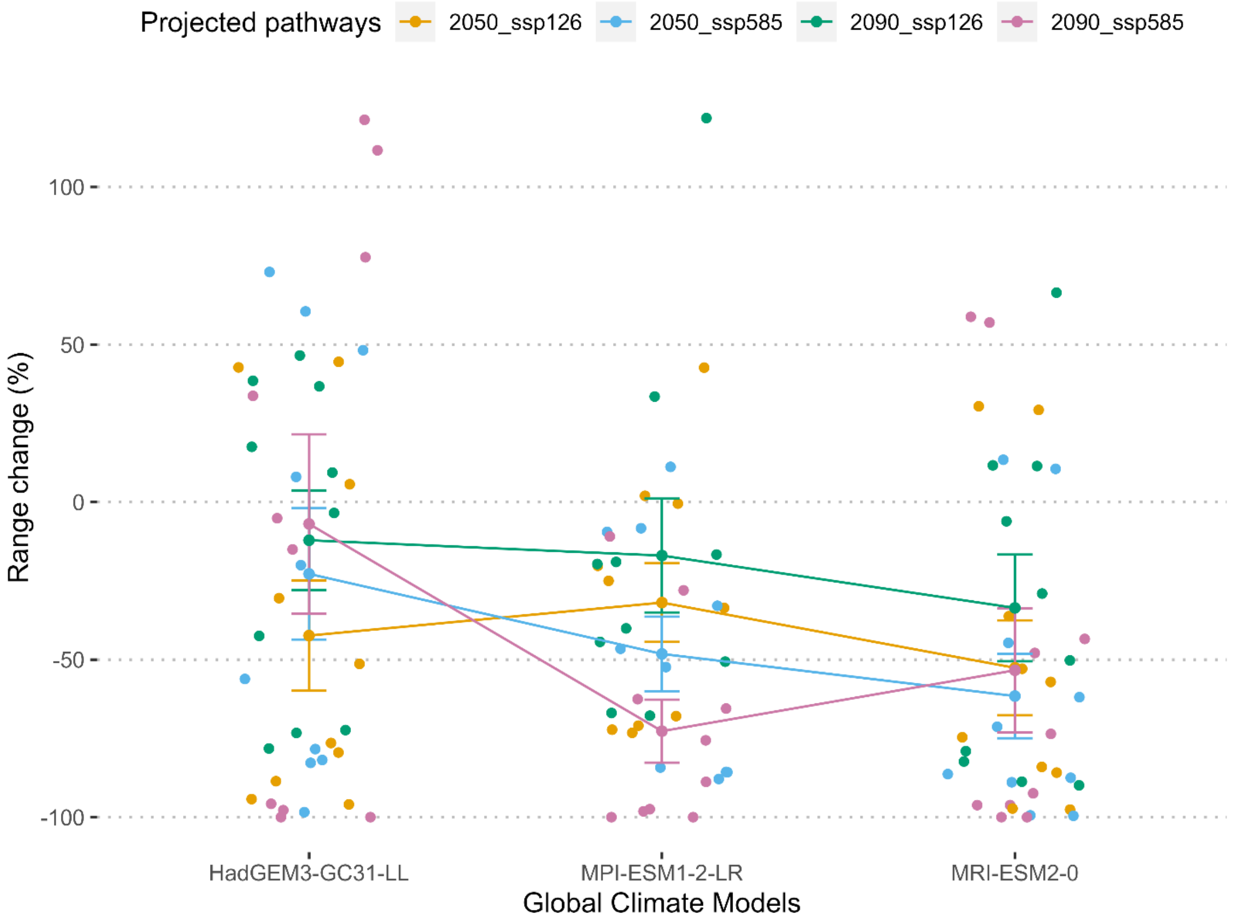

Fig S2. Estimated range changes across time for five *Thamnophis* gartersnake species in Arizona under three global climate models, two 20-year periods (median years 2050, 2090), and two shared socio-economic pathways (SSP). Data are derived from weighted-mean algorithms of ensemble species distribution models. Bars represent means ± standard error. Pathways include the most optimistic (SSP126) and pessimistic (SSP585) emissions-limiting models.
