# Supplementary material for: Modeling a hot, dry future: Substantial range reductions in suitable environment projected under climate change for a semiarid riparian predator guild
Source: PLoS One. 2024 May 6;19(5):e0302981. doi: 10.1371/journal.pone.0302981 (PMC11073737; doi:10.1371/journal.pone.0302981)
Supplement: S3 Fig — Projections based on weighted-means ensemble models generated from the MRI-ESM2-0 global climate model. Data columns represent present (1980–2021), near future (“2050”, i.e., 2041–2060 median), and distant future (“2090”, i.e., 2081–2100 median) projections of two future shared socio-economic pathways (SSP) that represent optimistic (SSP126) and pessimistic (SSP585, i.e., status quo) emissions-limiting models. (PDF) [file pone.0302981.s003.pdf]

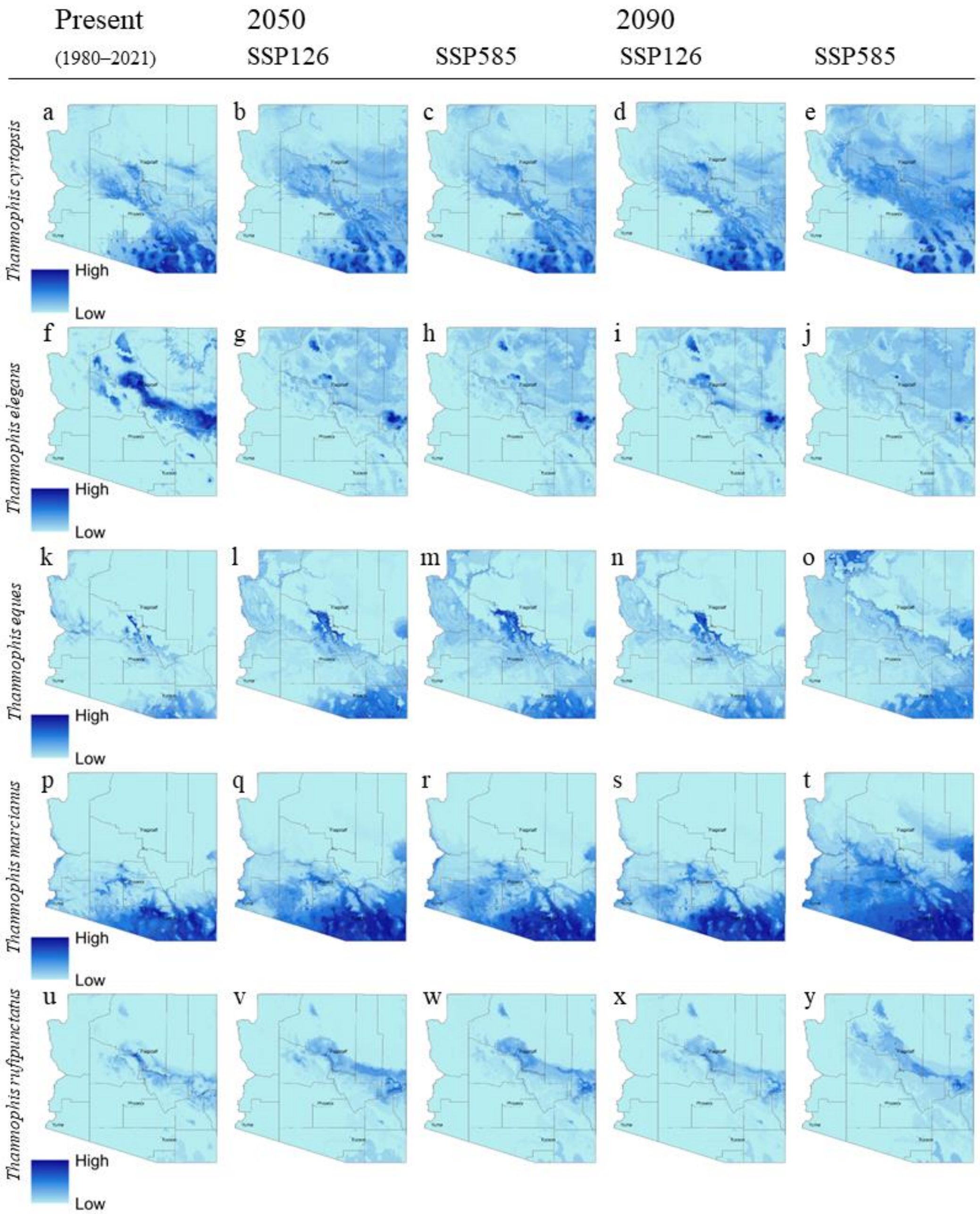

Fig S3a–y. Environmental suitability maps for *Thamnophis* gartersnakes in Arizona. Projections based on weighted-means ensemble models generated from the MRI-ESM2-0 global climate model. Data columns represent present (1980–2021), near future (“2050”, i.e., 2041–2060 median), and distant future (“2090”, i.e., 2081–2100 median) projections of two future shared socio-economic pathways (SSP) that represent optimistic (SSP126) and pessimistic (SSP585, i.e., status quo) emissions-limiting models. Individual panels are enlarged below.

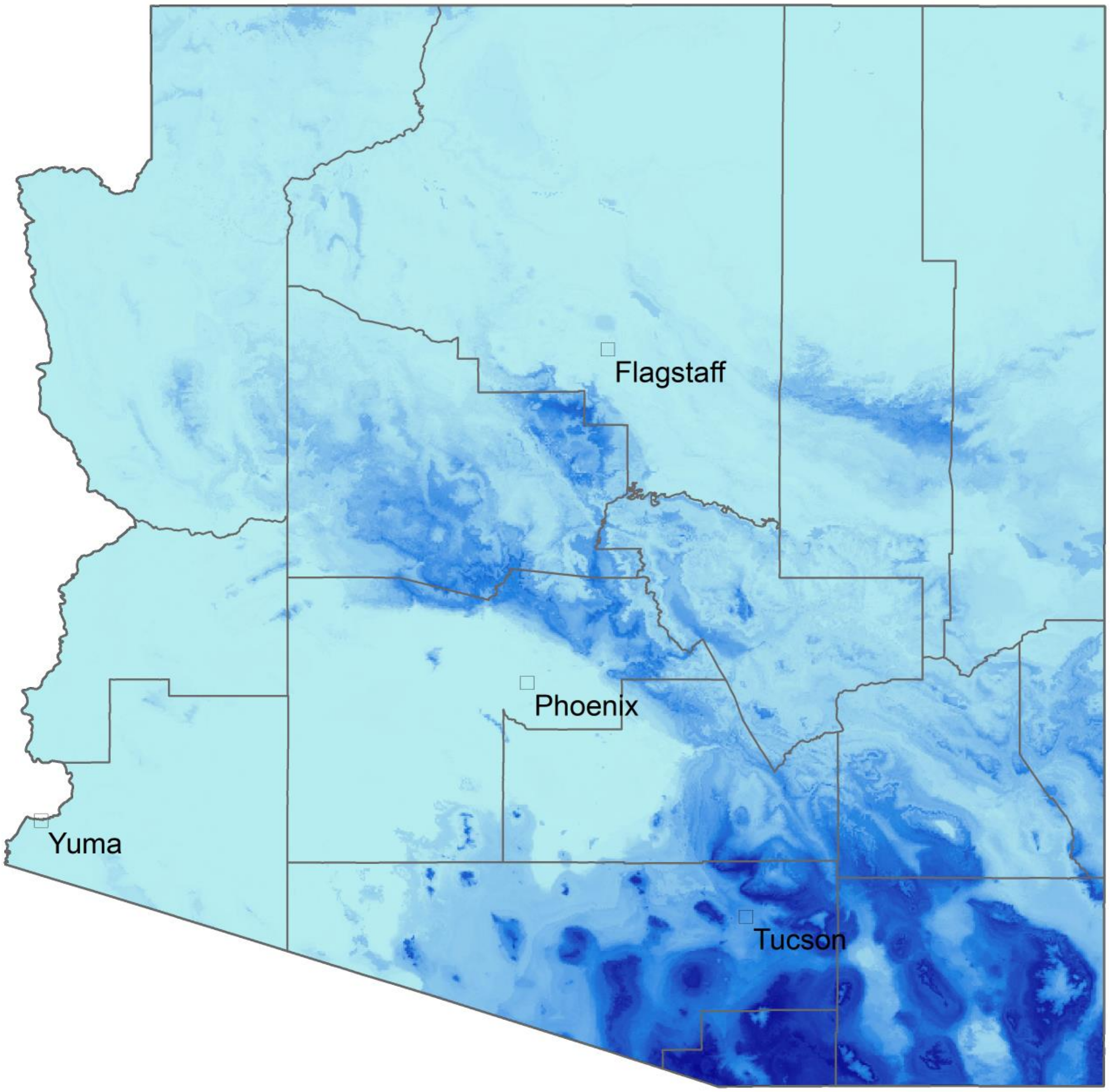

Fig S3a. Species: *Thamnophis cyrtopsis*; Time period: present (1980–2021); shared socio-economic pathway: n/a.

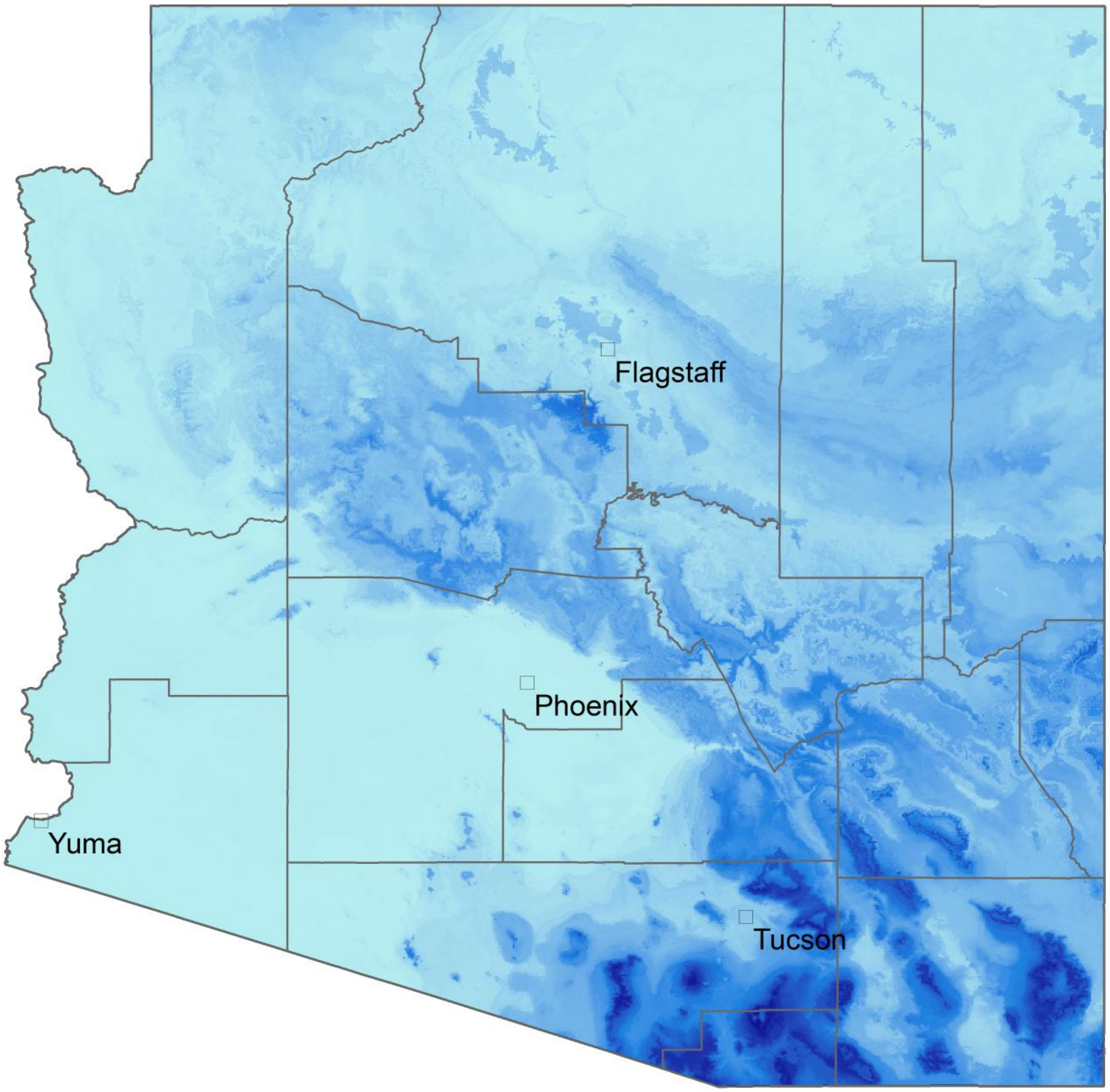

Fig S3b. Species: *Thamnophis cyrtopsis*; Time period: near future 2041–2060 (i.e., "2050" median); shared socio-economic pathway: "SSP126" (optimistic emissions-limiting models).

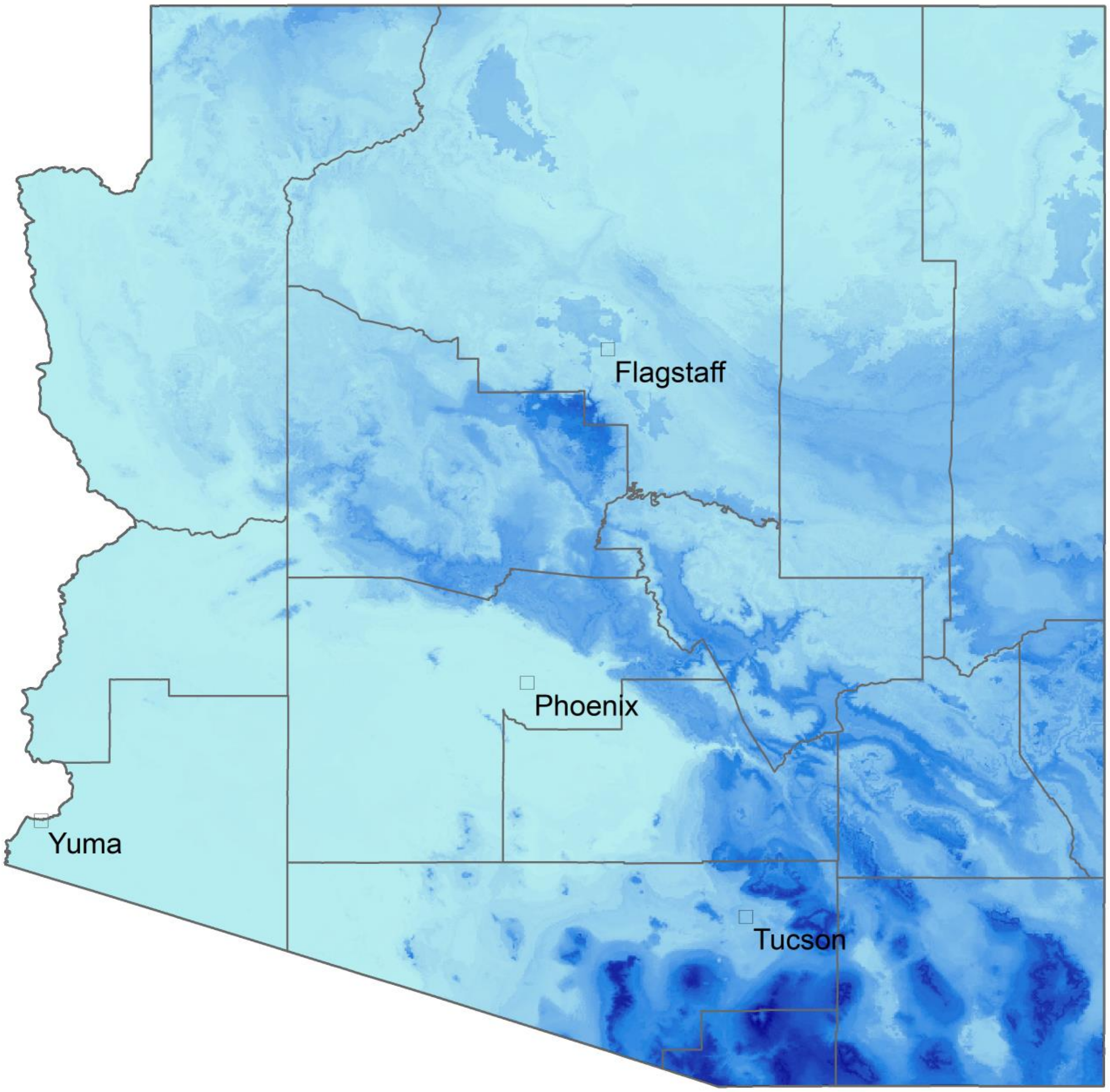

Fig S3c. Species: *Thamnophis cyrtopsis*; Time period: near future 2041–2060 (i.e., “2050” median); shared socio-economic pathway: “SSP585” (pessimistic ‘status quo’ emissions-limiting models).

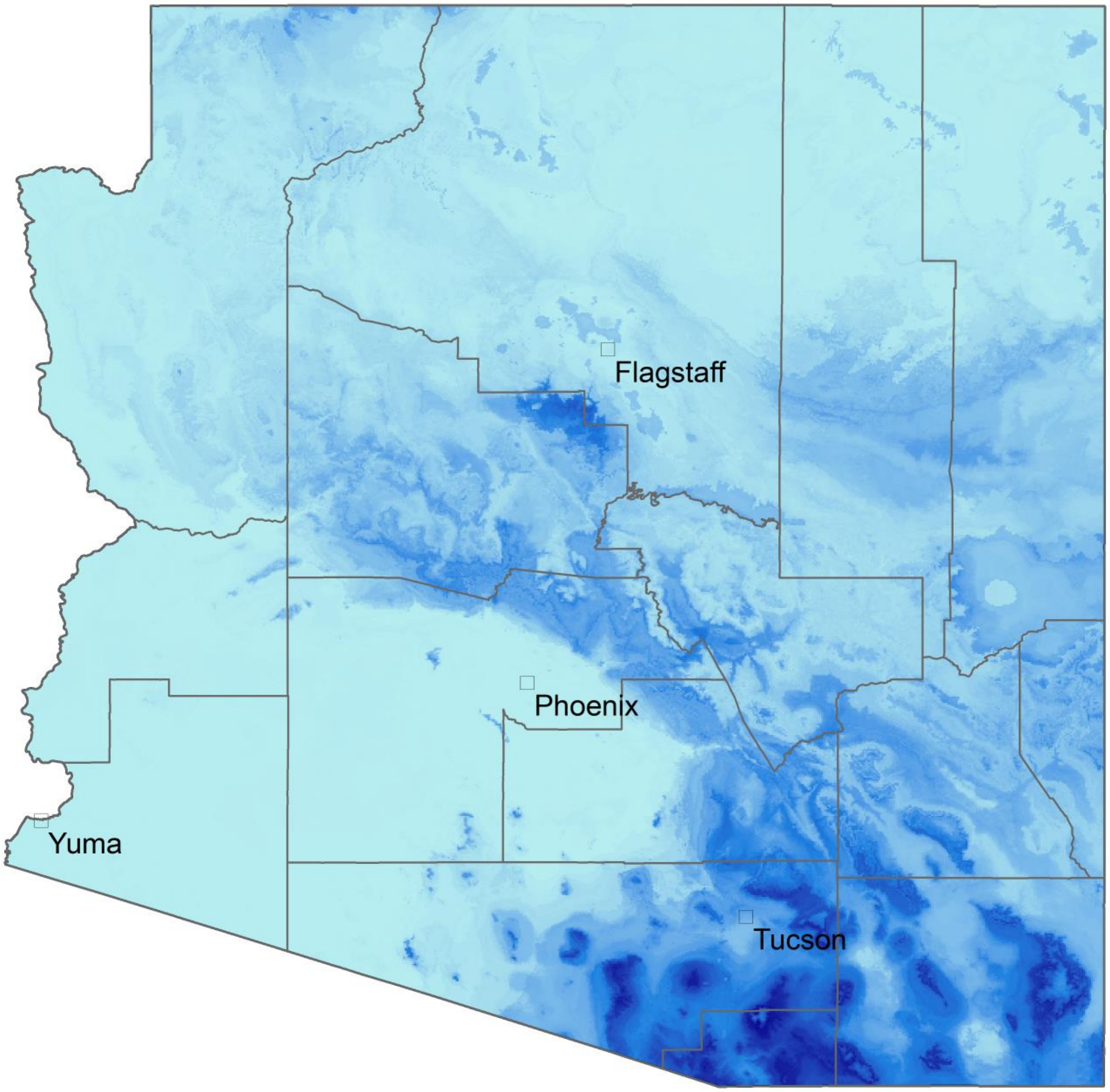

Fig S3d. Species: *Thamnophis cyrtopsis*; Time period: distant future 2081–2100 (i.e., “2090” median); shared socio-economic pathway: “SSP126” (optimistic emissions-limiting models).

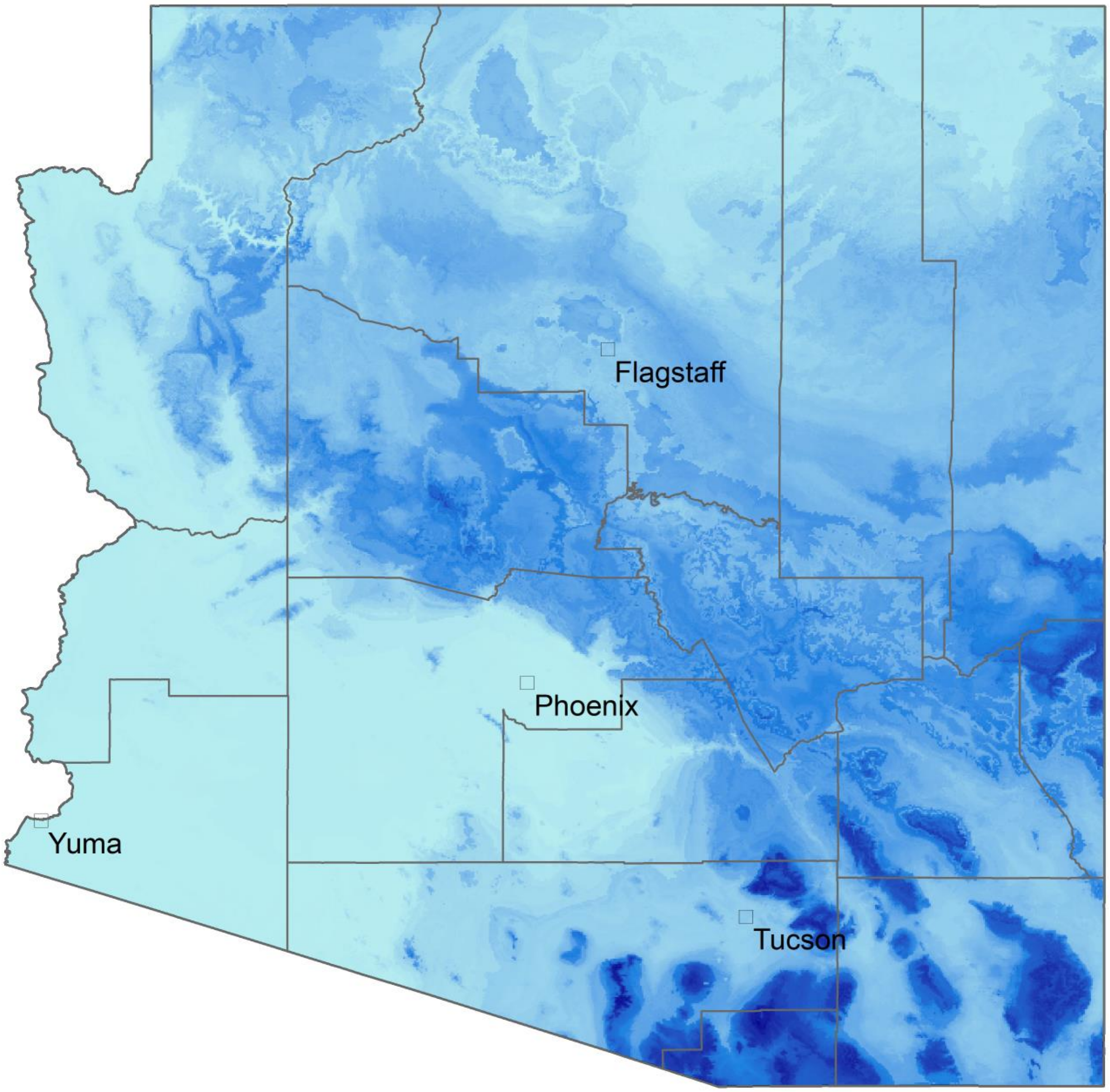

Fig S3e. Species: *Thamnophis cyrtopsis*; Time period: distant future 2081–2100 (i.e., “2090” median); shared socio-economic pathway: “SSP585” (pessimistic ‘status quo’ emissions-limiting models).

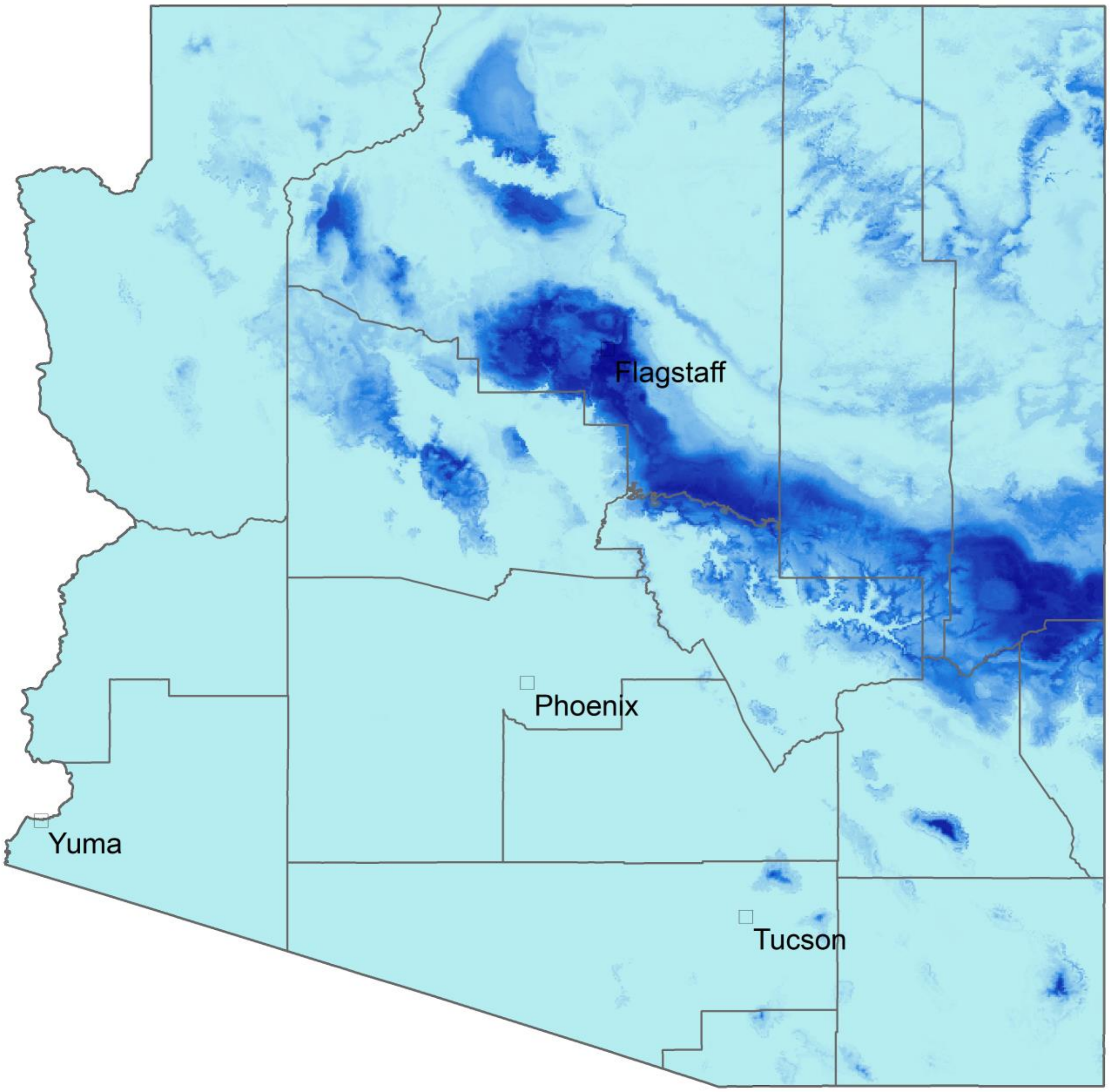

Fig S3f. Species: *Thamnophis elegans*; Time period: present (1980–2021); shared socio-economic pathway: n/a.

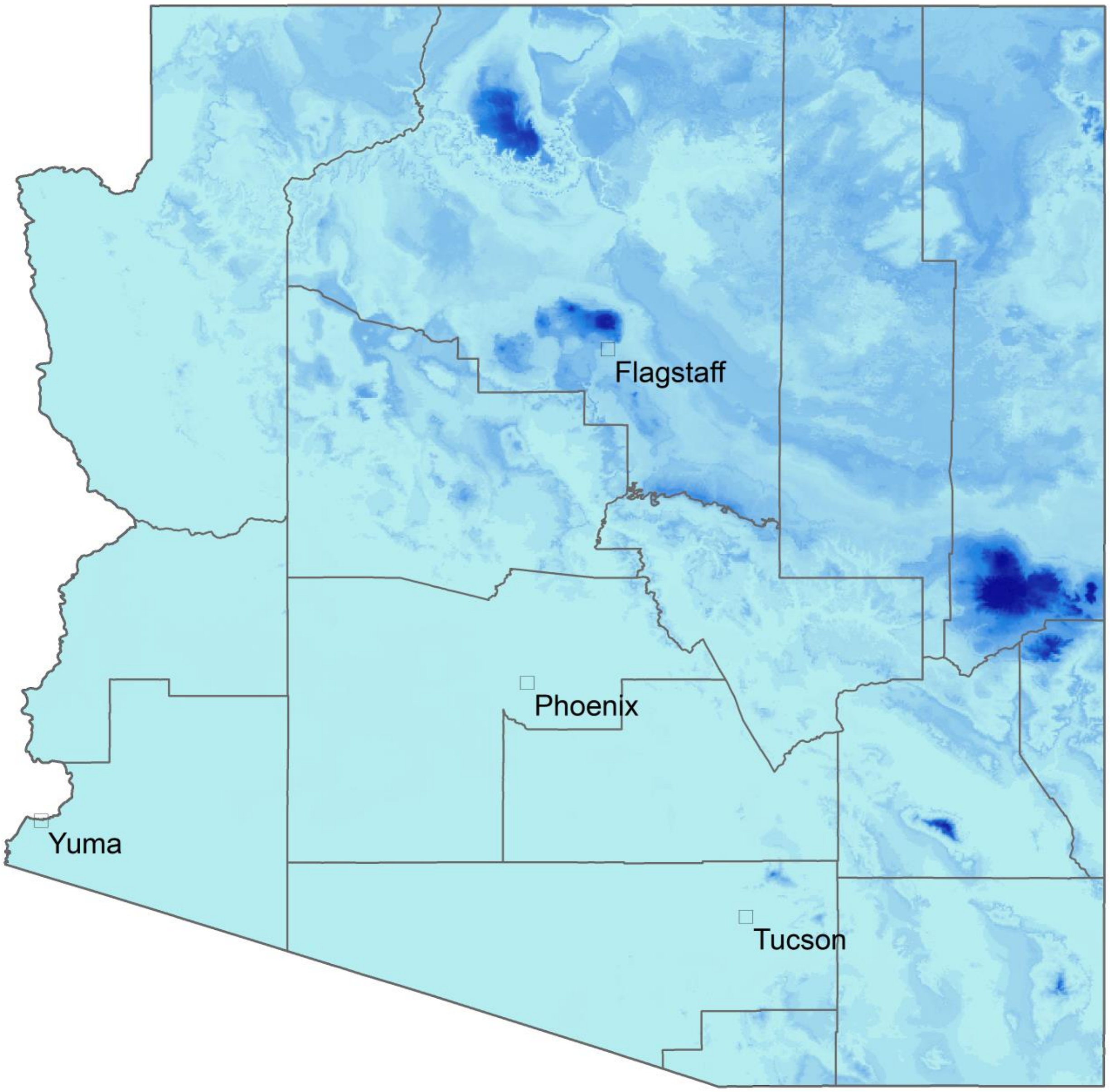

Fig S3g. Species: *Thamnophis elegans*; Time period: near future 2041–2060 (i.e., “2050” median); shared socio-economic pathway: “SSP126” (optimistic emissions-limiting models).

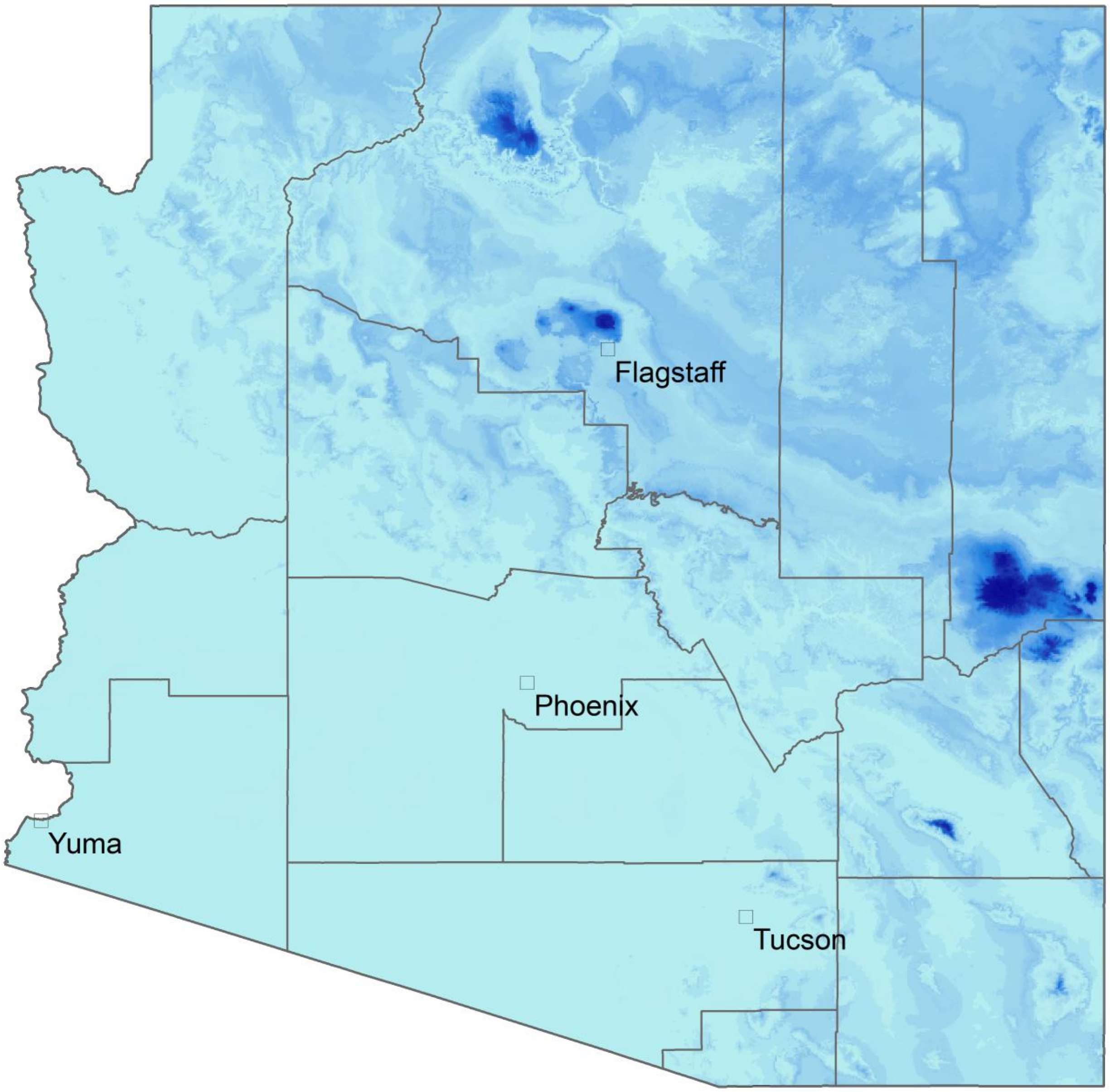

Fig S3h. Species: *Thamnophis elegans*; Time period: near future 2041–2060 (i.e., “2050” median); shared socio-economic pathway: “SSP585” (pessimistic ‘status quo’ emissions-limiting models).

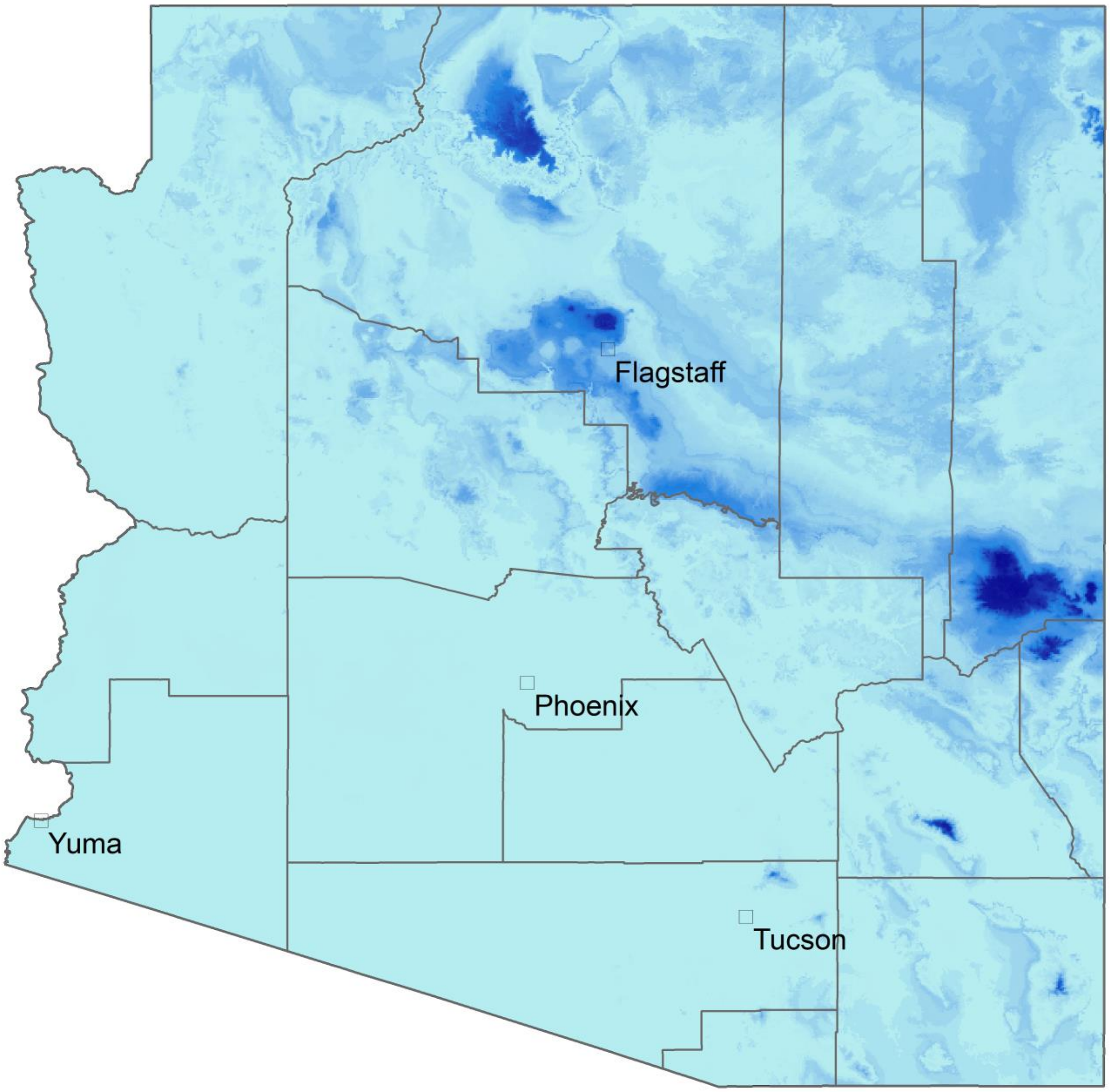

Fig S3i. Species: *Thamnophis elegans*; Time period: distant future 2081–2100 (i.e., “2090” median); shared socio-economic pathway: “SSP126” (optimistic emissions-limiting models).

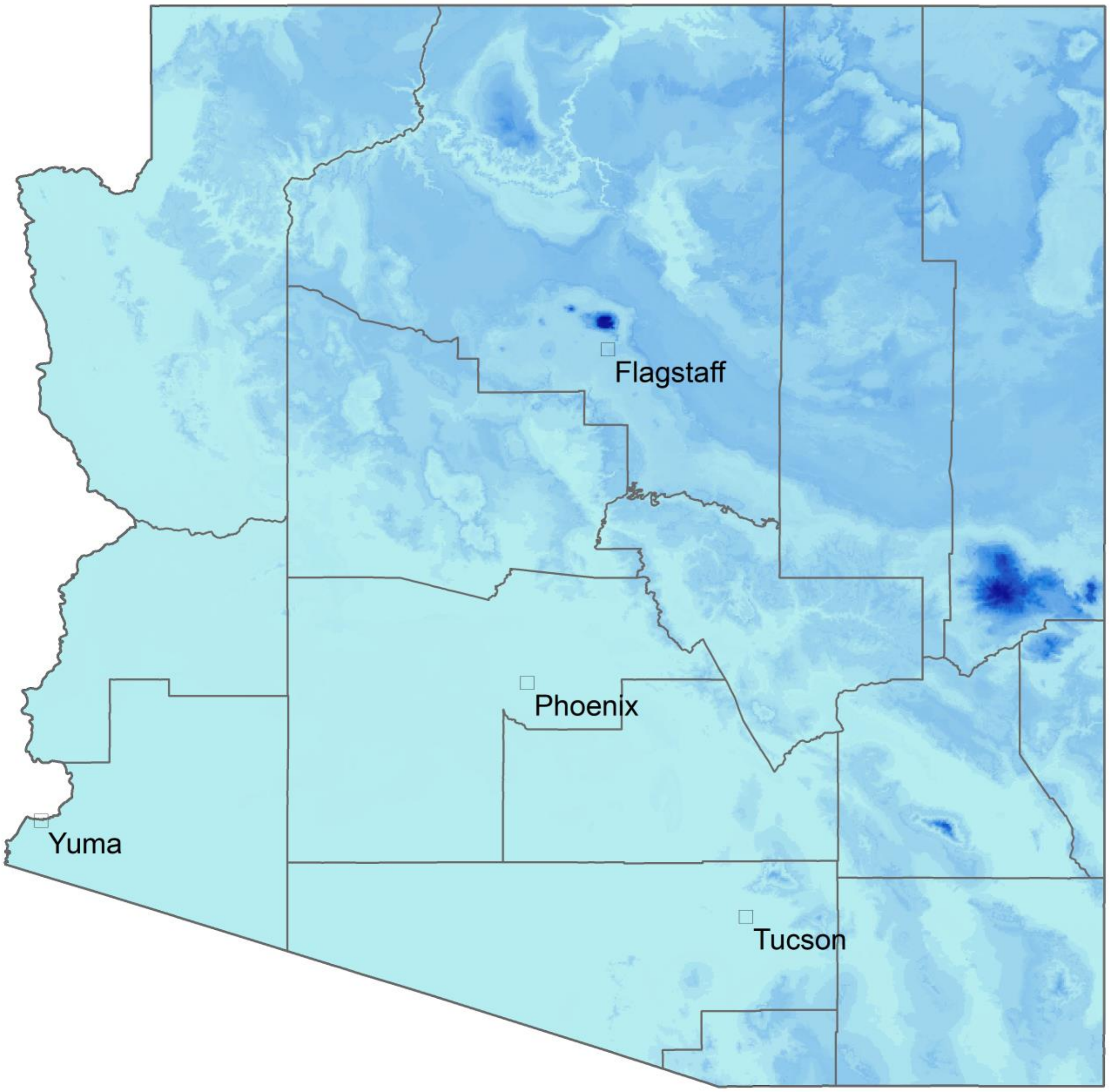

Fig S3j. Species: *Thamnophis elegans*; Time period: distant future 2081–2100 (i.e., “2090” median); shared socio-economic pathway: “SSP585” (pessimistic ‘status quo’ emissions-limiting models).

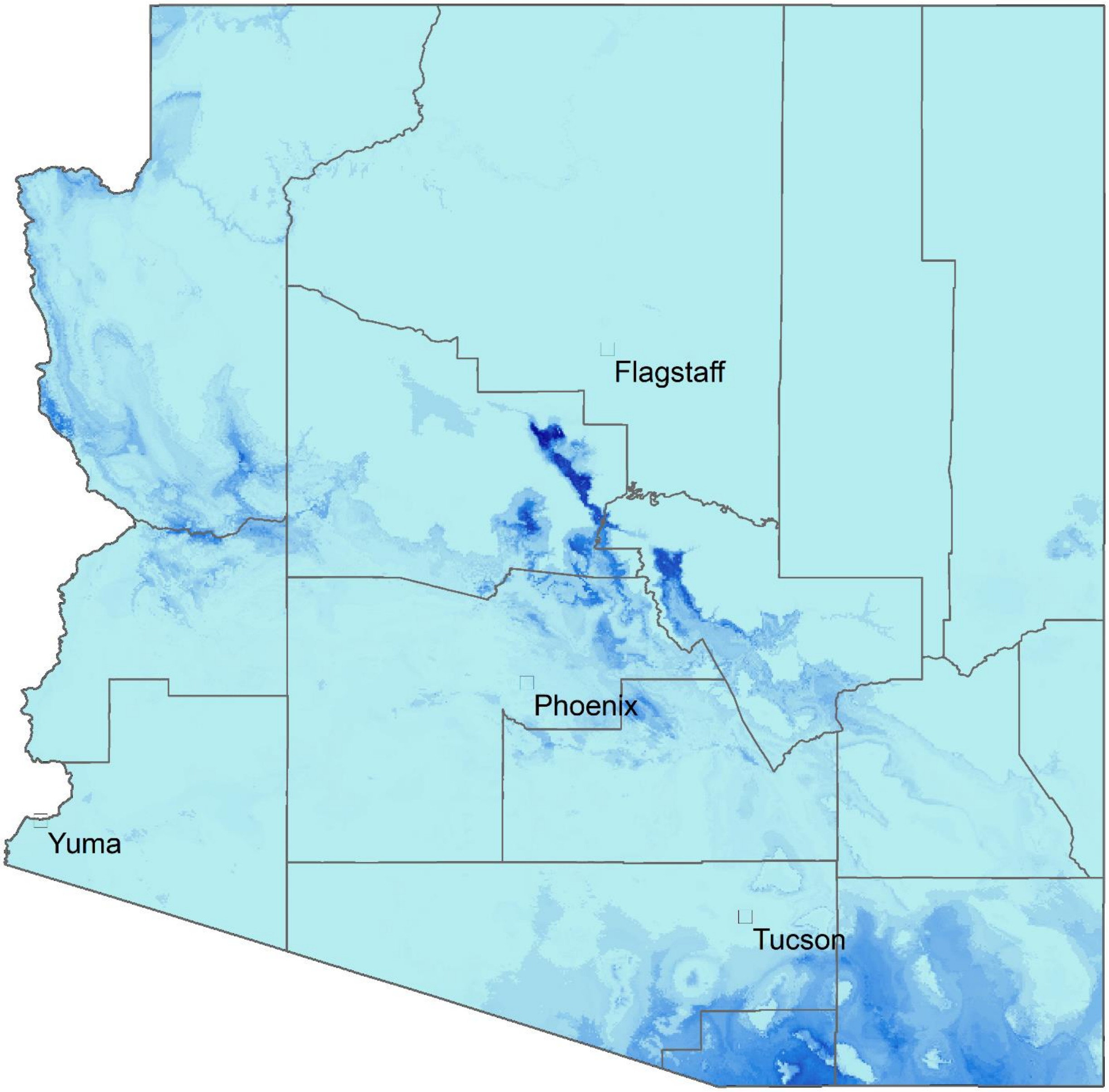

Fig S3k. Species: *Thamnophis eques*; Time period: present (1980–2021); shared socio-economic pathway: n/a.

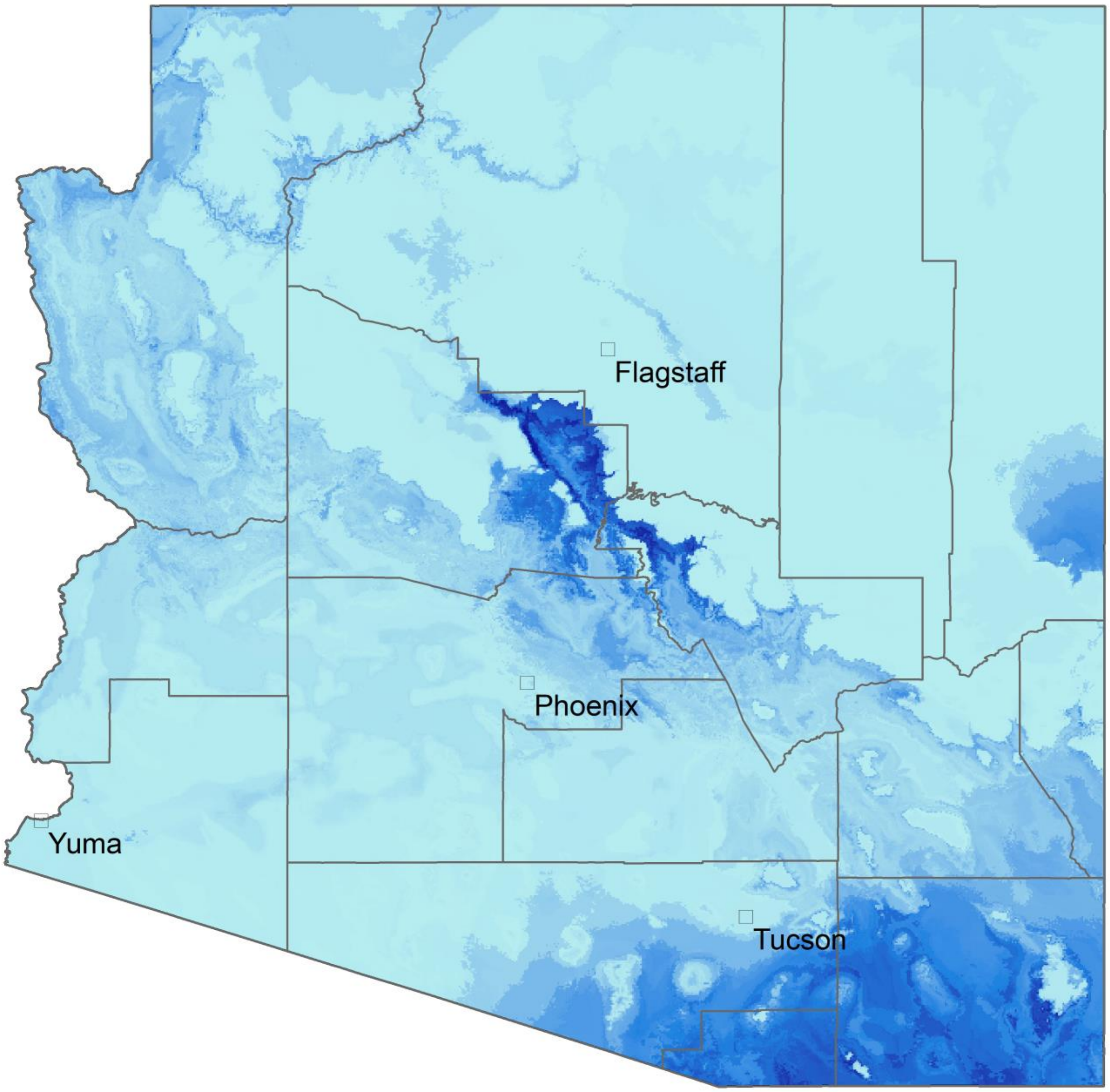

Fig S3l. Species: *Thamnophis eques*; Time period: near future 2041–2060 (i.e., “2050” median); shared socio-economic pathway: “SSP126” (optimistic emissions-limiting models).

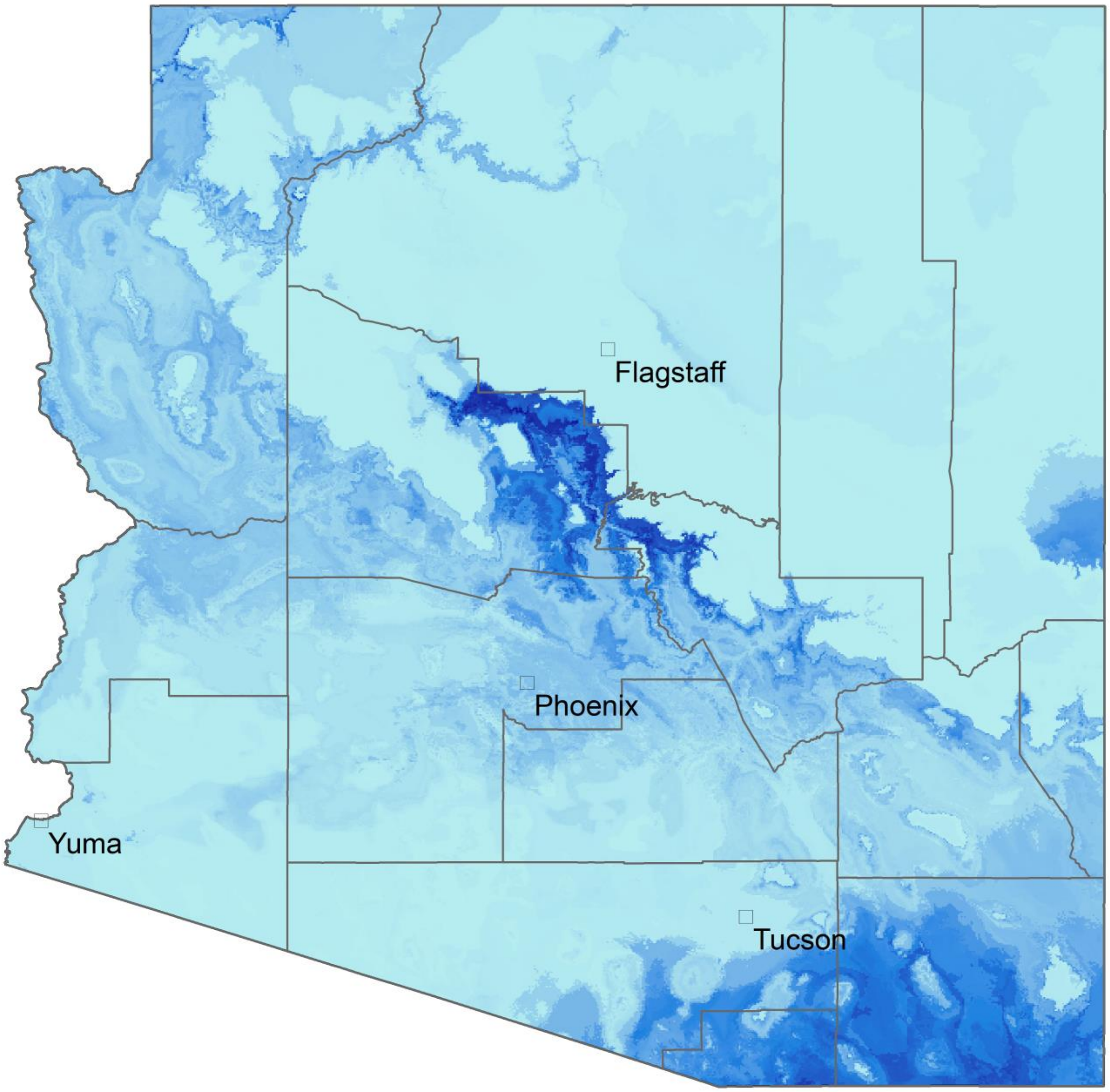

Fig S3m. Species: *Thamnophis eques*; Time period: near future 2041–2060 (i.e., “2050” median); shared socio-economic pathway: “SSP585” (pessimistic ‘status quo’ emissions-limiting models).

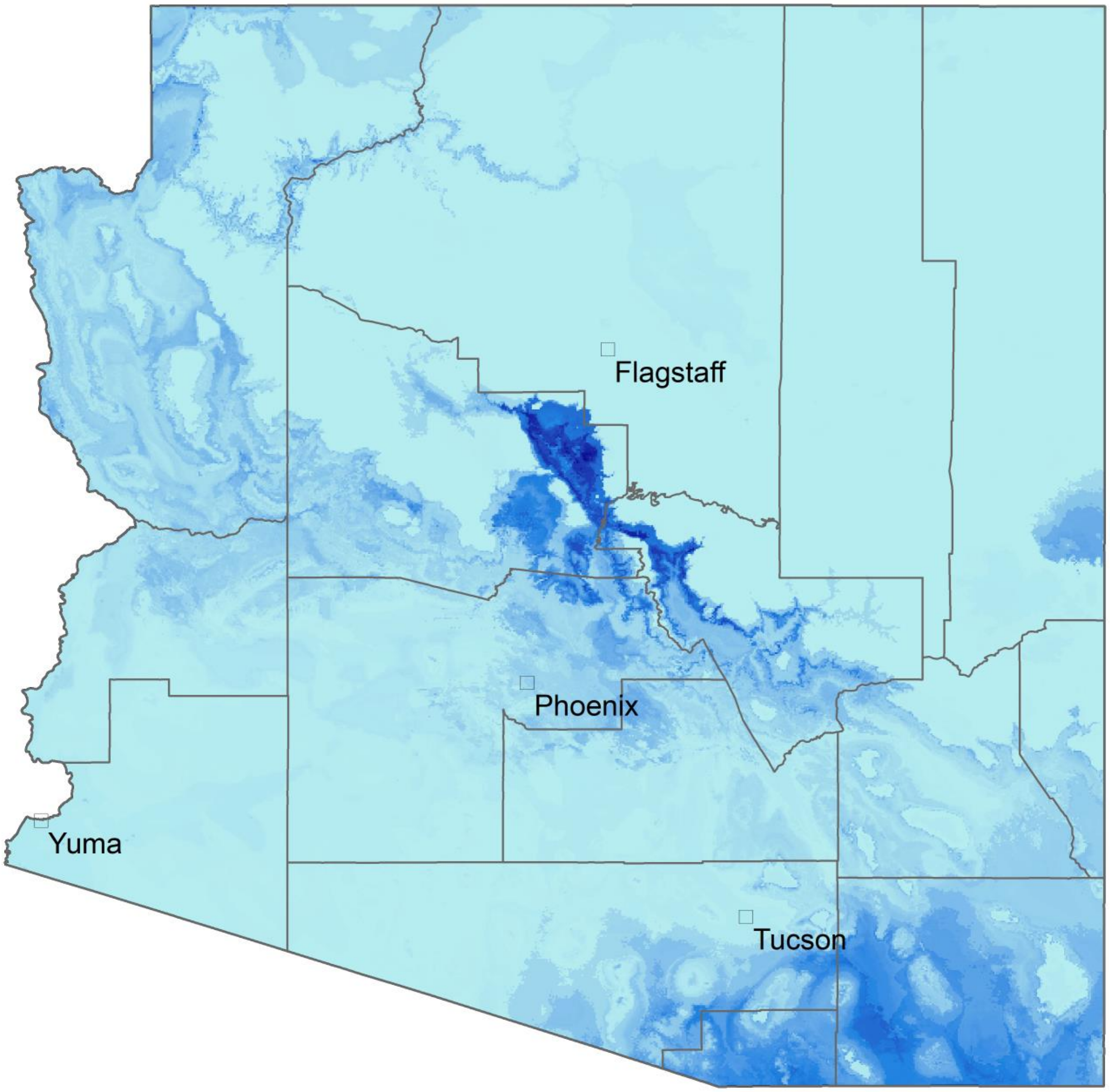

Fig S3n. Species: *Thamnophis eques*; Time period: distant future 2081–2100 (i.e., “2090” median); shared socio-economic pathway: “SSP126” (optimistic emissions-limiting models).

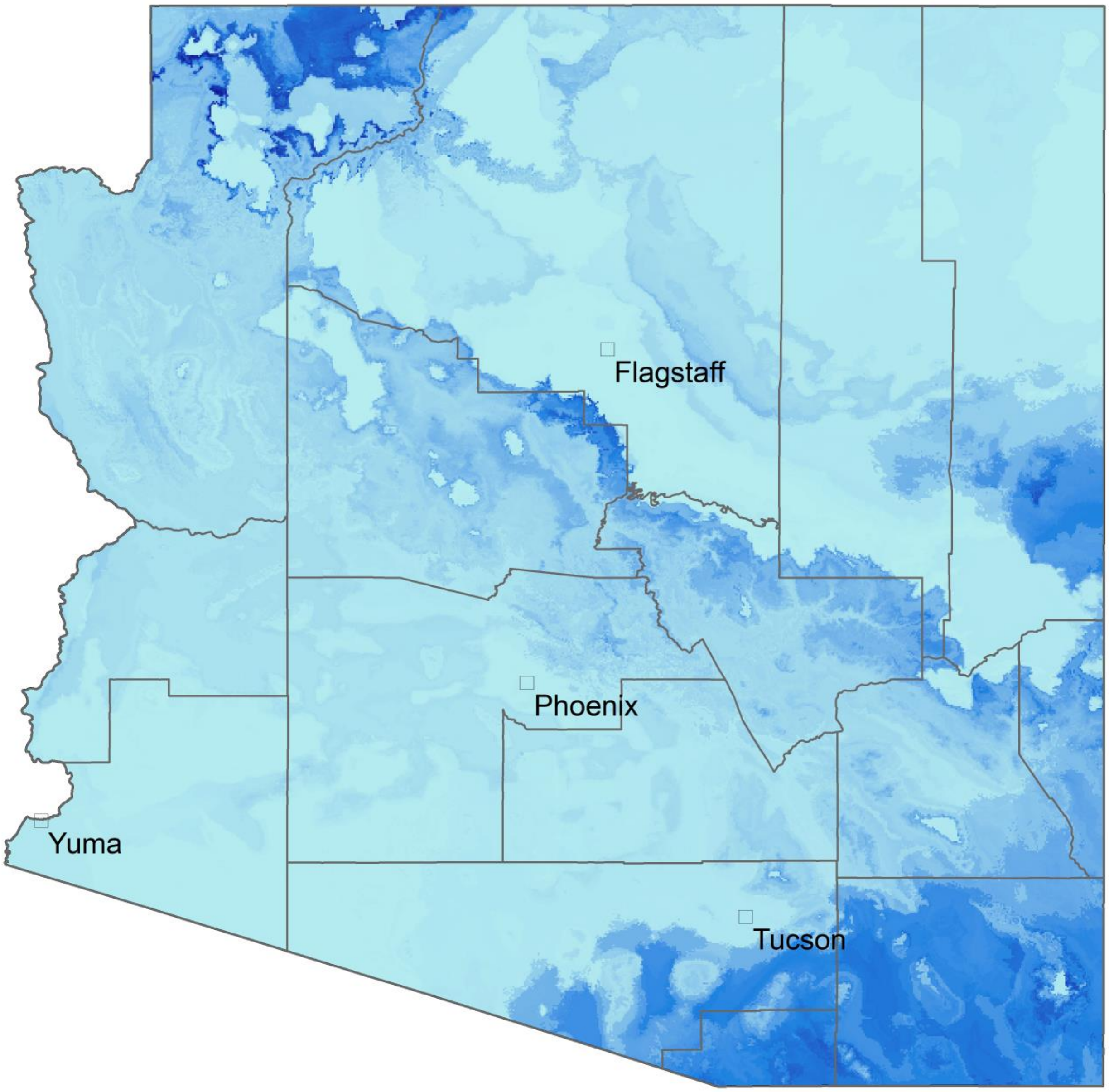

Fig S3o. Species: *Thamnophis eques*; Time period: distant future 2081–2100 (i.e., “2090” median); shared socio-economic pathway: “SSP585” (pessimistic ‘status quo’ emissions-limiting models).

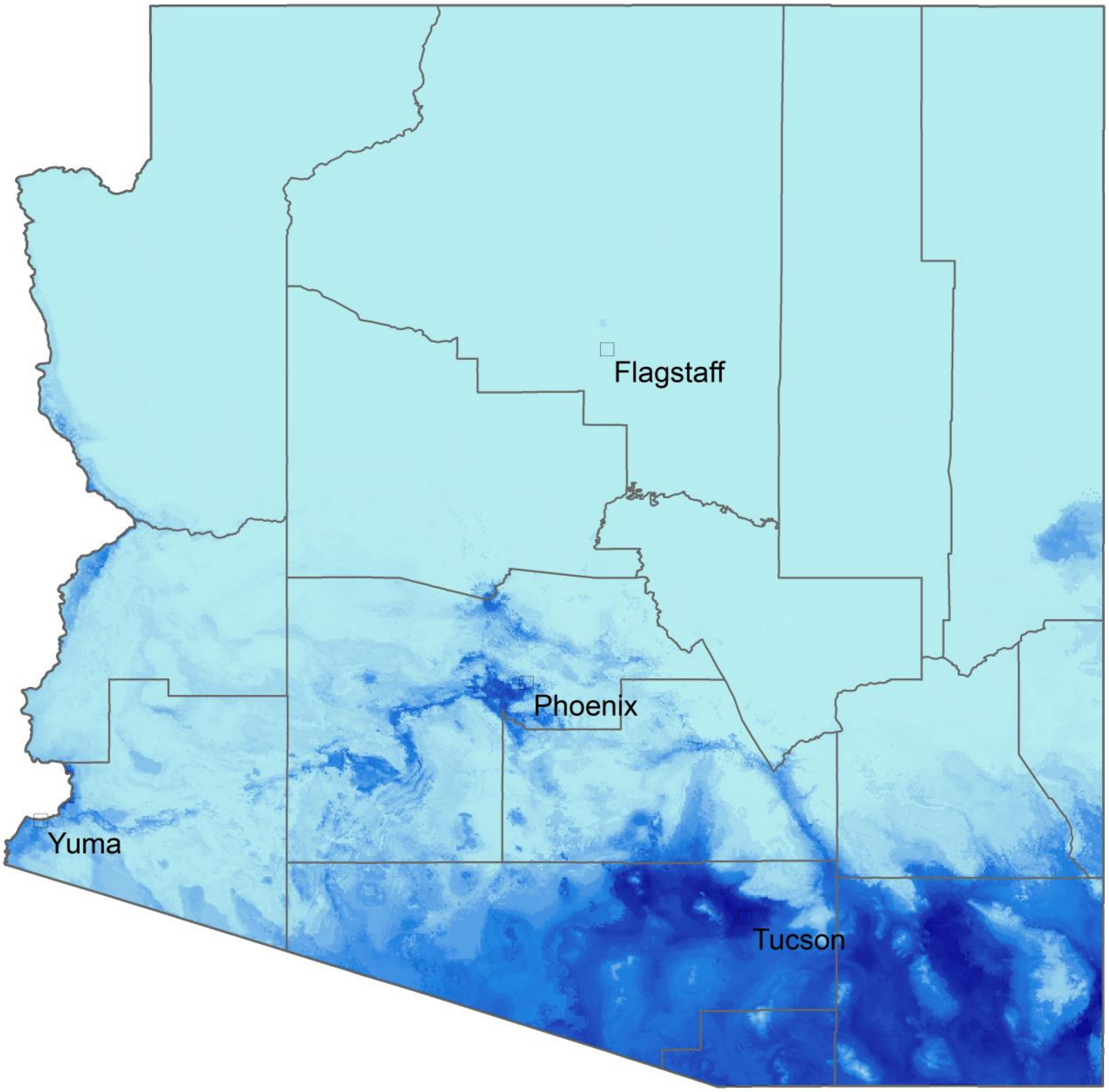

Fig S3p. Species: *Thamnophis marcianus*; Time period: present (1980–2021); shared socio-economic pathway: n/a.

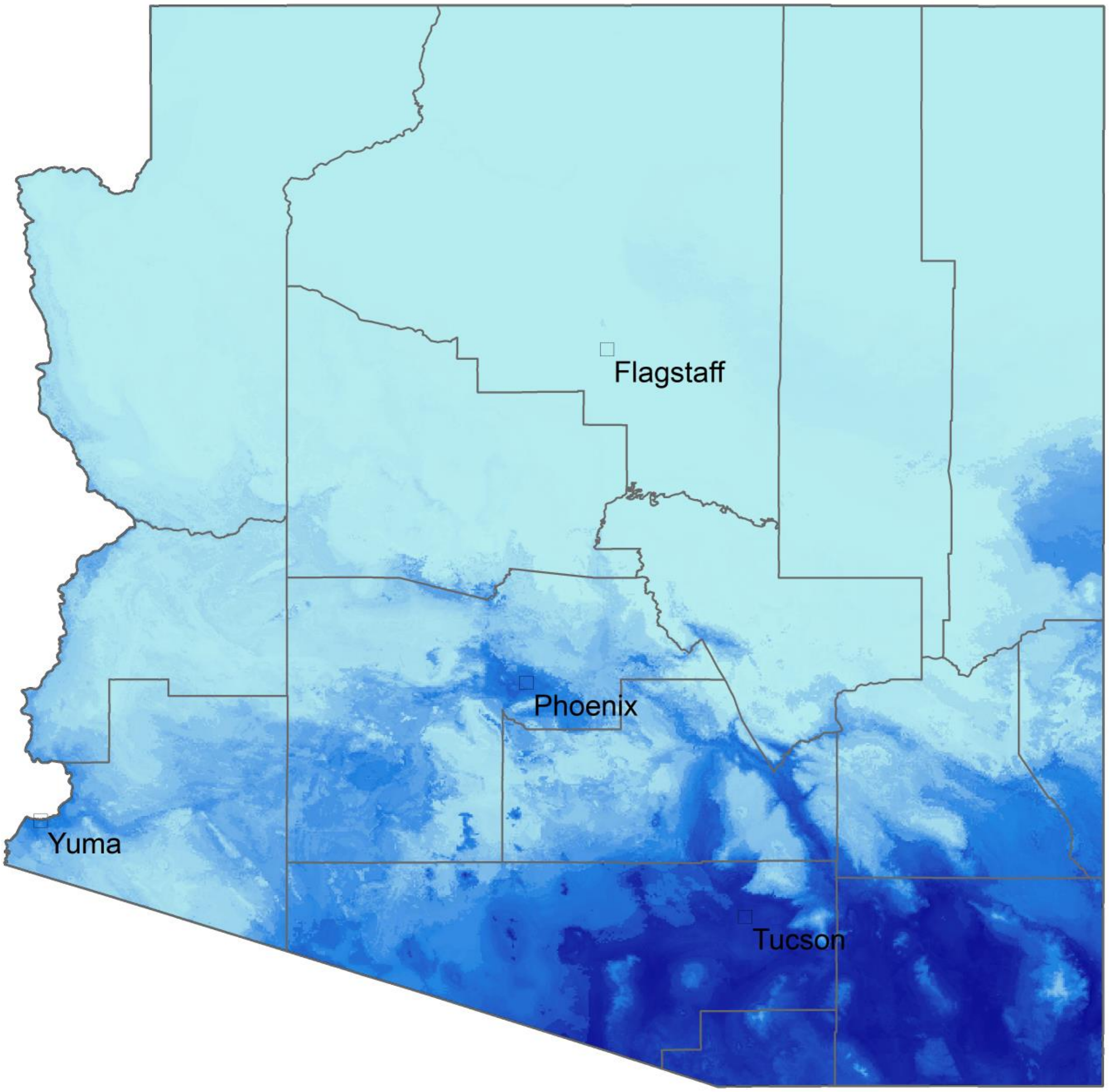

Fig S3q. Species: *Thamnophis marcianus*; Time period: near future 2041–2060 (i.e., “2050” median); shared socio-economic pathway: “SSP126” (optimistic emissions-limiting models).

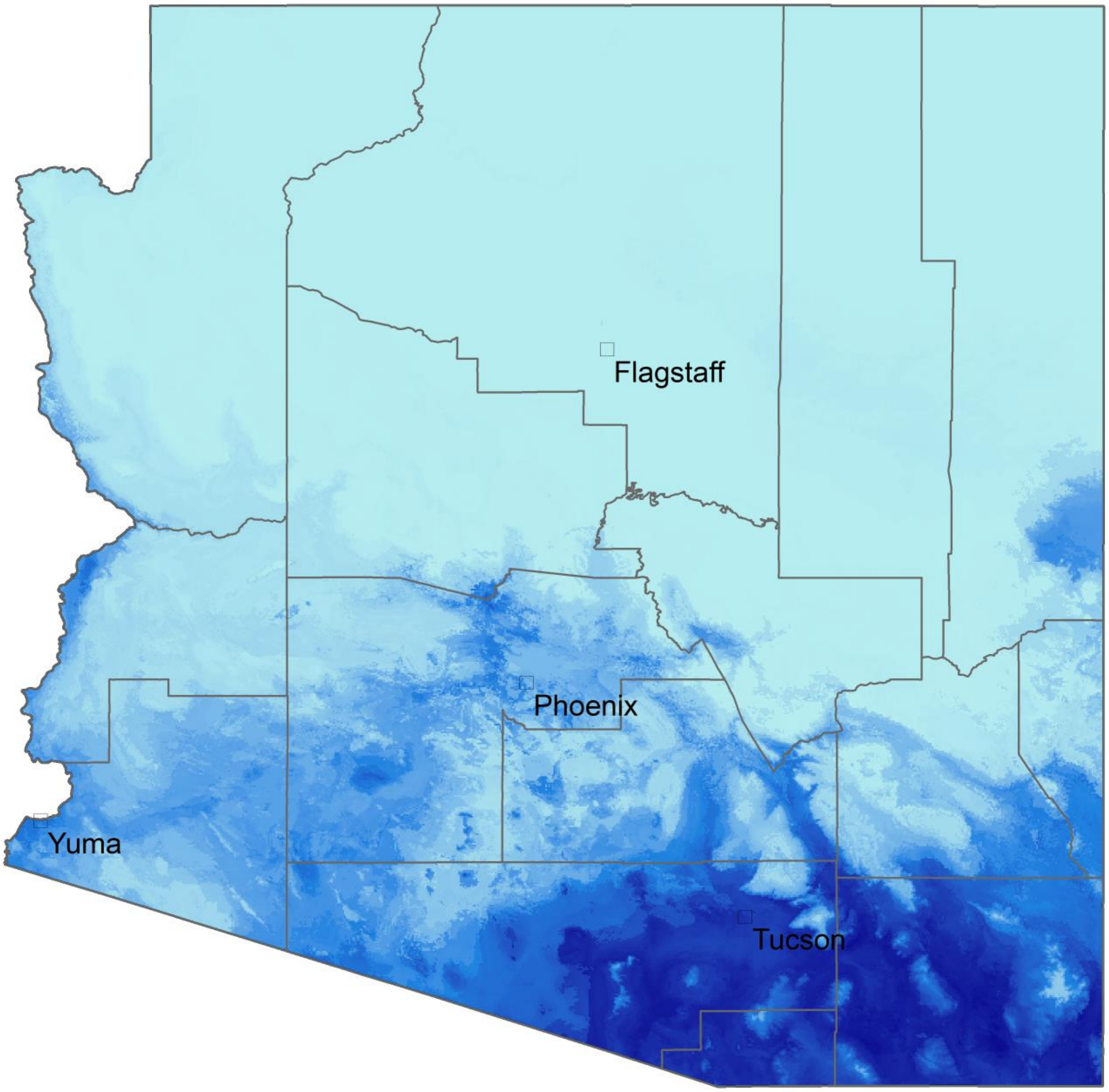

Fig S3r. Species: *Thamnophis marcianus*; Time period: near future 2041–2060 (i.e., “2050” median); shared socio-economic pathway: “SSP585” (pessimistic ‘status quo’ emissions-limiting models).

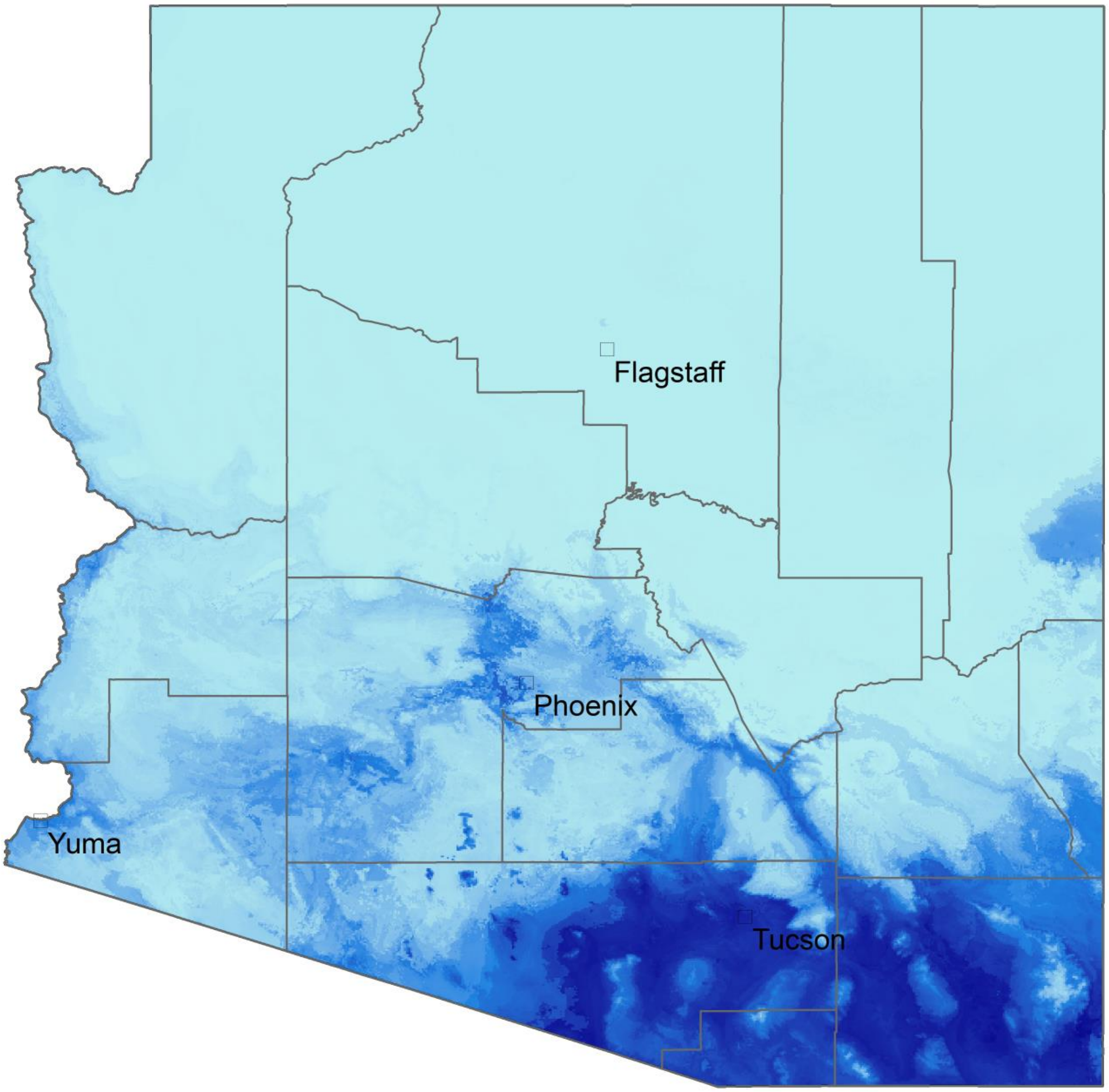

Fig S3s. Species: *Thamnophis marcianus*; Time period: distant future 2081–2100 (i.e., "2090" median); shared socio-economic pathway: "SSP126" (optimistic emissions-limiting models).

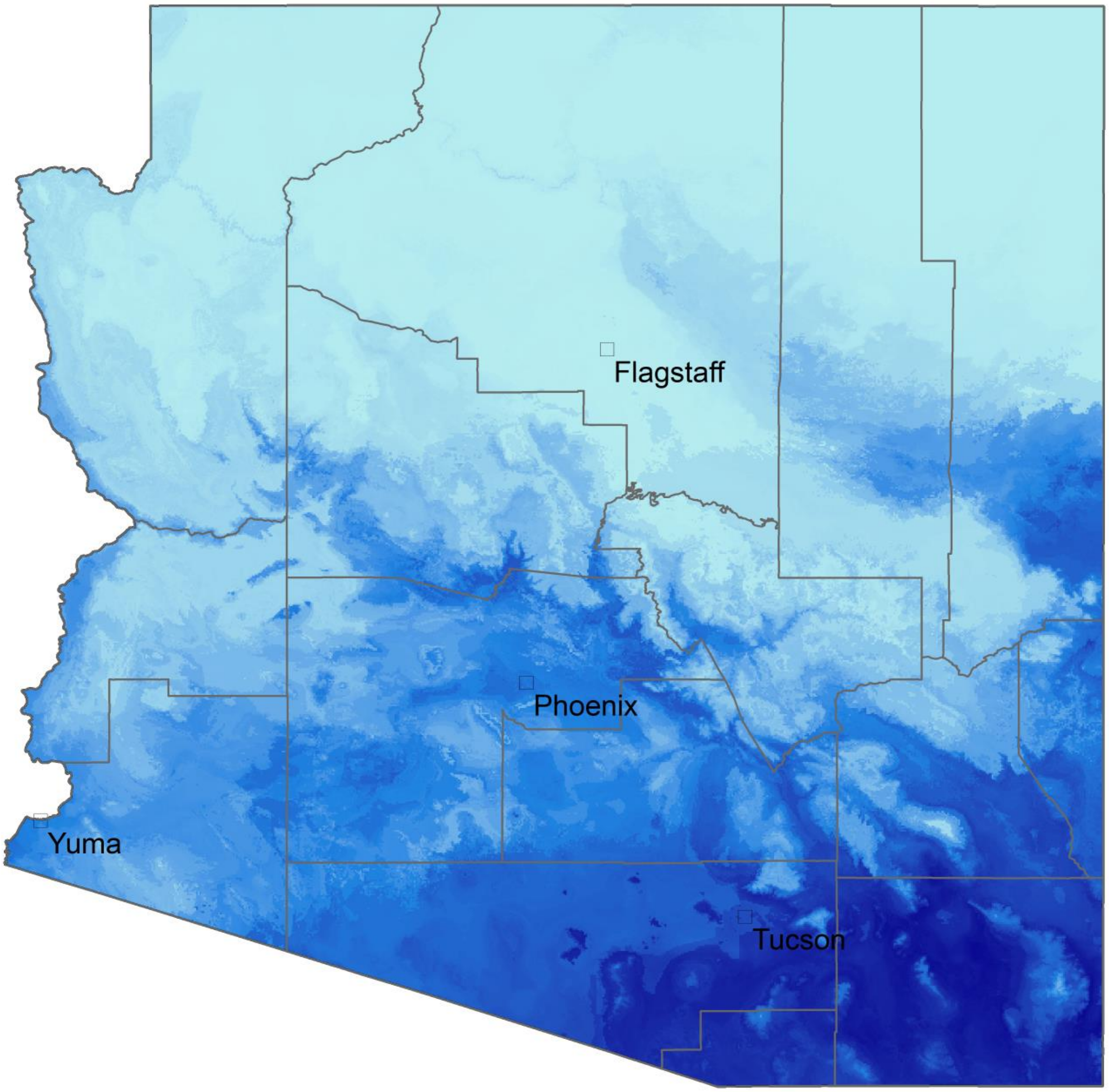

Fig S3t. Species: *Thamnophis marcianus*; Time period: distant future 2081–2100 (i.e., “2090” median); shared socio-economic pathway: “SSP585” (pessimistic ‘status quo’ emissions-limiting models).

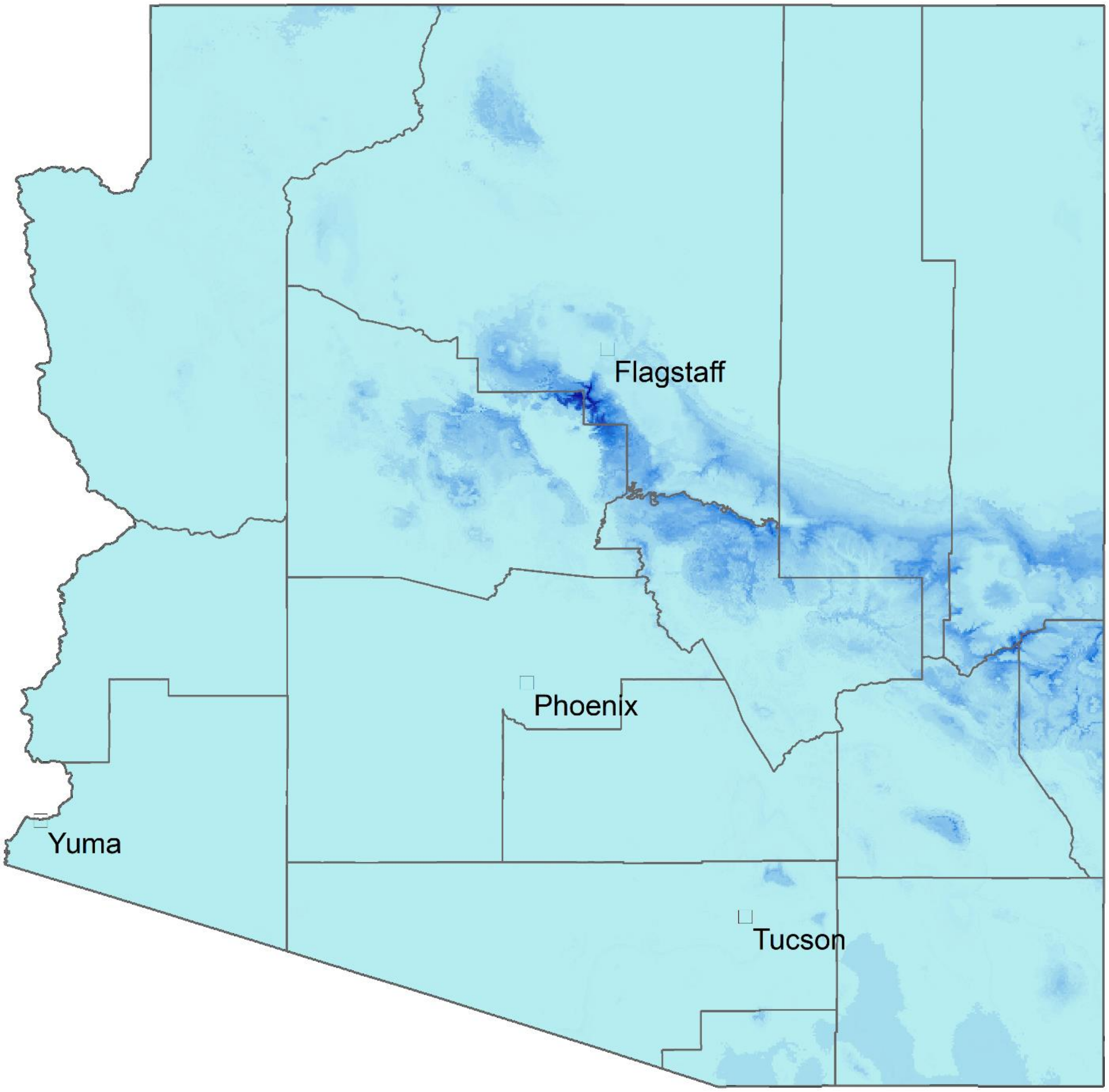

Fig S3u. Species: *Thamnophis rufipunctatus*; Time period: present (1980–2021); shared socio-economic pathway: n/a.

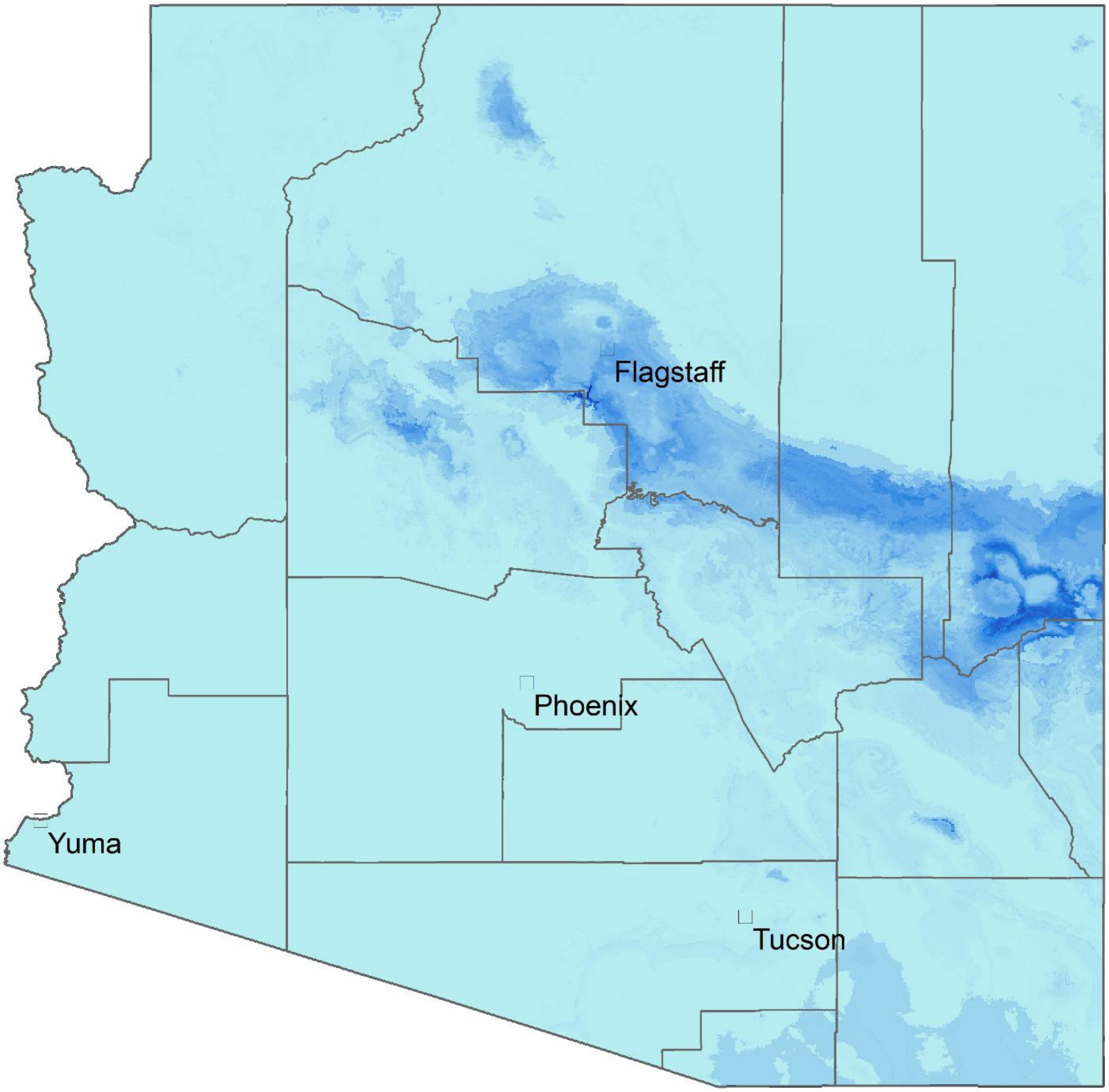

Fig S3v. Species: *Thamnophis rufipunctatus*; Time period: near future 2041–2060 (i.e., “2050” median); shared socio-economic pathway: “SSP126” (optimistic emissions-limiting models).

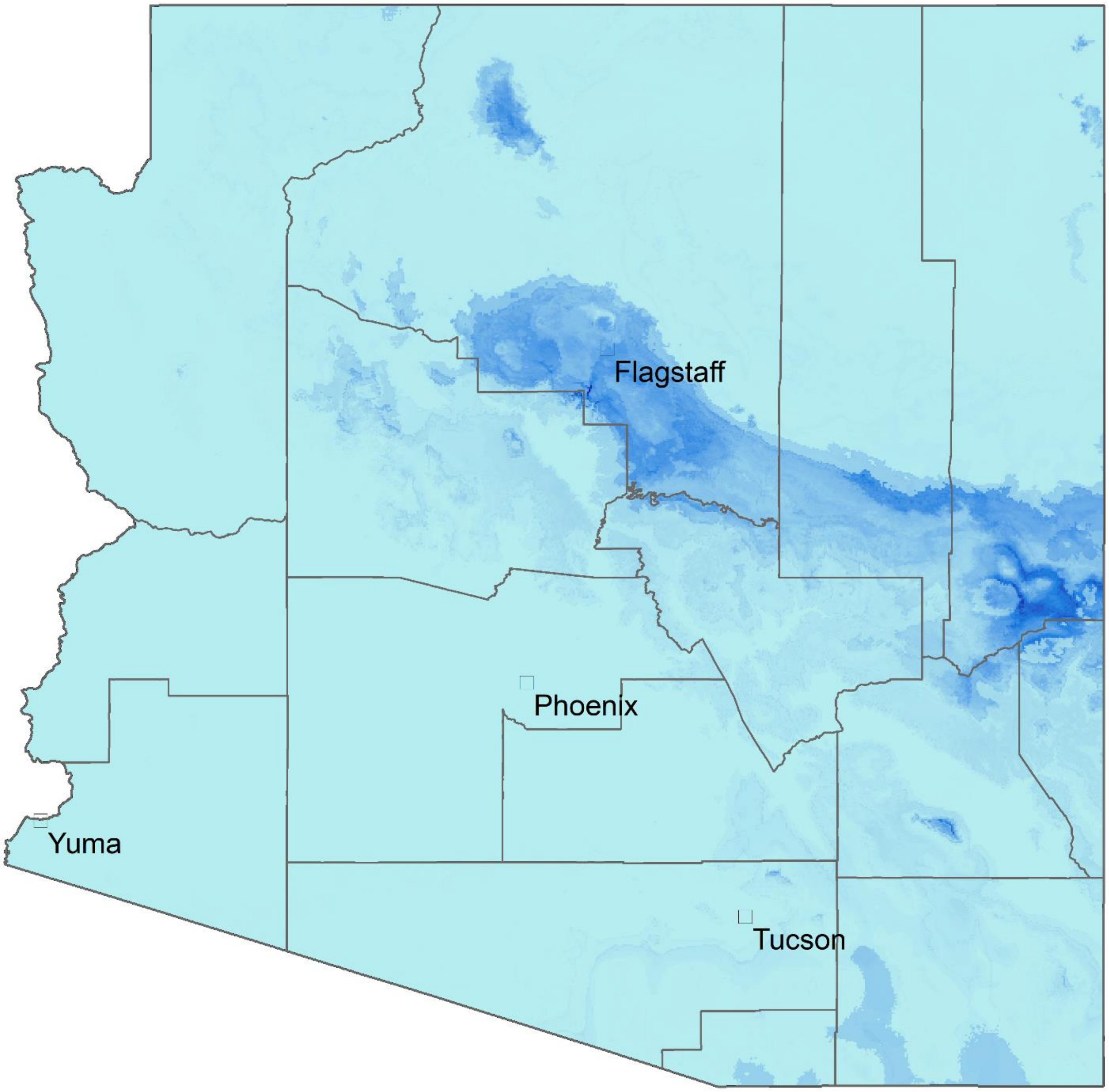

Fig S3w. Species: *Thamnophis rufipunctatus*; Time period: near future 2041–2060 (i.e., "2050" median); shared socio-economic pathway: "SSP585" (pessimistic 'status quo' emissions-limiting models).

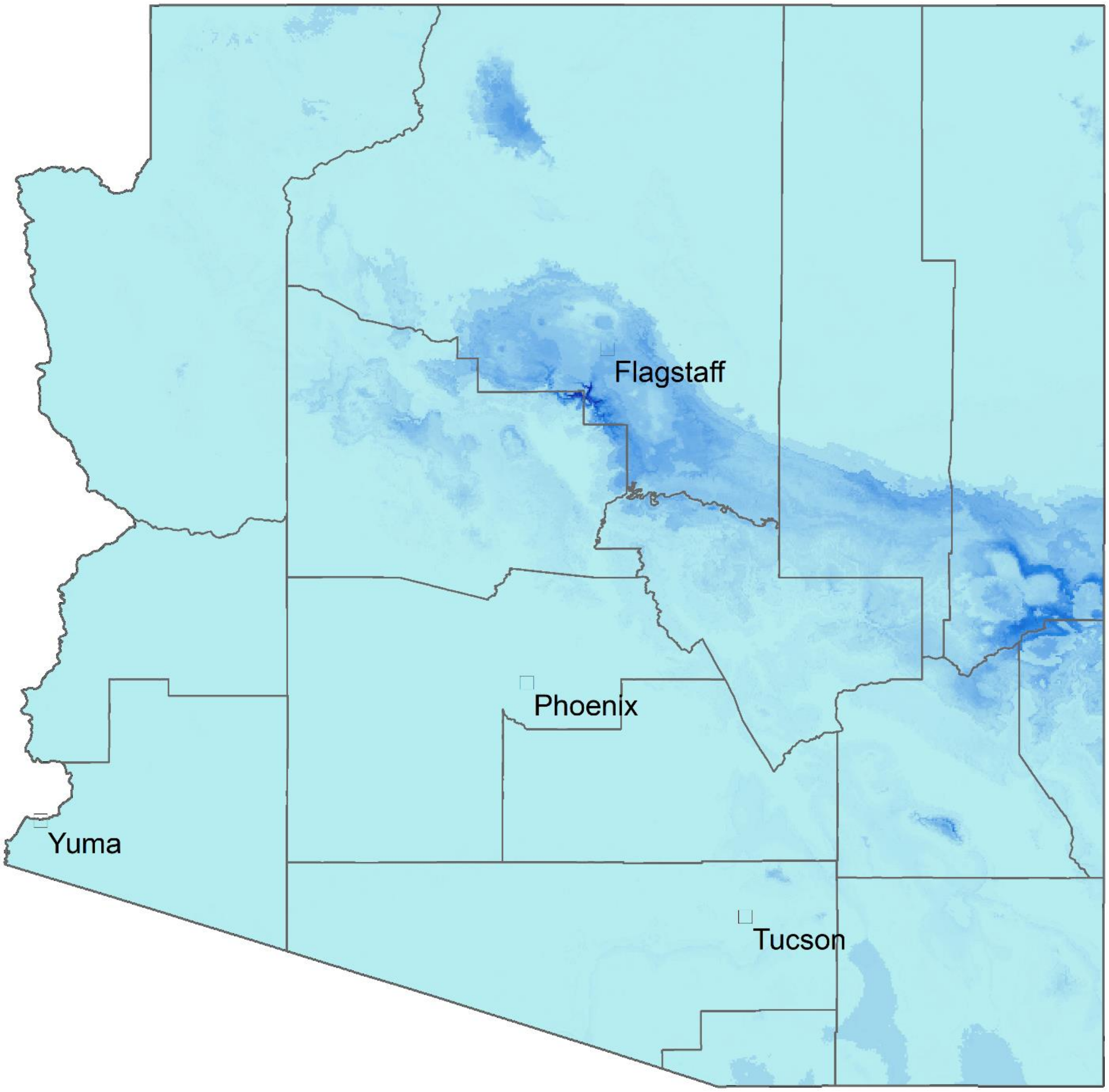

Fig S3x. Species: *Thamnophis rufipunctatus*; Time period: distant future 2081–2100 (i.e., “2090” median); shared socio-economic pathway: “SSP126” (optimistic emissions-limiting models).

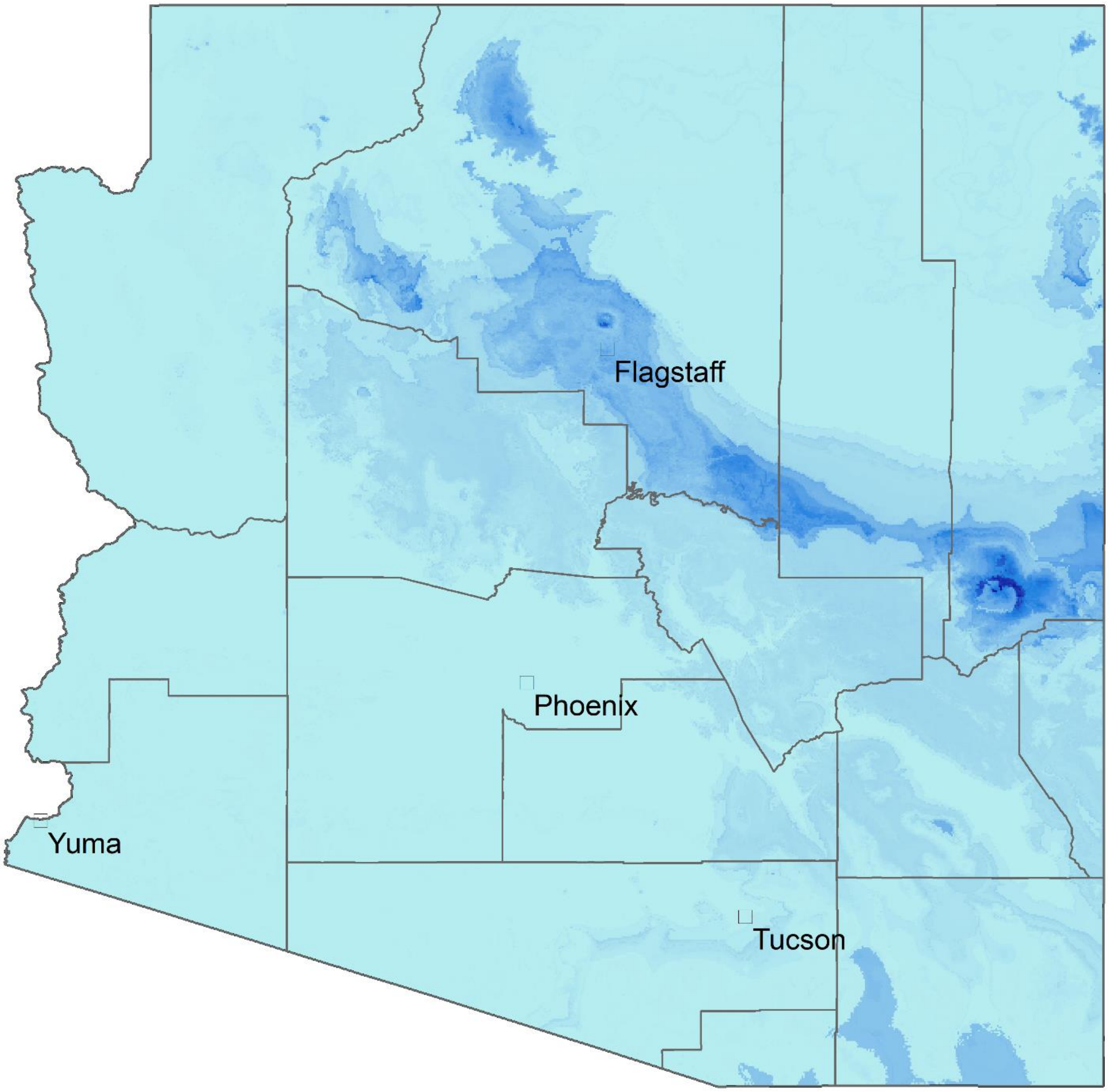

Fig S3y. Species: *Thamnophis rufipunctatus*; Time period: distant future 2081–2100 (i.e., "2090" median); shared socio-economic pathway: "SSP585" (pessimistic 'status quo' emissions-limiting models).
