# Supplementary material for: Modeling a hot, dry future: Substantial range reductions in suitable environment projected under climate change for a semiarid riparian predator guild
Source: PLoS One. 2024 May 6;19(5):e0302981. doi: 10.1371/journal.pone.0302981 (PMC11073737; doi:10.1371/journal.pone.0302981)
Supplement: S4 Fig — Data are modeled from weighted-means ensemble models generated from the MRI-ESM2-0 global climate model. Changes based on present time modeling (1980–2021). Color values: yellow = suitable, stable; blue = suitable gained; red = lost, unsuitable; gray = unsuitable. (PDF) [file pone.0302981.s004.pdf]

Fig S4. Individual panels (a–t) from 'Fig 4 Projected change in range sizes for *Thamnophis* gartersnakes in Arizona.' Data are modeled from weighted-means ensemble models generated from the MRI-ESM2-0 global climate model. Changes based on present time (1980–2021) modeling. Color values: yellow = suitable, stable; blue = suitable gained; red = lost, unsuitable; gray = unsuitable.

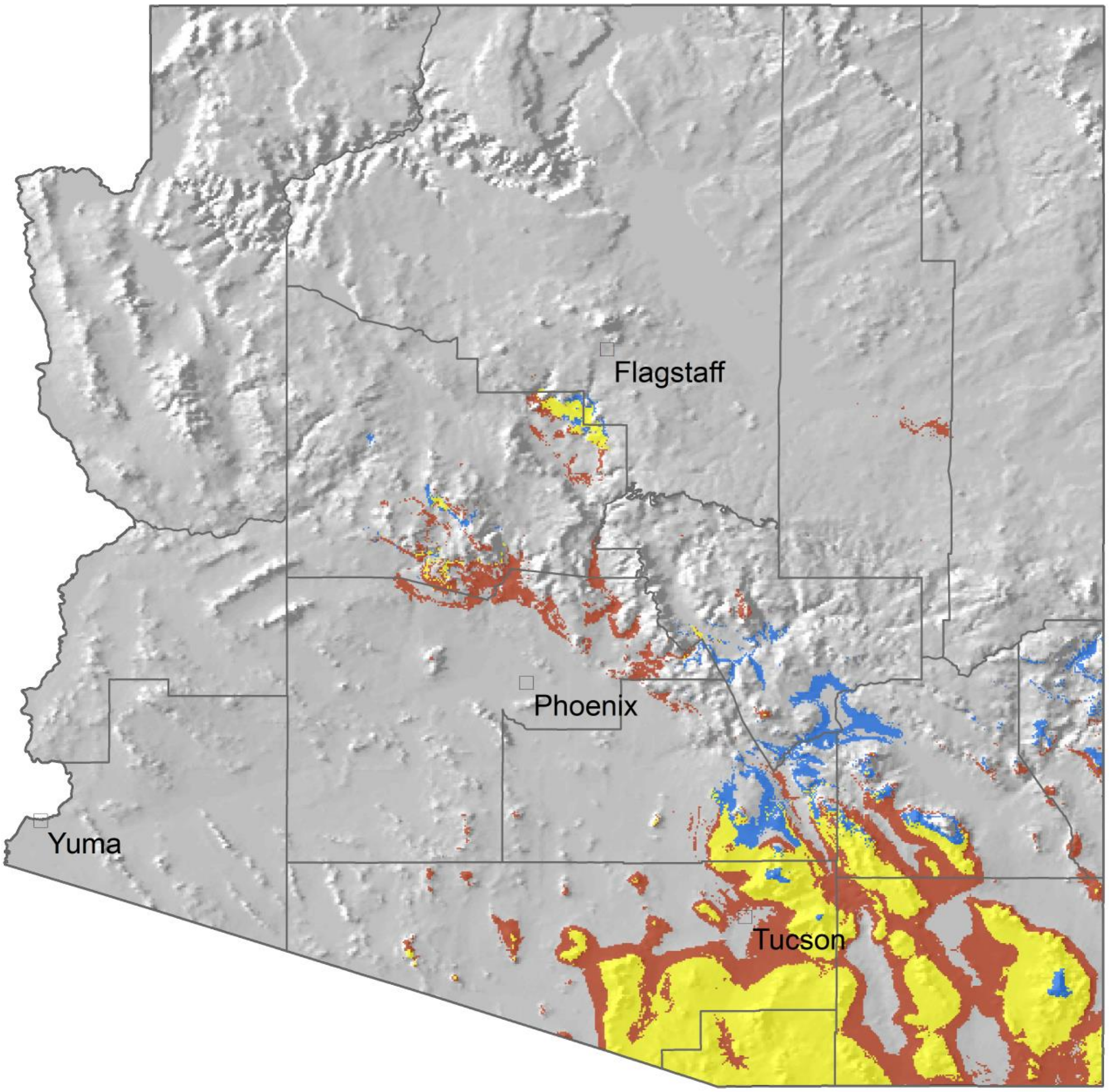

Fig S4a. Species: *Thamnophis cyrtopsis*; Time period: near future 2041–2060 (i.e., "2050" median); shared socio-economic pathway: "SSP126" (optimistic emissions-limiting models).

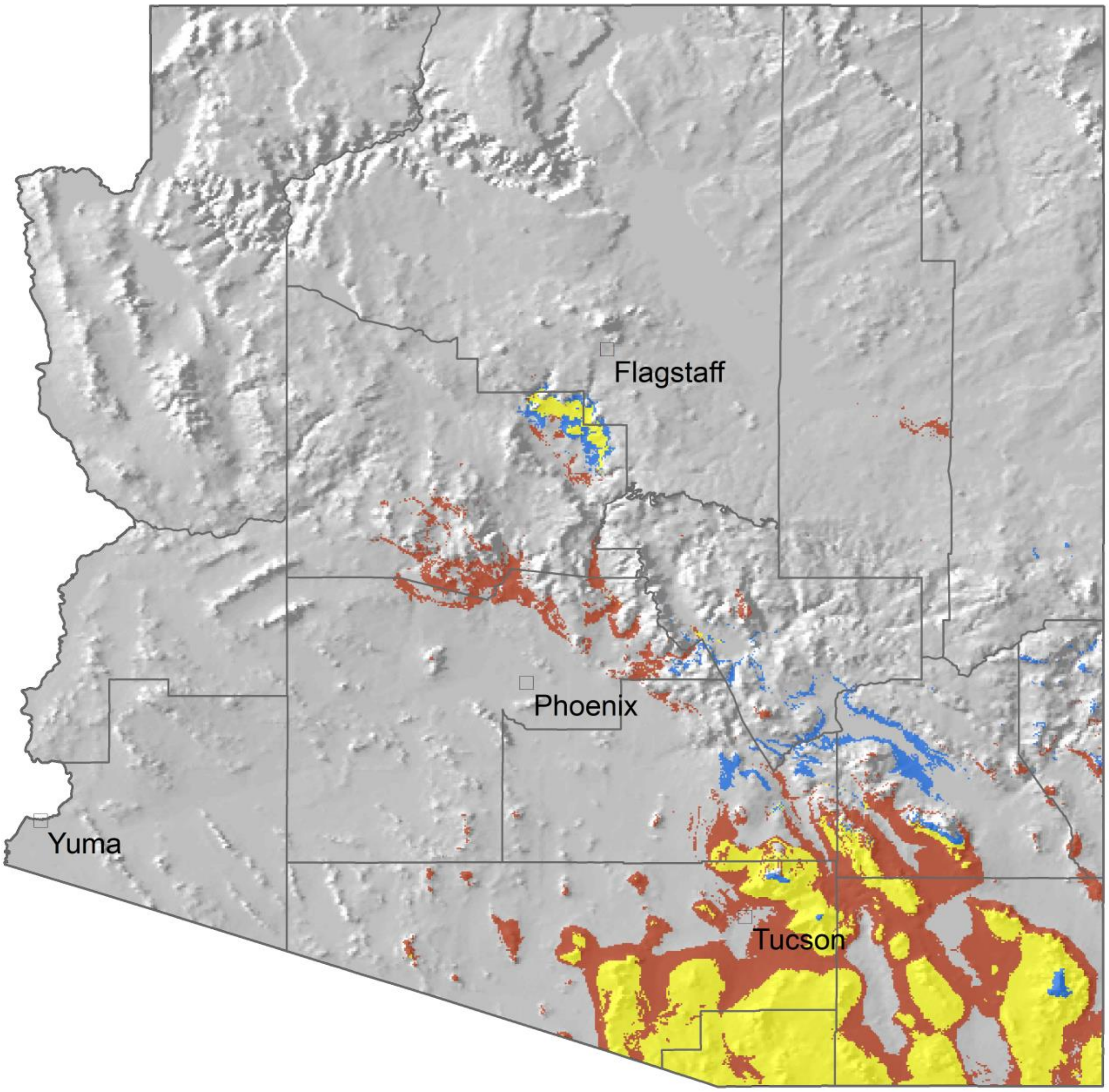

Fig S4b. Species: *Thamnophis cyrtopsis*; Time period: near future 2041–2060 (i.e., “2050” median); shared socio-economic pathway: “SSP585” (pessimistic ‘status quo’ emissions-limiting models).

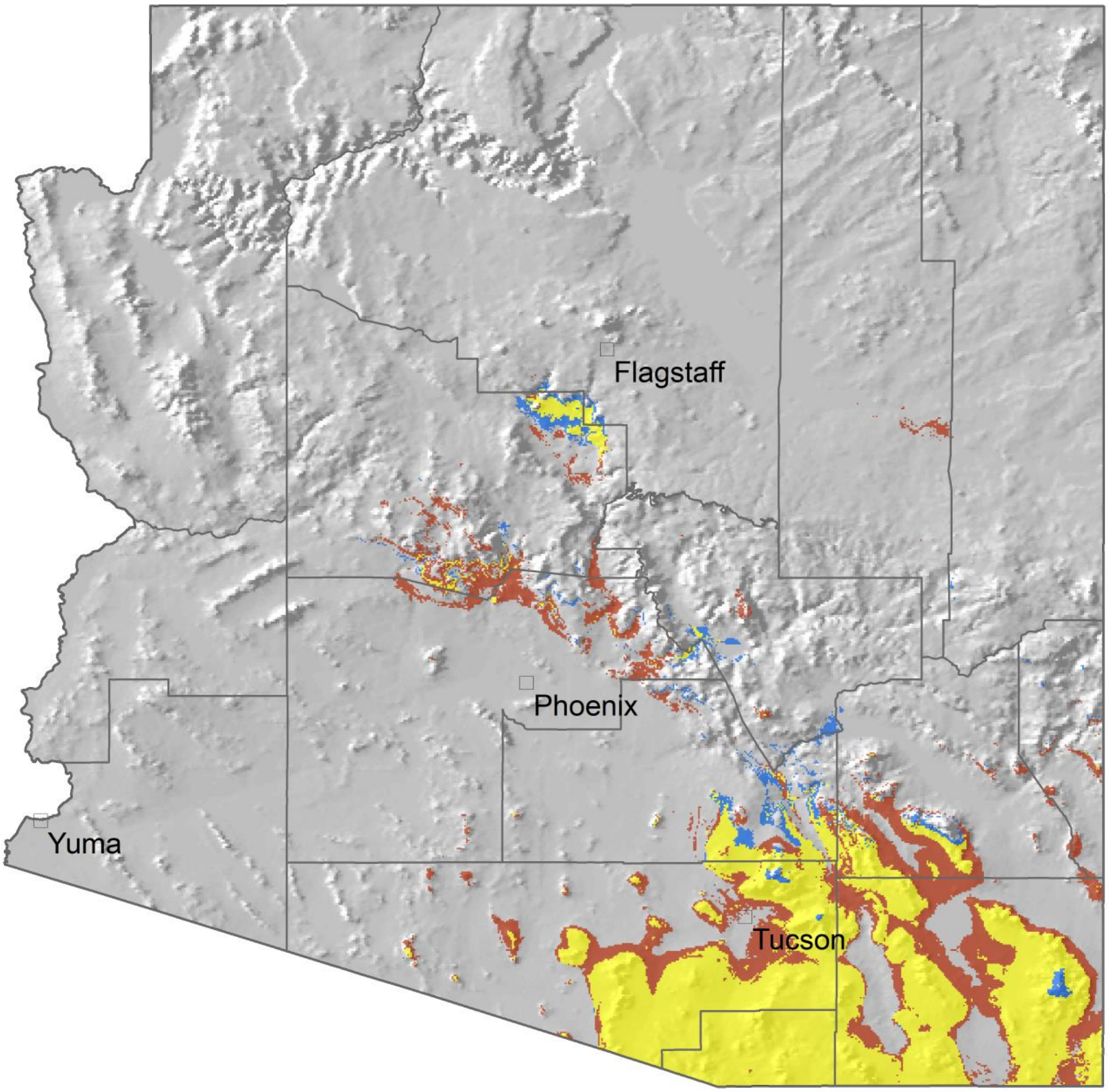

Fig S4c. Species: *Thamnophis cyrtopsis*; Time period: distant future 2081–2100 (i.e., “2090” median); shared socio-economic pathway: “SSP126” (optimistic emissions-limiting models).

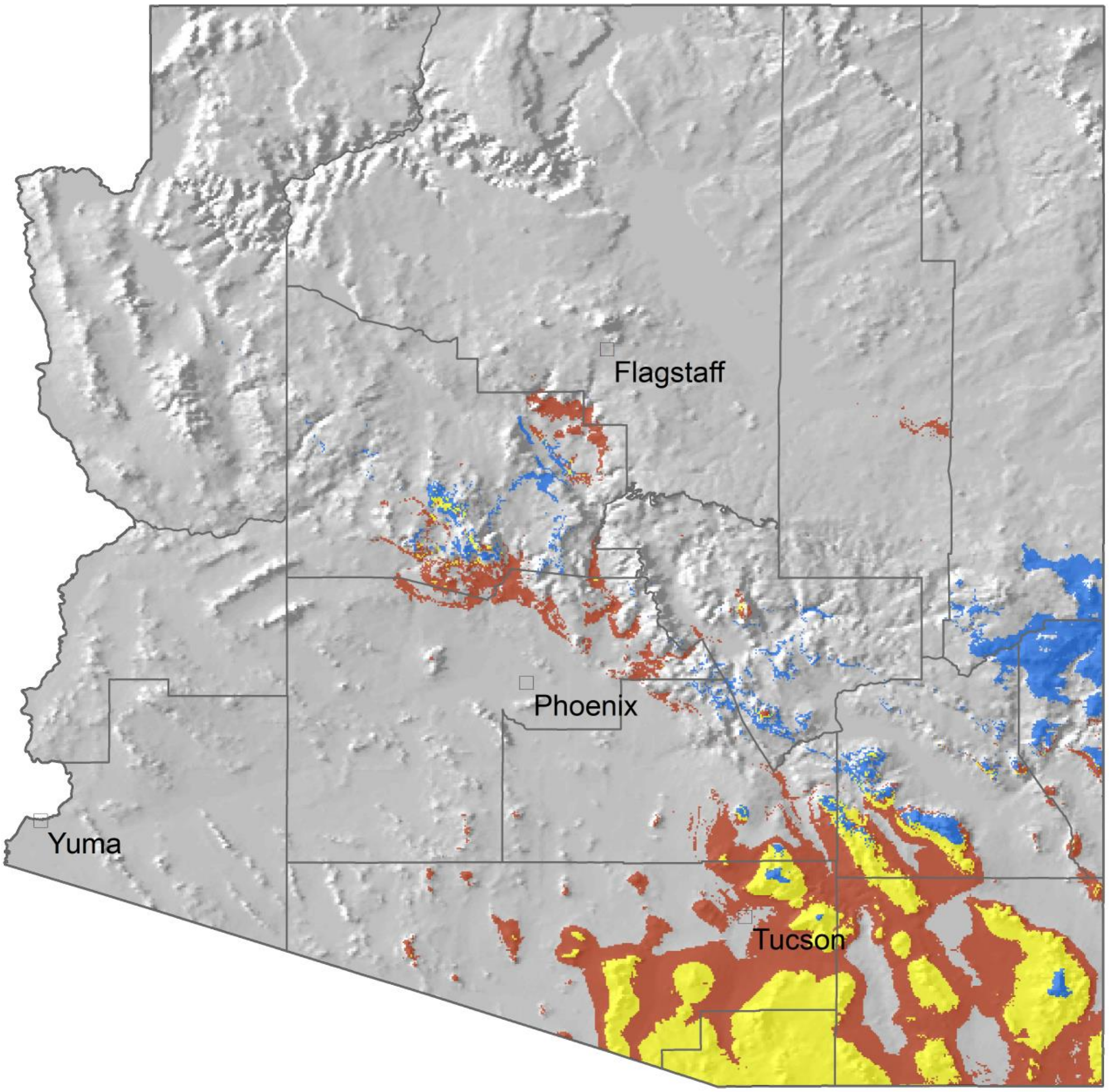

Fig S4d. Species: *Thamnophis cyrtopsis*; Time period: distant future 2081–2100 (i.e., “2090” median); shared socio-economic pathway: “SSP585” (pessimistic ‘status quo’ emissions-limiting models).

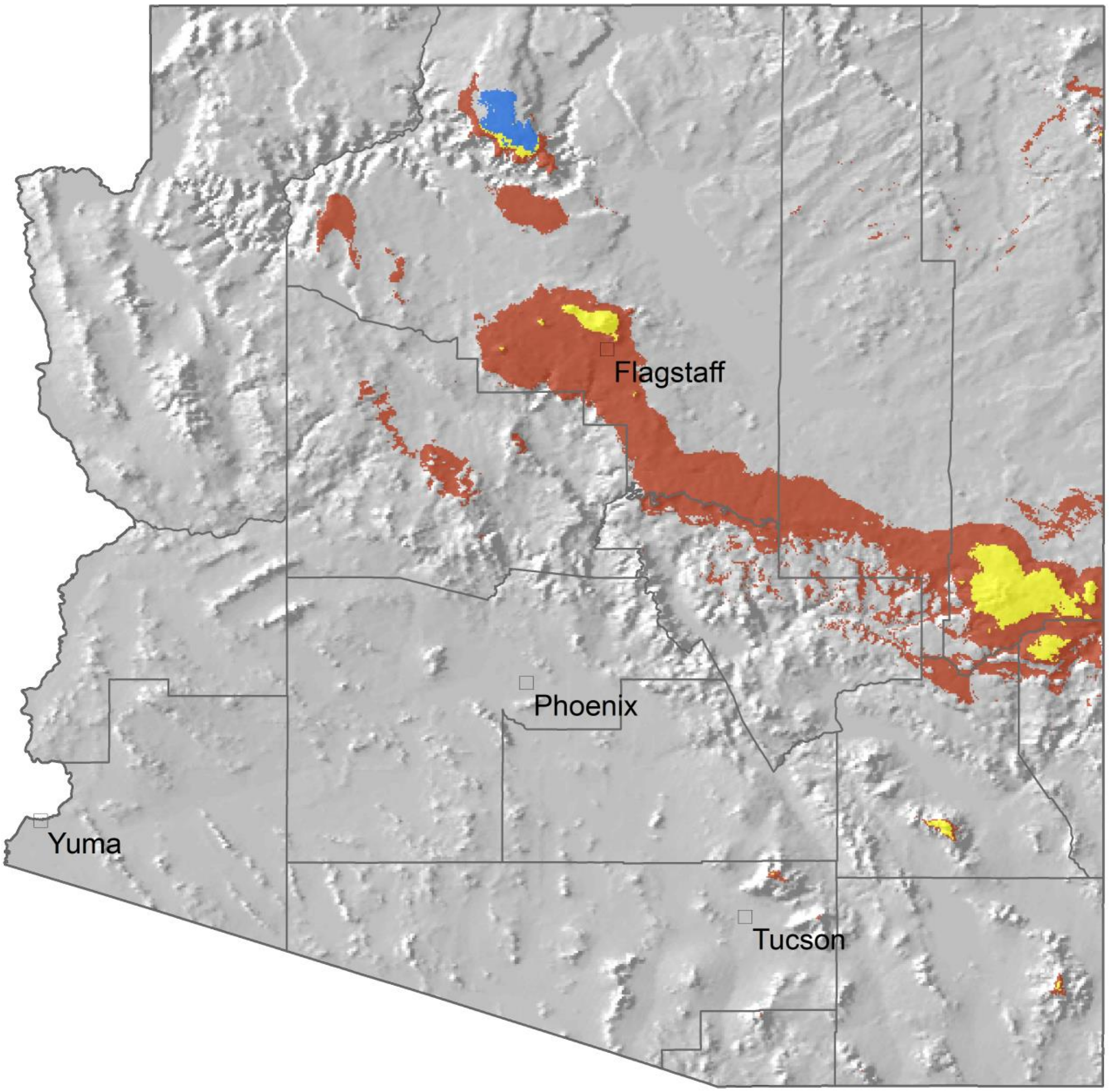

Fig S4e. Species: *Thamnophis elegans*; Time period: near future 2041–2060 (i.e., “2050” median); shared socio-economic pathway: “SSP126” (optimistic emissions-limiting models).

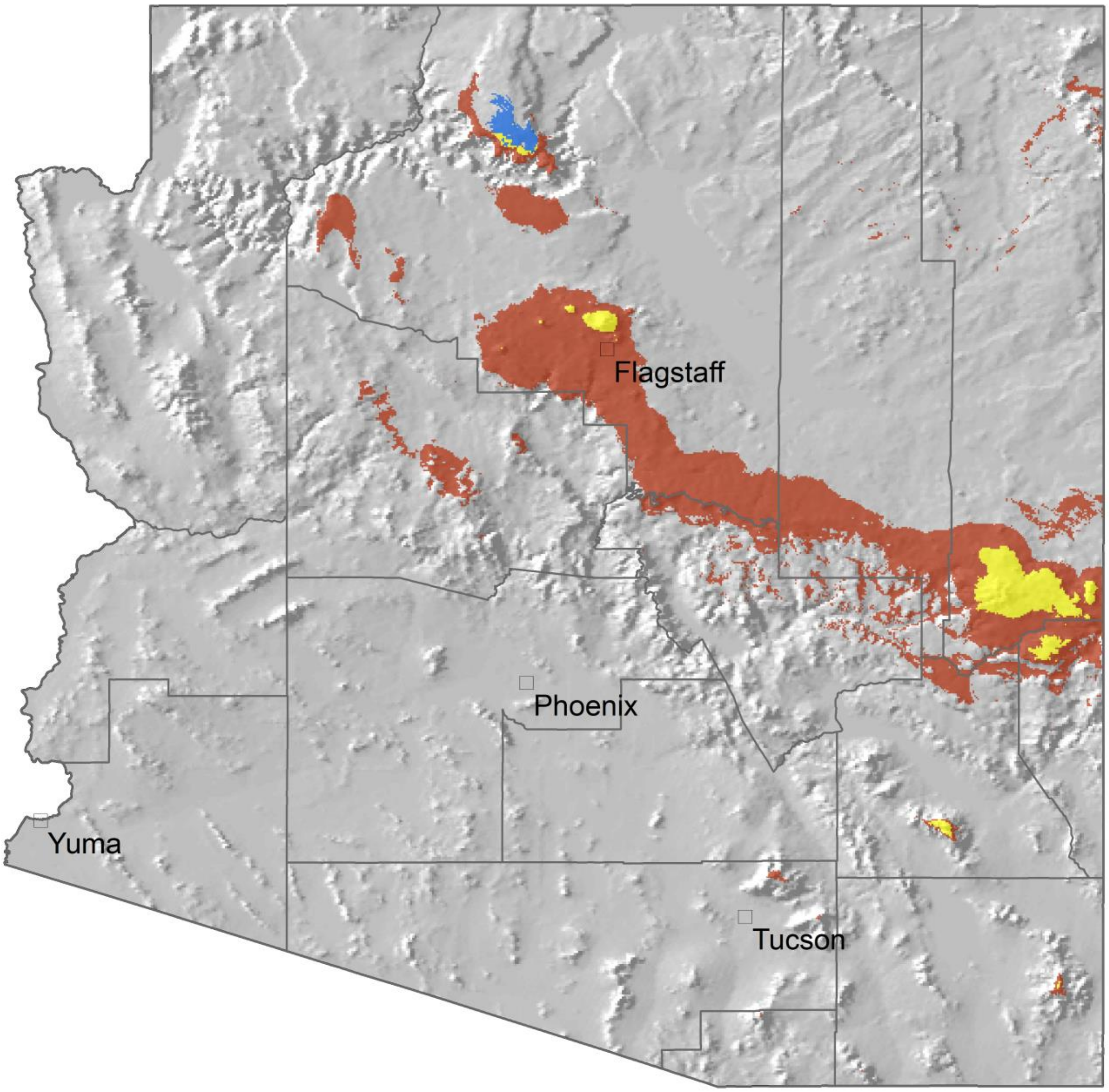

Fig S4f. Species: *Thamnophis elegans*; Time period: near future 2041–2060 (i.e., “2050” median); shared socio-economic pathway: “SSP585” (pessimistic ‘status quo’ emissions-limiting models).

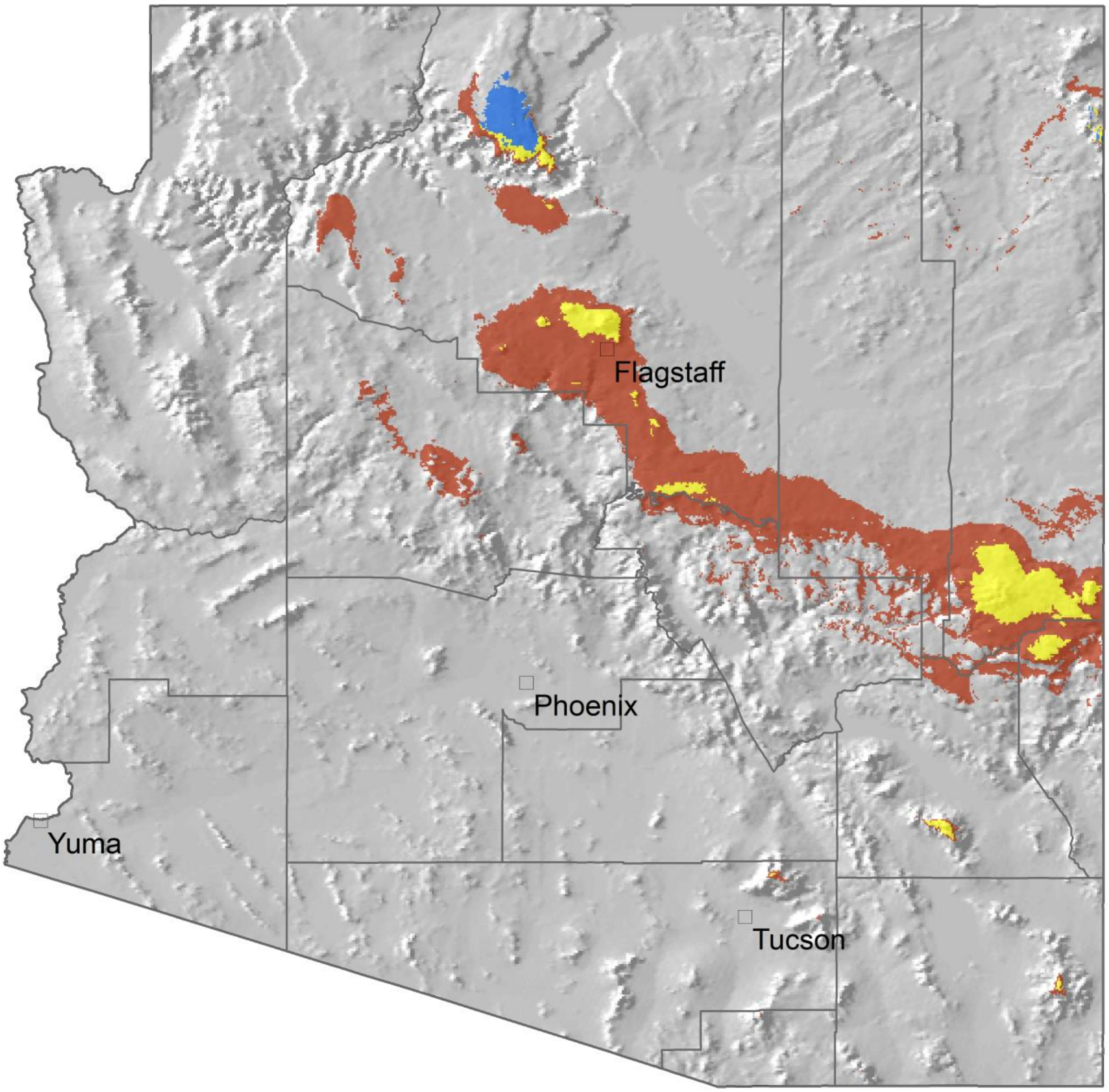

Fig S4g. Species: *Thamnophis elegans*; Time period: distant future 2081–2100 (i.e., “2090” median); shared socio-economic pathway: “SSP126” (optimistic emissions-limiting models).

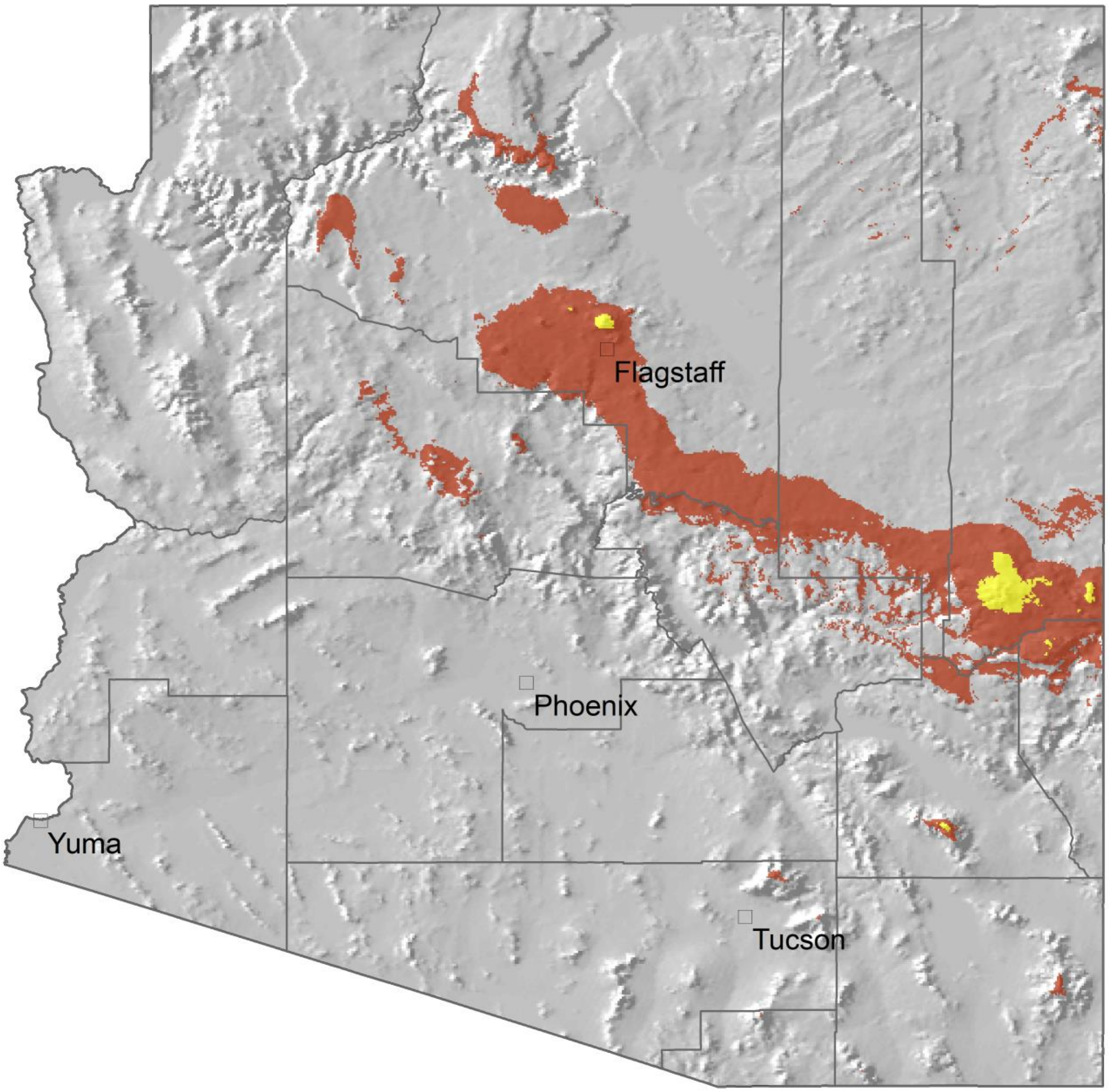

Fig S4h. Species: *Thamnophis elegans*; Time period: distant future 2081–2100 (i.e., “2090” median); shared socio-economic pathway: “SSP585” (pessimistic ‘status quo’ emissions-limiting models).

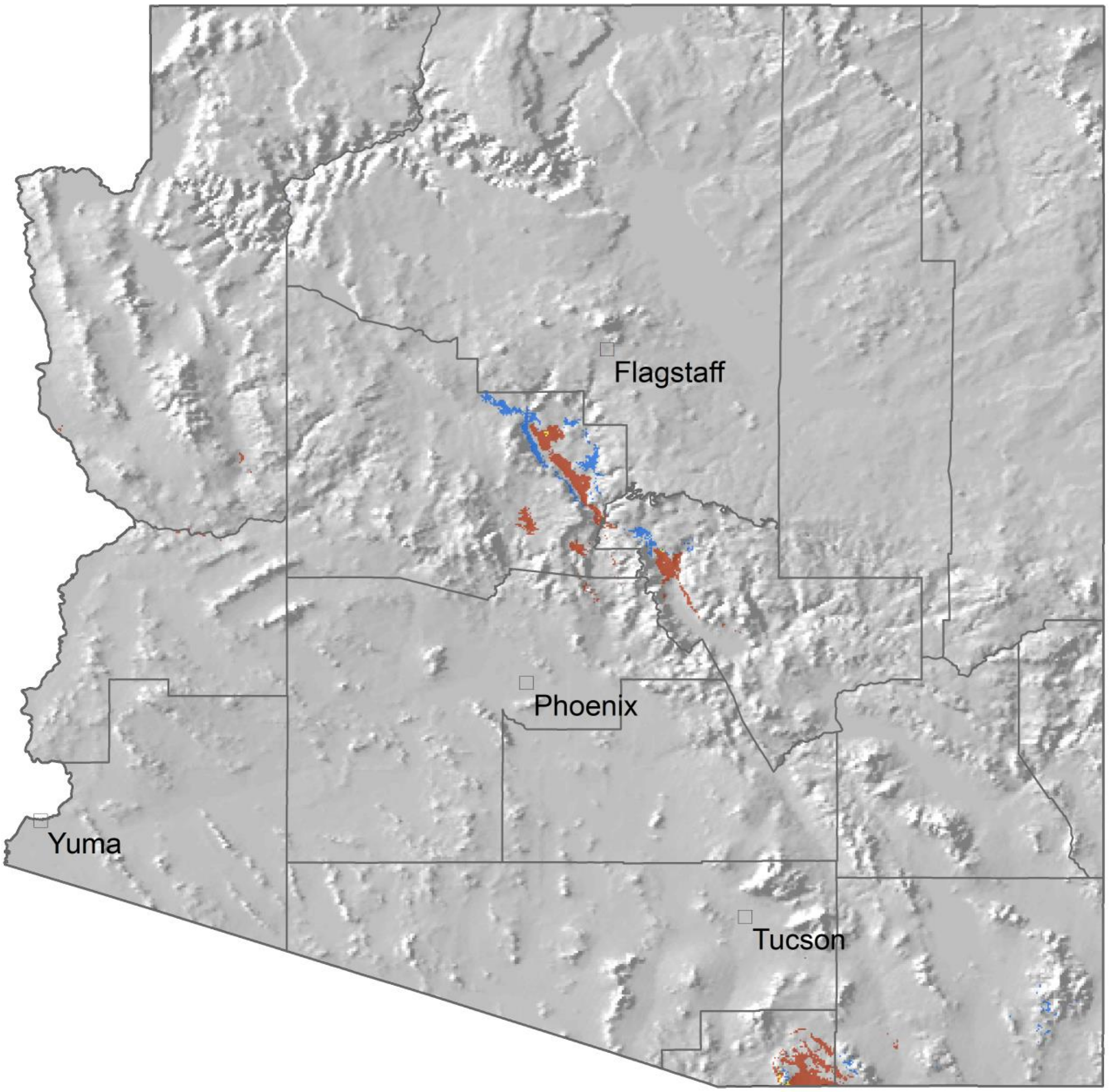

Fig S4i. Species: *Thamnophis eques*; Time period: near future 2041–2060 (i.e., “2050” median); shared socio-economic pathway: “SSP126” (optimistic emissions-limiting models).

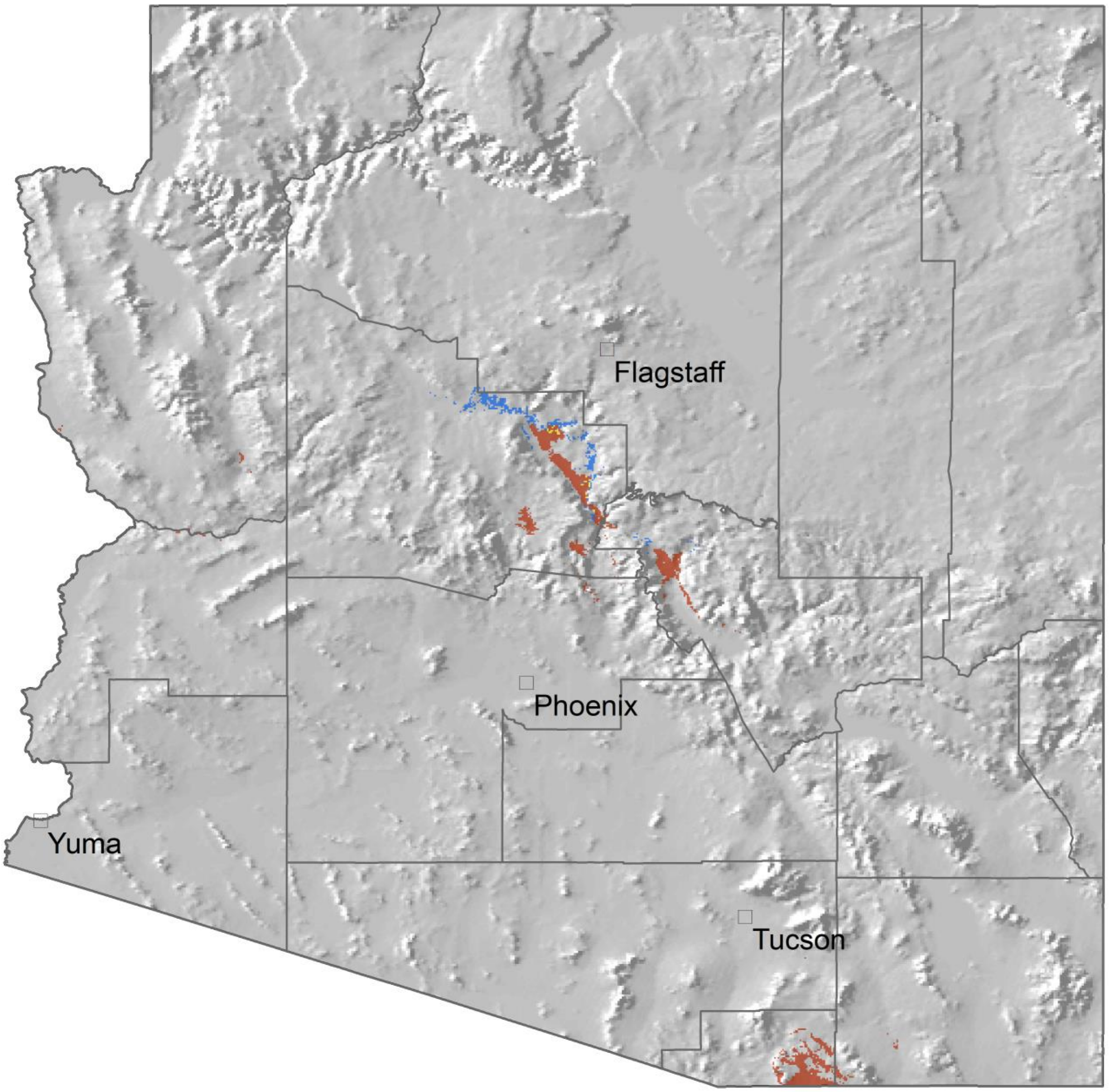

Fig S4j. Species: *Thamnophis eques*; Time period: near future 2041–2060 (i.e., “2050” median); shared socio-economic pathway: “SSP585” (pessimistic ‘status quo’ emissions-limiting models).

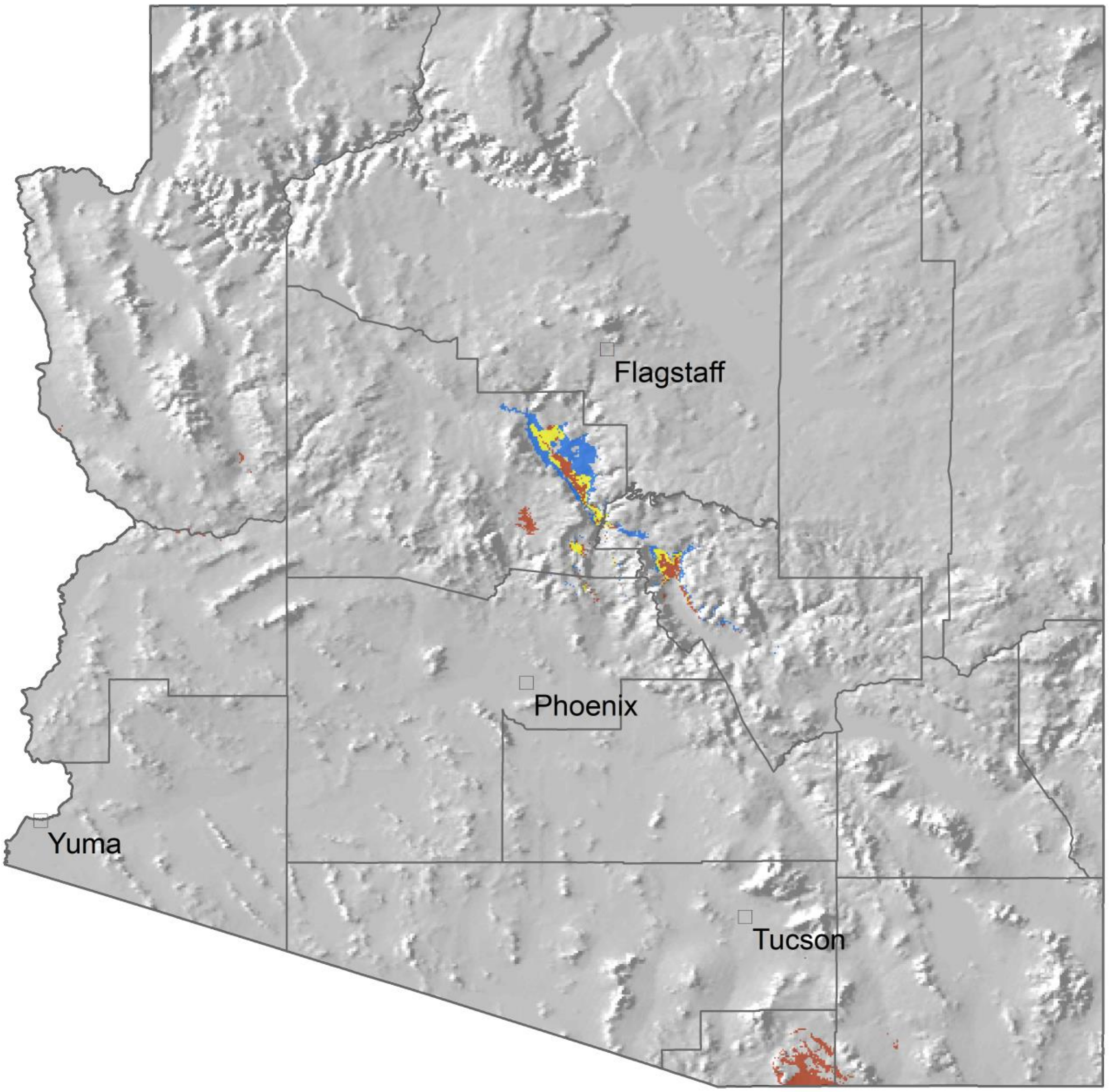

Fig S4k. Species: *Thamnophis eques*; Time period: distant future 2081–2100 (i.e., “2090” median); shared socio-economic pathway: “SSP126” (optimistic emissions-limiting models).

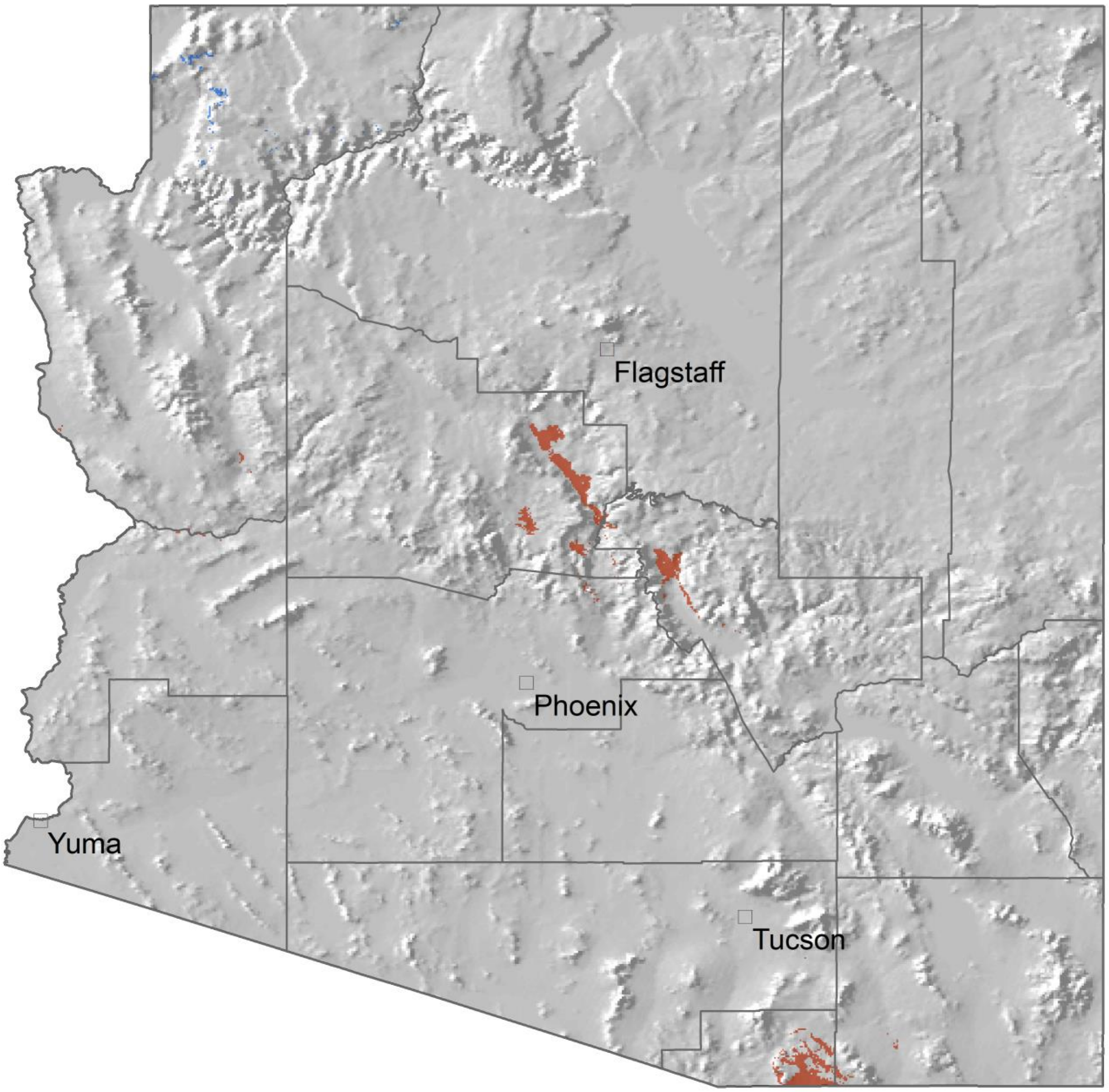

Fig S4l. Species: *Thamnophis eques*; Time period: distant future 2081–2100 (i.e., “2090” median); shared socio-economic pathway: “SSP585” (pessimistic ‘status quo’ emissions-limiting models).

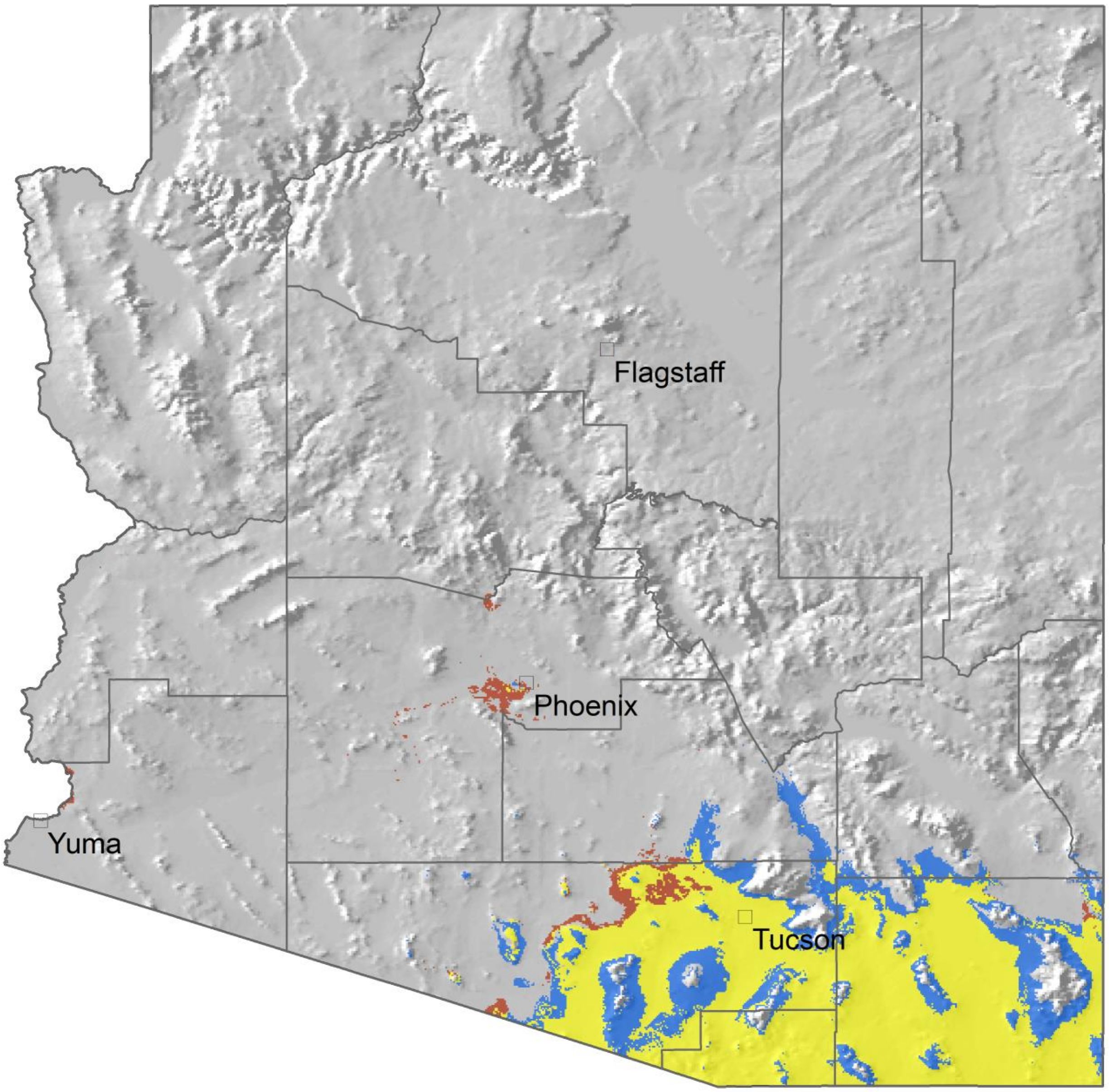

Fig S4m. Species: *Thamnophis marcianus*; Time period: near future 2041–2060 (i.e., “2050” median); shared socio-economic pathway: “SSP126” (optimistic emissions-limiting models).

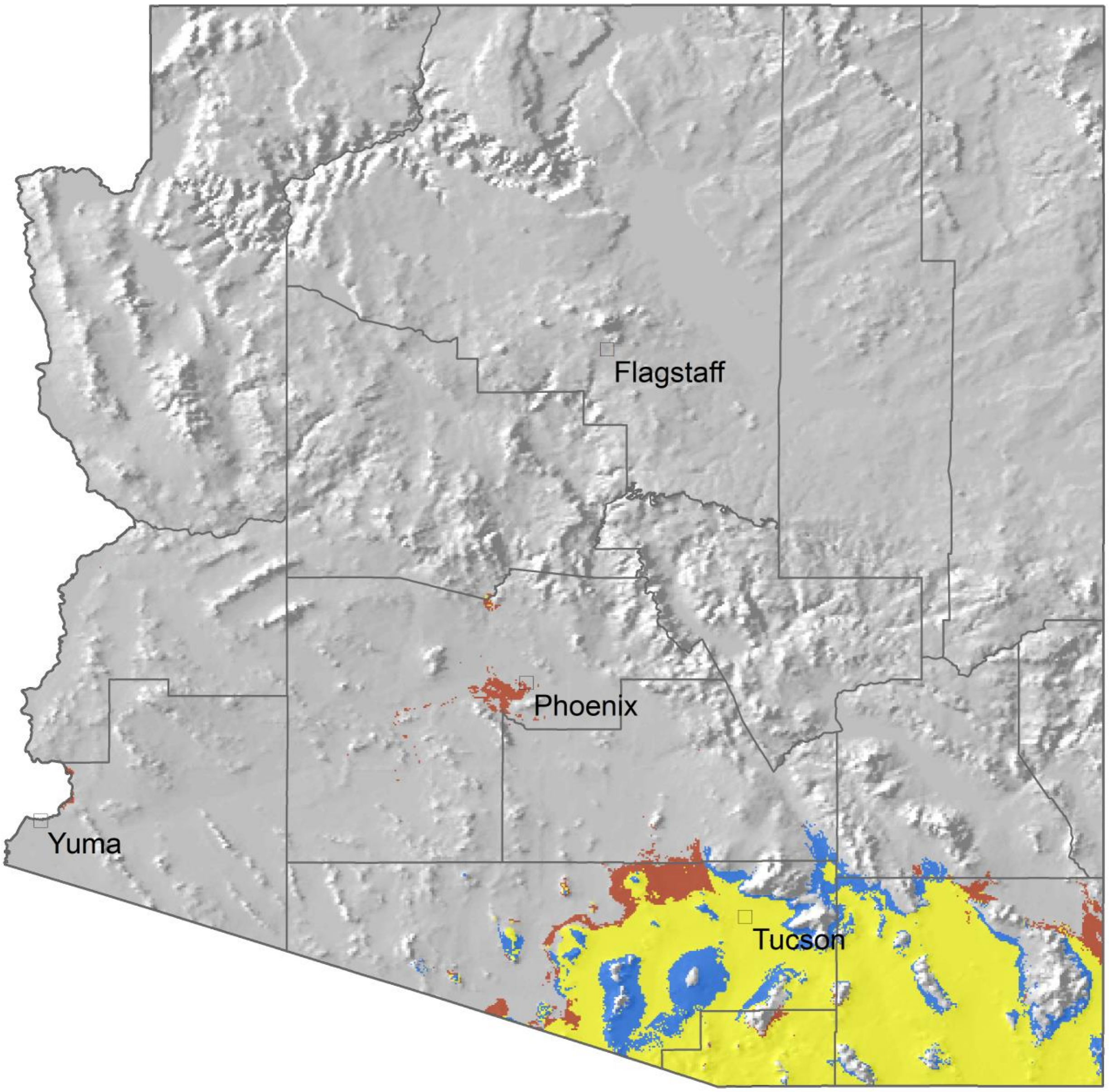

Fig S4n. Species: *Thamnophis marcianus*; Time period: near future 2041–2060 (i.e., "2050" median); shared socio-economic pathway: "SSP585" (pessimistic 'status quo' emissions-limiting models).

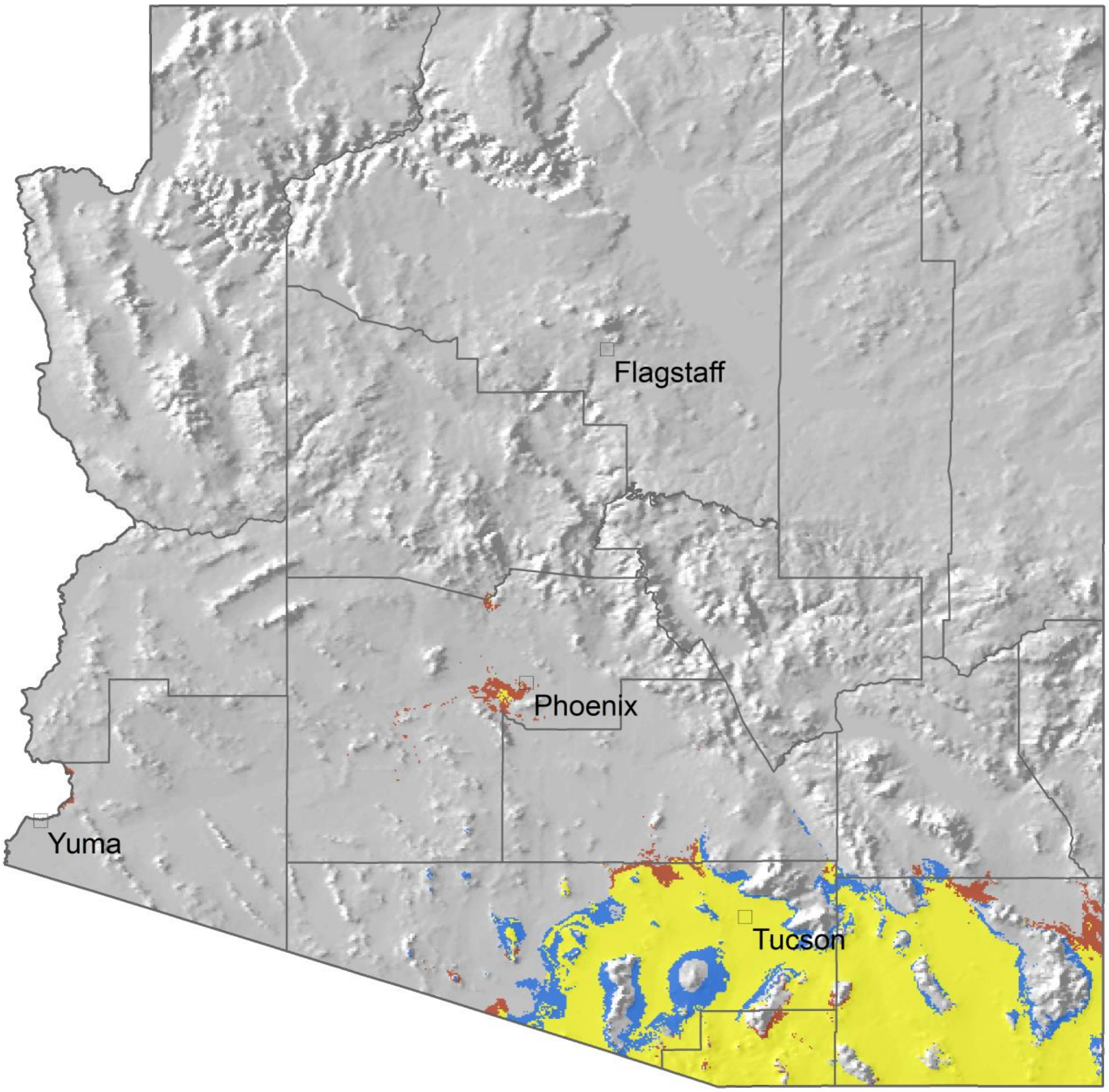

Fig S4o. Species: *Thamnophis marcianus*; Time period: distant future 2081–2100 (i.e., “2090” median); shared socio-economic pathway: “SSP126” (optimistic emissions-limiting models).

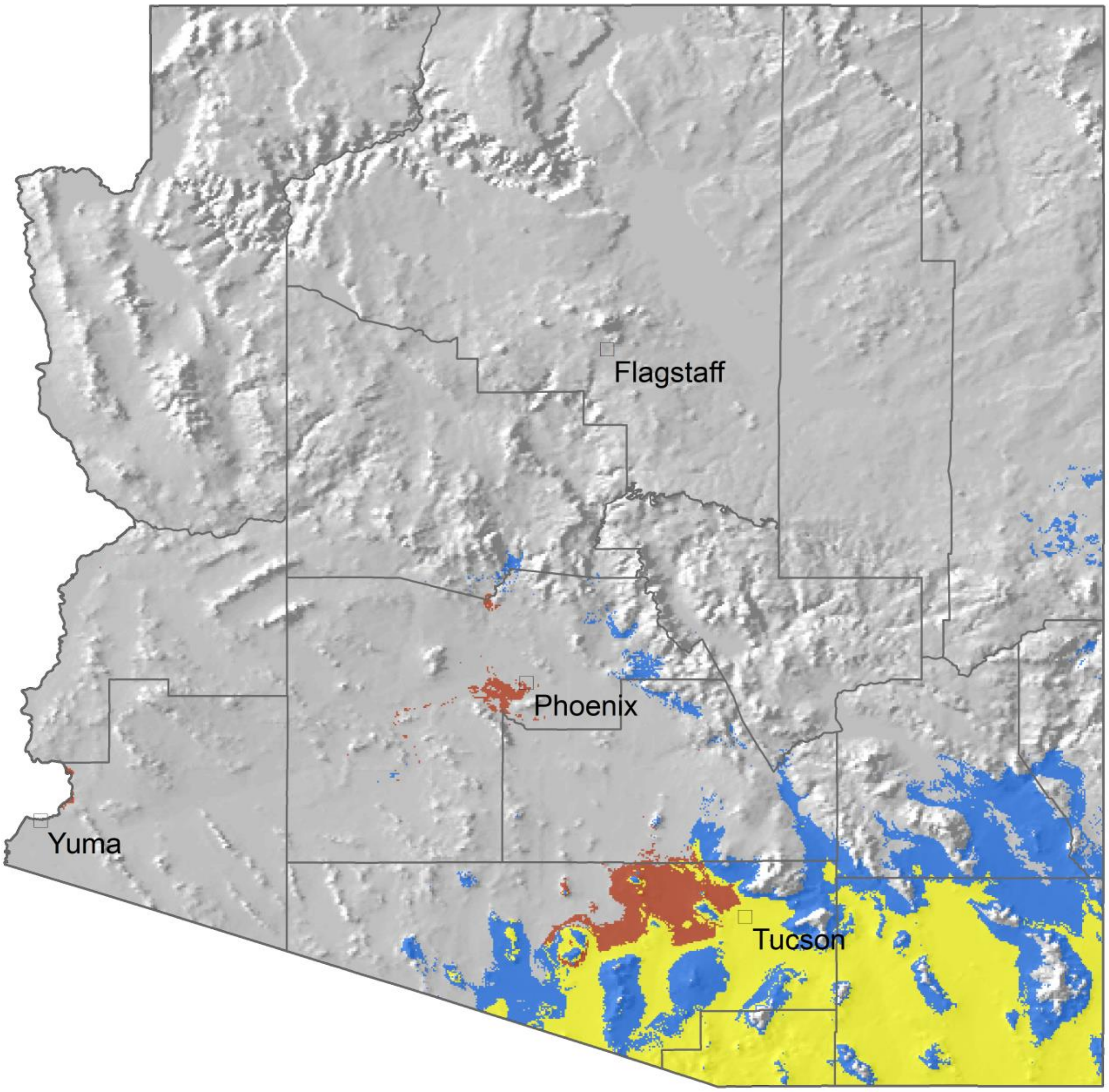

Fig S4p. Species: *Thamnophis marcianus*; Time period: distant future 2081–2100 (i.e., “2090” median); shared socio-economic pathway: “SSP585” (pessimistic ‘status quo’ emissions-limiting models).

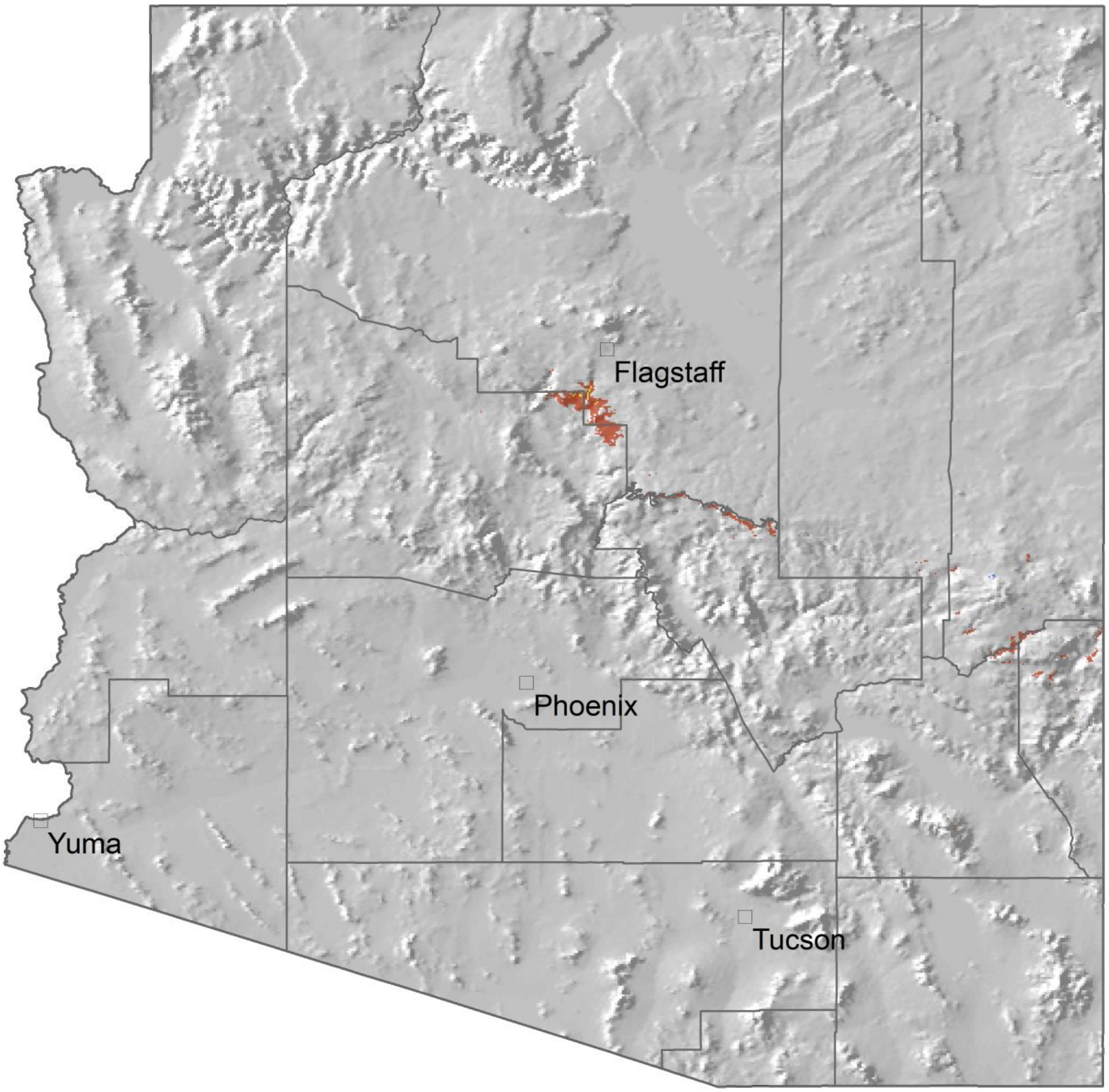

Fig S4q. Species: *Thamnophis rufipunctatus*; Time period: near future 2041–2060 (i.e., “2050” median); shared socio-economic pathway: “SSP126” (optimistic emissions-limiting models).

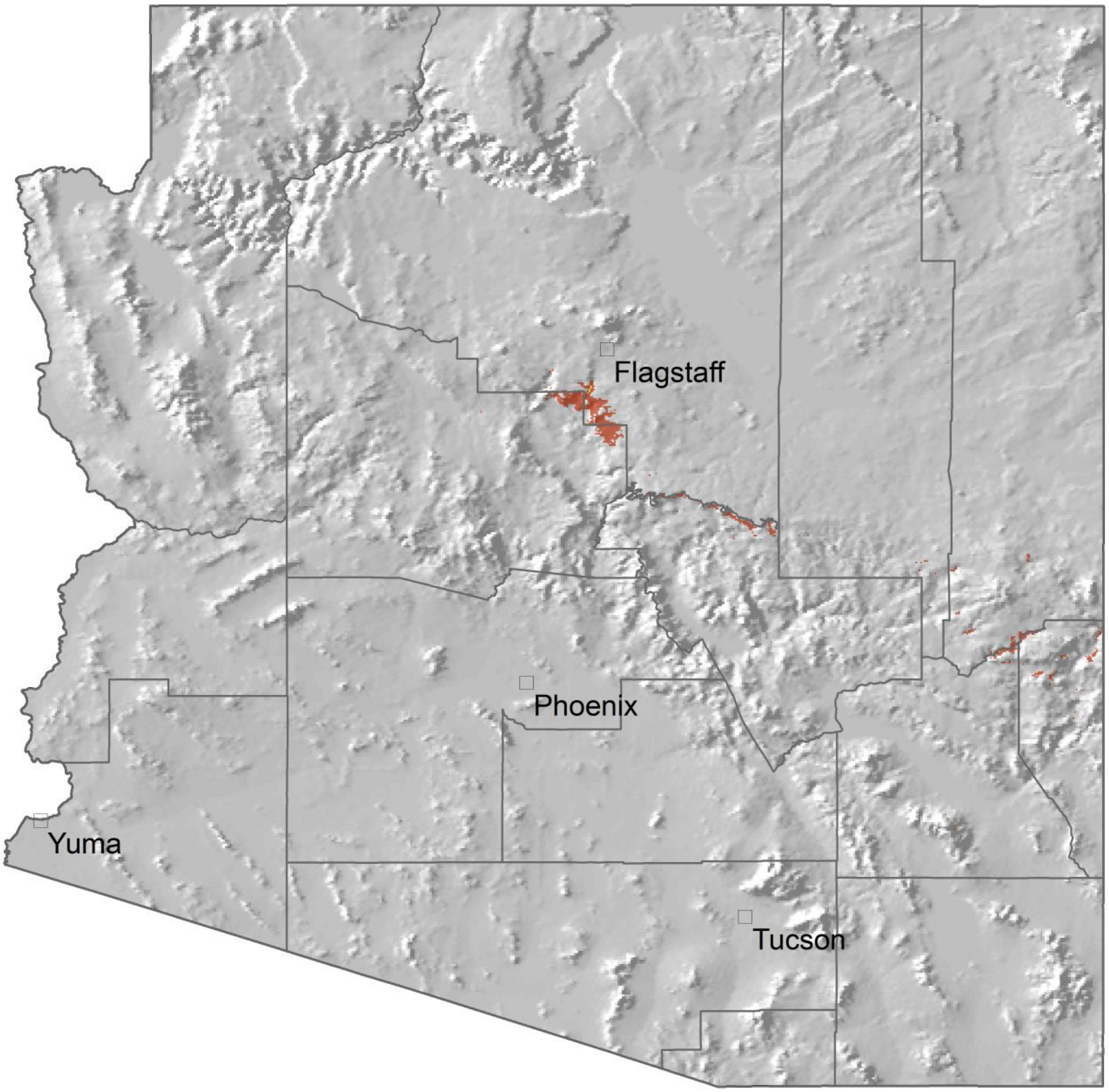

Fig S4r. Species: *Thamnophis rufipunctatus*; Time period: near future 2041–2060 (i.e., "2050" median); shared socio-economic pathway: "SSP585" (pessimistic 'status quo' emissions-limiting models).

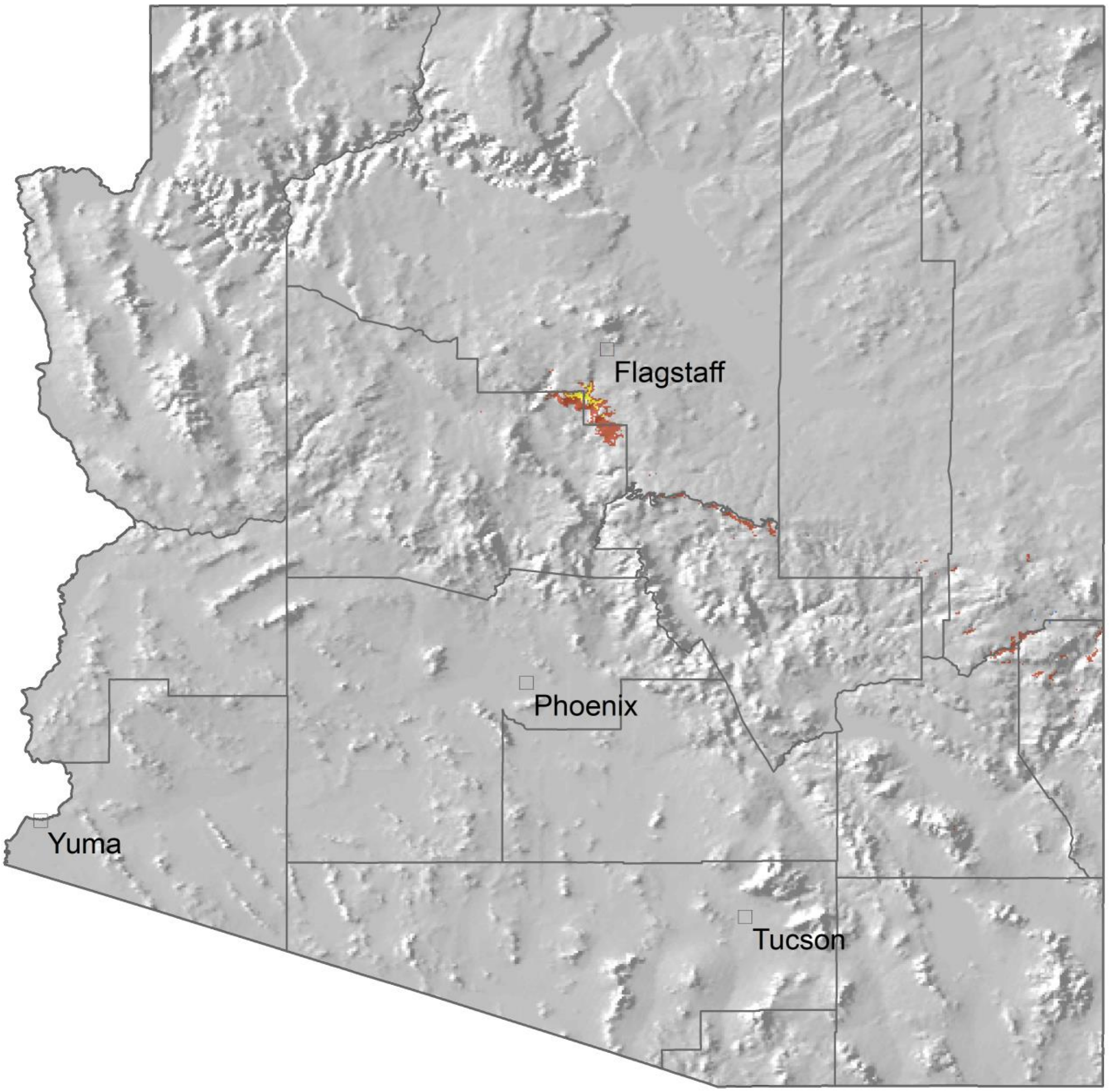

Fig S4s. Species: *Thamnophis rufipunctatus*; Time period: distant future 2081–2100 (i.e., “2090” median); shared socio-economic pathway: “SSP126” (optimistic emissions-limiting models).

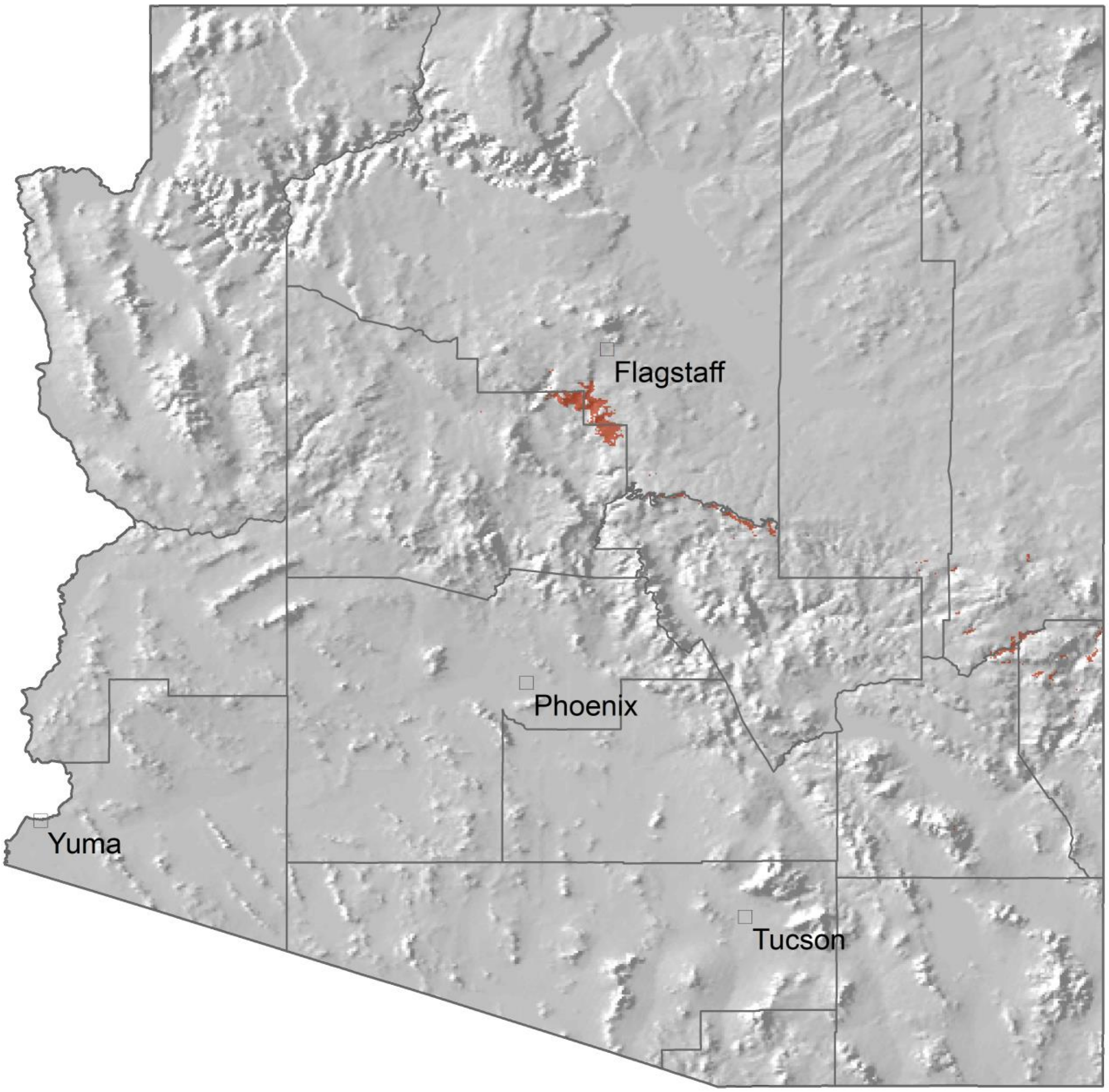

Fig S4t. Species: *Thamnophis rufipunctatus*; Time period: distant future 2081–2100 (i.e., "2090" median); shared socio-economic pathway: "SSP585" (pessimistic 'status quo' emissions-limiting models).
