# Supplementary material for: Modeling a hot, dry future: Substantial range reductions in suitable environment projected under climate change for a semiarid riparian predator guild
Source: PLoS One. 2024 May 6;19(5):e0302981. doi: 10.1371/journal.pone.0302981 (PMC11073737; doi:10.1371/journal.pone.0302981)
Supplement: S1 Table — Ensemble models are comprised of multiple single-algorithm models (GAM, GBM, and RF) with true skill statistics (TSS) scores >70%. Ensemble algorithms (EM.algo) include committee averaging (Ca) and weighted mean (wmean; [82]), evaluation metrics (Eval.met) include relative operating characteristic (ROC) and TSS, and reported values include ensemble model sensitivity (sens, i.e., true positive rate), specificity (spec, i.e., true negative rate), and calibration (cal). (PDF) [file pone.0302981.s005.pdf]

Table S1. Ensemble species distribution models for *Thamnophis* gartersnakes in Arizona, 1980–2021. Ensemble models are comprised of multiple single-algorithm models (GAM, GBM, and RF) with true skill statistics (TSS) scores >70%. Ensemble algorithms (*EM.algo*) include committee averaging (*Ca*) and weighted mean (*wmean*; [78]), evaluation metrics (*Eval.met*) include relative operating characteristic (*ROC*) and *TSS*, and reported values include ensemble model sensitivity (*sens*, i.e., true positive rate), specificity (*spec*, i.e., true negative rate), and calibration (*cal*).

**Species**	**EM.algo**	**Eval.met**	**sens**	**spec**	**cal**
T. cyrtopsis	Ca	ROC	99.206	93.120	0.923
		TSS	99.206	93.120	0.986
	Wmean	ROC	98.413	90.481	0.889
		TSS	98.413	90.514	0.977
T. elegans	Ca	ROC	99.123	94.821	0.939
		TSS	99.123	94.821	0.991
	Wmean	ROC	97.368	93.785	0.912
		TSS	97.368	94.019	0.989
T. eques	Ca	ROC	100.000	99.900	0.999
		TSS	100.000	99.900	1.000
	Wmean	ROC	100.000	99.766	0.998
		TSS	100.000	99.766	0.999
T. marcianus	Ca	ROC	100.000	93.612	0.936
		TSS	100.000	93.612	0.990
	Wmean	ROC	98.276	93.278	0.916
		TSS	98.276	93.445	0.987
T. rufipunctatus	Ca	ROC	100.000	99.833	0.998
		TSS	100.000	99.833	1.000
	Wmean	ROC	100.000	99.799	0.998
		TSS	100.000	99.799	1.000
