# Supplementary material for: Modeling a hot, dry future: Substantial range reductions in suitable environment projected under climate change for a semiarid riparian predator guild
Source: PLoS One. 2024 May 6;19(5):e0302981. doi: 10.1371/journal.pone.0302981 (PMC11073737; doi:10.1371/journal.pone.0302981)
Supplement: Attachment — Submitted filename: PONE-D-23-43716.pdf [file pone.0302981.s007.pdf]

PLOS ONE

Modeling a hot, dry future: substantial range reductions in suitable environment projected under climate change for a semiarid riparian predator guild

--Manuscript Draft--

**Manuscript Number:**	PONE-D-23-43716
**Article Type:**	Research Article
**Full Title:**	Modeling a hot, dry future: substantial range reductions in suitable environment projected under climate change for a semiarid riparian predator guild
**Short Title:**	Suitable environment declines with climate change
**Corresponding Author:**	Brian R Blais<br>The University of Arizona College of Agriculture and Life Sciences<br>Tucson, AZ UNITED STATES
**Keywords:**	species distribution model; ecological niche model; aridland; bioclimatic; gartersnakes; endangered species
**Abstract:**	An understanding of species-environmental relationships is invaluable for effective conservation and management under anthropogenic climate change. Warming and drying conditions threaten aridland riparian habitats—biodiversity hotspots. Species distribution models (SDMs) assess the relationships between species occurrences and environmental conditions, which can then be modeled to project potential suitable environments through space and time. We generated 260 ensemble SDMs for five species of Thamnophis gartersnakes—an important riparian predator guild—in a semiarid and biogeographically diverse region (Arizona, United States) to 1) understand spatial shifts in recent history; 2) assess a suite of environmental predictors influencing present distributions; and 3) model and project changes to suitable environment under 12 future climate scenarios per species, including the most and least optimistic greenhouse gas emission pathways, through 2100. We detected a northerly shift across time for historical Thamnophis occurrences (n = 955) in Arizona. Seven environmental variables best represented present distributions (n = 347); overwinter temperature and seasonal precipitation were among the most important variables. On average, the future ranges of suitable environment for Thamnophis are projected to decrease (mean = -37.1 ±SE 15.5%). Species already threatened with extinction or those with warm trailing-edge populations likely face the greatest loss of suitable environment. Climate change scenarios suggest an upward advance of suitable environment around montane areas for some low to mid-elevation species, which may create pressures to ascend. By bounding the climate pathway extremes to reduce SDM uncertainties, we provide valuable information to help conservation practitioners mitigate climate-induced threats to species. Modeled areas of sustained or gained suitable environment can be compared to present distributions, including applicable critical habitat designations, to identify potential safe zones to prioritize conservation refuges. Implementing informed conservation actions is paramount for sustaining aridland riparian biodiversity as the climate warms and dries.
**Order of Authors:**	Brian R Blais, PhD, MS
	John Koprowski
**Additional Information:**	
**Question**	**Response**
**Financial Disclosure**<br><br>Enter a financial disclosure statement that describes the sources of funding for the work included in this submission. Review the submission guidelines for detailed requirements. View published research articles from *PLOS ONE* for specific	The author(s) received no specific funding for this work.

examples.<br><br>This statement is required for submission and **will appear in the published article** if the submission is accepted. Please make sure it is accurate.<br><br>**Funded studies**<br>Enter a statement with the following details:<br>• Initials of the authors who received each award<br>• Grant numbers awarded to each author<br>• The full name of each funder<br>• URL of each funder website<br>• Did the sponsors or funders play any role in the study design, data collection and analysis, decision to publish, or preparation of the manuscript?<br><br>Did you receive funding for this work?	
**Competing Interests**<br><br>Use the instructions below to enter a competing interest statement for this submission. On behalf of all authors, disclose any competing interests that could be perceived to bias this work—acknowledging all financial support and any other relevant financial or non-financial competing interests.<br><br>This statement is required for submission and **will appear in the published article** if the submission is accepted. Please make sure it is accurate and that any funding sources listed in your Funding Information later in the submission form are also declared in your Financial Disclosure statement.<br><br>View published research articles from *PLOS ONE* for specific examples.	The authors have declared that no competing interests exist.

**NO authors have competing interests**<br><br>Enter: *The authors have declared that no competing interests exist.*<br><br>**Authors with competing interests**<br><br>Enter competing interest details beginning with this statement:<br><br>*I have read the journal's policy and the authors of this manuscript have the following competing interests: [insert competing interests here]*<br><br>* typeset	
**Ethics Statement**<br><br>Enter an ethics statement for this submission. This statement is required if the study involved:<br><br>• Human participants<br>• Human specimens or tissue<br>• Vertebrate animals or cephalopods<br>• Vertebrate embryos or tissues<br>• Field research<br><br>Write "N/A" if the submission does not require an ethics statement.<br><br>General guidance is provided below. Consult the submission guidelines for detailed instructions. **Make sure that all information entered here is included in the Methods section of the manuscript.**	N/A

**Format for specific study types**<br><br>**Human Subject Research (involving human participants and/or tissue)**<br>• Give the name of the institutional review board or ethics committee that approved the study<br>• Include the approval number and/or a statement indicating approval of this research<br>• Indicate the form of consent obtained (written/oral) or the reason that consent was not obtained (e.g. the data were analyzed anonymously)<br><br>**Animal Research (involving vertebrate animals, embryos or tissues)**<br>• Provide the name of the Institutional Animal Care and Use Committee (IACUC) or other relevant ethics board that reviewed the study protocol, and indicate whether they approved this research or granted a formal waiver of ethical approval<br>• Include an approval number if one was obtained<br>• If the study involved *non-human primates*, add *additional details* about animal welfare and steps taken to ameliorate suffering<br>• If anesthesia, euthanasia, or any kind of animal sacrifice is part of the study, include briefly which substances and/or methods were applied<br><br>**Field Research**<br><br>Include the following details if this study involves the collection of plant, animal, or other materials from a natural setting:<br>• Field permit number<br>• Name of the institution or relevant body that granted permission	
**Data Availability**<br><br>Authors are required to make all data underlying the findings described fully available, without restriction, and from the time of publication. PLOS allows rare exceptions to address legal and ethical concerns. See the PLOS Data Policy and FAQ for detailed information.	No - some restrictions will apply

Powered by Editorial Manager® and ProduXion Manager® from Aries Systems Corporation

A Data Availability Statement describing where the data can be found is required at submission. Your answers to this question constitute the Data Availability Statement and **will be published in the article**, if accepted.<br><br>**Important:** Stating 'data available on request from the author' is not sufficient. If your data are only available upon request, select 'No' for the first question and explain your exceptional situation in the text box.<br><br>Do the authors confirm that all data underlying the findings described in their manuscript are fully available without restriction?	
**Describe where the data may be found in full sentences. If you are copying our sample text, replace any instances of XXX with the appropriate details.**<br><br>• If the data are **held or will be held in a public repository**, include URLs, accession numbers or DOIs. If this information will only be available after acceptance, indicate this by ticking the box below. For example: *All XXX files are available from the XXX database (accession number(s) XXX, XXX.)*.<br>• If the data are all contained **within the manuscript and/or Supporting Information files**, enter the following: *All relevant data are within the manuscript and its Supporting Information files.*<br>• If neither of these applies but you are able to provide **details of access elsewhere**, with or without limitations, please do so. For example:<br><br>*Data cannot be shared publicly because of [XXX]. Data are available from the XXX Institutional Data Access / Ethics Committee (contact via XXX) for researchers who meet the criteria for access to confidential data.*<br><br>*The data underlying the results presented in the study are available from (include the name of the third party*	Data are provided within the manuscript and it's Supporting Information files. Occurrence data are available within most database sites listed in the Methods section, except for species with designations under the Endangered Species Act. Because of the sensitive nature of those data, their occurrences cannot be shared publicly; data inquiries can be directed to Arizona Game and Fish Department, Heritage Data Management System (HDMS) Program Manager at 623 236-7618 or HDMS@azgfd.gov. Additional inquiries can be directed to the corresponding author.

and contact information or URL).<br>• This text is appropriate if the data are owned by a third party and authors do not have permission to share the data.<br><br>* typeset	
Additional data availability information:	

Powered by Editorial Manager® and ProduXion Manager® from Aries Systems Corporation

Manuscript
Click here to access/download;Manuscript;Thamnophis_SDM_manuscript.doc

Modeling a hot, dry future: substantial range reductions in suitable environment projected under climate change for a semiarid riparian predator guild

Short title: Suitable environment declines with climate change

Brian R. Blais[1]*, John L. Koprowski[1,#a]

[1] School of Natural Resources and the Environment, University of Arizona, Tucson, Arizona, United States of America,

[#a] Current Address: Haub School of Environment and Natural Resources, University of Wyoming, Laramie, Wyoming, United States of America

*Corresponding author

E-mail: bblais@arizona.edu (BRB)

Abstract

An understanding of species-environmental relationships is invaluable for effective conservation and management under anthropogenic climate change. Warming and drying conditions threaten aridland riparian habitats—biodiversity hotspots. Species distribution models (SDMs) ~~assess the relationships between species occurrences and environmental conditions, which~~ can ~~then be modeled to~~ project potential suitable environments through space and time. We generated 260 ensemble SDMs for five species of *Thamnophis* gartersnakes—an important riparian predator guild—in a semiarid and biogeographically diverse region (Arizona, United States) to 1) understand spatial shifts in recent history; 2) assess a suite of environmental predictors influencing present distributions; and 3) model and project changes to suitable environment under 12 future climate scenarios per species, including the most and least optimistic greenhouse gas emission pathways, through 2100. We detected a northerly shift across time for historical *Thamnophis* occurrences (n = 955) in Arizona. Seven environmental variables best represented present distributions (n = 347); overwinter temperature and seasonal precipitation were among the most important variables. On average, the future ranges of suitable environment for *Thamnophis* are projected to decrease (~~mean = -37.1 ±SE 15.5%~~). Species already threatened with extinction or those with warm trailing-edge populations likely face the greatest loss of suitable environment. Climate change scenarios suggest an upward advance of suitable environment around montane areas for some low to mid-elevation species, which may create pressures to ascend. By bounding the climate pathway extremes to reduce SDM uncertainties, we provide valuable information to help conservation practitioners mitigate climate-induced threats to species. Modeled areas of sustained or gained suitable environment can be compared to present distributions, including applicable critical habitat designations, to

identify potential safe zones to prioritize conservation refuges. Implementing informed conservation actions is paramount for sustaining aridland riparian biodiversity as the climate warms and dries.

Introduction

An understanding of species-environmental relationships is invaluable to project potential outcomes under future scenarios [1–3]. Such insights are critical for managing biodiversity conservation during anthropogenic climate change [4,5]. Globally, increases in aridity and temperature will likely place added pressure on water availability and demand [6]. Aridland ecosystems, such as the southwestern United States, are predicted to become warmer and drier [7–10]. This can affect ecosystems through increased frequencies of major disturbances and extreme climatic events [11], especially in biodiverse riparian zones [12–14]. Slower, gradual changes to habitats are also occurring [15–17]. Ecosystem changes can adversely affect biota [18–23] and influence how species respond to challenges, and thereby are of key importance for the maintenance of biodiversity [24–29].

Analyzing and predicting species distributions based on environmental gradients is required to decipher patterns in ecological niches [30–32]. Species distribution models (SDMs; *syn*. climate envelope models, ecological/environmental niche models, habitat suitability models; [33]) assess the relationships between species occurrence and environmental conditions (i.e., realized niche), which can then be modeled to infer and project potential suitable environments through space and time [3,34,35]. Although numerous tools, steps, and pathways exist to facilitate SDMs for a given set of parameters and objectives [36–38], novel advances in technologies help enable conservation practitioners to develop customized applications in ways that can be evaluated across multiple scenarios [39–43]. These tools are essential to guide effective conservation planning in light of climate change and extinction risks faced by vulnerable species [37,44,45].

Gartersnakes (*Thamnophis* spp.) usually have life histories connected to surface water and are often considered riparian-dependent [46]. *Thamnophis* are distributed throughout the Americas, comprise an intermediate predatory guild of generalists and specialists, and are important in energy transfer between aquatic and terrestrial habitats that help uphold ecosystem integrity [47,48]. *Thamnophis* can also be sensitive to disturbances and changes to habitat and environment [49–53]. Given their trophic position, occupancy of important areas of biodiversity (e.g., riparian zones), and environmental susceptibility, *Thamnophis* make ideal model species to assess bioclimatic influences on contemporary distributions and project how climate change patterns may impact future distributions.

Herein, we were interested in how contemporary and future climatic trends may impact environmentally sensitive species in a warming and drying semiarid climate (Arizona, United States). We used *Thamnophis* as a model system to address the following objectives: 1) examine spatial patterns in recent historical and contemporary occurrences; 2) assess explanatory relationships of bioclimatic variables on present distributions via ensemble species distribution models; and 3) model and map projected changes of suitable environmental range under multiple future climate scenarios. Given the contemporary trends and likelihood of increased heat and drought in the southwestern U.S. region, we hypothesized reductions of suitable environmental range for gartersnakes across time. We also expected to see reflections of upward advancement—in both elevation and latitude—as observed with numerous species impacted by climate change [54–57]. Coupled with ongoing challenges faced by *Thamnophis* in aridland climates [49,50,52,58], contraction in suitable environment will likely exacerbate existing risks to population demographics [59]. Less is known about the environmental influences on gartersnakes in aridlands; our assessment of suitable environment can help guide multi-species

conservation management decisions, especially for species already under threat of extinction. More broadly, gartersnakes are important indicator species and their relationships and responses to environmental change can serve as an early warning system for how other riparian-linked taxa with similar life histories may respond to climate change in aridland regions.

Materials and methods

Study area and system

In the southwestern United States, the semiarid state of Arizona is biogeographically diverse and experiences a wide range of seasonally variable climatic gradients, including a bimodal annual precipitation pattern with periods of drought fluctuated between rainy winters and summers [60–62]. The region is predicted to become hotter and drier, with less frequent but more intense precipitation trends likely to further strain natural resources [7,10,63–65]. These characteristics make Arizona an ideal study area to model present and future impacts of environmental change on representative taxa. State-extent assessments may also facilitate collaboration and extrapolation of conservation management applications among both localized and broader regional levels.

Five species of *Thamnophis* occur in Arizona; three are considered common (*T. cyrtopsis* = black-necked gartersnake; *T. elegans* = terrestrial gartersnake; and *T. marcianus* = checkered gartersnakes) and two rare species (*T. eques* = Mexican gartersnake; *T. rufipunctatus* = narrow-headed gartersnake) that have threatened status at state and federal levels [52,58,59]. Their primarily allopatric distributions range from low desert drainage networks to subalpine conifer forest biomes, though some interspecific syntopy occurs [46,52,66].

Occurrence and climatic data curation

We obtained georeferenced occurrence data for *Thamnophis* in Arizona from museum records (e.g., Vertnet), the global biodiversity information facility (GBIF, [67]), iNaturalist, field projects, and technical reports; records spanned from the 1800s through 2021. We manually inspected records with geocoordinate uncertainty ≥0.5 km—a conservative proxy of home range size for the focal taxa (ca. ≤10 ha [46,52,66])—and retained records where metadata descriptions were sufficiently detailed to localities. Otherwise, we omitted imprecise records (>0.5 km) with vague descriptions, those with obscured/private locality masks, and platform cross-duplicates. We also omitted highly suspect localities (see [52]).

To assess present-time environmental influences on *Thamnophis* distributions, we obtained elevation and (19) bioclimatic predictor variables at 0.5′ (~1 km^2) spatial resolution from the WorldClim Version 2 dataset [68]. We focused on bioclimatic variables and elevation—which is climatically linked [60]—for their explanatory power in SDM predictions and likelihood to drive changes to species distributions [34,69]. We used ArcMap v.10.8 (ESRI, Redlands, CA) to crop predictors to the spatial extent of the Arizona state boundary.

For future projections, we again used WorldClim v.2 to obtain bioclimatic variables that have been downscaled and projected for two future periods, three global climate models (GCMs), and two shared socio-economic pathways (SSPs)—derived from the Coupled Model Intercomparison Project 6th assessment (CMIP6) data [6]. We selected HadGEM3-GC31-LL (“HAD”), MPI-ESM1-2-LR (“MPI”), and MRI-ESM2-0 (“MRI”); these GCMs have performed well for SDMs in North America, including representation of the North American Monsoon that drives summer climate in our study region [69–73]. For each GCM, we assessed two SSPs: low reflected various emission projections (SSP126, i.e., progressive action towards sustainable

development to limit ca. +1.8 °C by 2100) and high emission projections (SSP585, ca. +4.4 °C by 2100, i.e., *status quo* trajectory of continued societal and energy practices; [6]). These SSPs reflect the most optimistic and pessimistic pathways, respectively [74], and have been used for modeling southwestern herpetofauna [69,73]. For each SSP per GCM, we assessed near-future (2041–2060, hereafter "2050", i.e., median year) and distant future (2081–2100, hereafter "2090") time periods. By assessing various combinations of GCMs, SSPs, and time periods (i.e., "scenarios"), including best-case/worst-case pathways, we better represent uncertainties in projections that should outweigh loss of modeled precision obtained from a single model scenario [73,75].

Historic spatial trends

To estimate *Thamnophis* range shifts in recent history, we partitioned the occurrences dataset into three time periods. Records prior to 1940 reflect the pioneering days of herpetological exploration [76], 1940–1979 characterize increased efforts to supply herpetological collections [77], and 1980–2021 represent contemporary ("present") records that we used to model environmental relationships. We used the Directional Distribution tool in ArcMap to calculate the central tendency, dispersion, and rotational trends per historical period, then calculated distance and directional change among mean centers per period. We also estimated the relative percent change in standard deviational ellipse area, i.e., expansion or contraction in occurrence sampling patterns, by dividing the standard distance ellipse area (X × Y) among time periods. We combined all *Thamnophis* species for these tests to increase sample size and infer broad spatial change patterns reflected by our five species.

Species distribution modeling and evaluation

We used the R package biomod2 version 4.2-2 [39,78] to generate SDMs separately per species. We built "present" scenario SDMs with only 1980–2021 records to avoid erroneously assessing since-altered habitat or local extirpations [52]. We used the spThin package [79] to delimit occurrences to 1/km^2 separately for each species [80]; this scale corresponded to that of the environmental layers. We created 1000 randomly distributed pseudo-absences, i.e., background points representing available environment [81,82], per modeling run. Thickening the number of background points greater than presence points has performed well in SDM [83], especially for taxa with limited sample size [84].

We ran preliminary modeling pipelines (see below) for each species separately with all bioclimatic predictors except those comprising precipitation-temperature interactions; these variables are problematic with discontinuities [85], especially in regions with bimodal precipitation distributions like Arizona. We further reduced the number of predictors in three ways based on collinearity (omittance $|r| > 0.7$; [86]), ranked variable importance scores from biomod2 to break ties among correlated pairs [87], and *a priori* knowledge from the literature and expertise of the focal system [88]. This process yielded three temperature-related predictors (Bio4–5,11), three precipitation factors (Bio15–17), and elevation for downstream analyses. Several of these predictors have worked well for snake SDMs in similar latitudes or regions, including congenerics and conspecifics [49,89–93].

We used a multi-algorithm ensemble modeling approach [40] that included generalized additive models (GAM), generalized boosted models (GBM, i.e., boosted regression trees), and random forest (RF) algorithms [39,78]. We chose these algorithms based upon repeated high performance in modeling trials among available algorithms in biomod2. For GAMs, we used

restricted maximum likelihood penalty without interaction via package mgcv [94] to minimize overfitting [73]; 1000 trees for RF; and default settings for GBM. Throughout, we ran three repetitions each for pseudo-absences permutations, single and ensemble model calibration/validations, and variable importance estimations. We split datasets for training (80%) and testing (20%) per run and evaluated models via relative operating characteristic (ROC) and true skill statistic (TSS) metrics [78,95]. We built ensembles from single algorithm models performing >70% percentile TSS threshold via committee averaging (ca) and proportionally-weighted means (wmean) ensemble algorithms [78]; we again used ROC and TSS to evaluate resulting ensembles.

For future projections, we used BIOMOD_EnsembleForecasting function in biomod2 to project ensemble models onto new environments per each GCM × period × SSP scenario. We did this for each species separately. The calculated output was used to visualize distribution of suitable environment as well as predict changes in suitable range size between present and each future scenario. We used Kruskal-Wallis tests to assess model evaluation scores between ensemble algorithms, GCMs, and metrics, respectively. Throughout, we performed all analyses in ArcMap or program R v.4.1.1 [96].

Results

Historical shifts

After data refinement, we retained n = 955 *Thamnophis* records for historical analyses (Table 1). Discernable temporal trends across historical records exist for *Thamnophis*. Patterns in central tendency reveal a northwestern progression that has become more directional relative the

central highlands—a diverse ecotone between desert ecoregions and the Mogollon Rim, i.e., the southern extent of the Colorado Plateau (Fig S1; [60,97]). Mean centers have shifted northwesterly (285.9°) by approximately 5.4 km between pre-1940 to the 1940–1979 periods, and 68.1 km (333.5° NW) to the 1980–present distribution. The area of concentration has contracted across time. Standard ellipse areas reduced by 42.2% between pre-1940 and 1940–1979 periods and another 18.9% from the 1940–1979 period to present. That is, occurrence sampling has become more spatially concentrated. These results partially support our hypothesis of a northerly latitudinal trend across time. We acknowledge that collection biases, e.g., sampling bias approximate roads and easily accessible locations [98], may exist and that our results may differ from true distributions of *Thamnophis* in our study area. We believe, however, that our exhaustive record acquisitions from multiple sources and careful inspection filtering therein represents the best available information to diagnose recent historic patterns.

Table 1. **Georeferenced occurrences for the five *Thamnophis* gartersnake species recorded in Arizona through 31 December 2021.** Data sources include museum and biodiversity repositories, iNaturalist, agency reports, and records collected by authors. Data was manually inspected for duplicates, erroneous records, and confirmation of location accuracy for records with geocoordinate error ≥0.5 km.

**Gartersnake Species in Arizona**	**Pre-1940**	**1940-1979**	**1980-2019‡**
Thamnophis cyrtopsis – black-necked gartersnakes	17	54	180 (127)
Thamnophis elegans – terrestrial gartersnake	13	37	170 (114)
Thamnophis eques – Mexican gartersnake^{T}	5	11	26 (20)
Thamnophis marcianus – checkered gartersnake	3	37	83 (58)

Thamnophis rufipunctatus – narrow-headed gartersnake[T]	2	21	476 (28)

[‡] numbers in parentheses are records thinned to 1 per km^2; [T] = "threatened" status at Arizona and Federal levels.

Environmental drivers of *Thamnophis* distribution

After spatially thinning occurrences, n = 347 *Thamnophis* records remained available for present SDM analyses (Fig 1, Table 1). From those data, we created 20 ensemble models to derive environmental suitability. We detected no differences among ensemble algorithm scores (ca vs wmean) for model sensitivity (H = 3.2, df = 1, $p = 0.075$), specificity (H = 1.1, df = 1, $p = 0.289$), and calibration (H = 0.5, df = 1, $p = 0.471$; Table S1). Likewise, evaluation metrics (ROC vs TSS) were equivalent in sensitivity (H = 0.0, df = 1, $p = 1.000$) and specificity (H = 0.1, df = 1, $p = 0.910$); ROC trended to have higher calibration scores although both metrics scored >0.95 on average (H = 3.7, df = 1, $p = 0.053$; Table S1). In summary, all ensemble models yielded satisfactory performance scores.

Fig 1. Distribution of *Thamnophis* gartersnake occurrences in Arizona, 1980–2021.

Predictor variable importance by species were equivalent (H = 4.6, df = 4, $p = 0.334$; Fig 2). The most influential predictors overall, however, were Bio11 (mean temperature of coldest quarter, i.e., overwinter dormancy period) and Bio16 (precipitation during wettest quarter, i.e., summer monsoonal period), and Bio15 (precipitation seasonality [coefficient of variation]; Table 2). Elevation and Bio5 (maximum temperature of warmest month) were the least influential overall, albeit the latter was important to *T. elegans*. However, importance among all predictors were equivalent (H = 5.1, df = 6, $p = 0.528$).

Table 2. Environmental variables used in ensemble species distribution modeling for *Thamnophis* gartersnakes in Arizona, 1980–2021. *Mean rank* is the mean variable importance score rank (out of 7) among five species, *Thamnophis cyrtopsis*, *T. elegans*, *T. eques*, *T. marcianus*, *T. rufipunctatus*; lower mean rank equals higher importance.

Variable[a]	Description	Mean rank
Bio4	temperature seasonality (SD × 100)	5
Bio5	max temperature of warmest month	5.2
Bio11	mean temperature of coldest quarter	2.6
Bio15	precipitation seasonality (coefficient of variation)	3.6
Bio16	precipitation of wettest quarter	3
Bio17	precipitation of driest quarter	4
Elev	elevation (meters above sea level)	4.6

[a] source: WorldClim v. 2 database [68]

Fig 2. **Predictor importance in species distribution models of environmental suitability for *Thamnophis* gartersnakes in Arizona, 1980–2021.** Variable importance scores range between 0 and 1 per species; column height reflects cumulative influence. Bioclimatic *predictor*s are described in Table 2.

Precipitation variables were most important for *T. eques*, *T. marcianus*, and *T. rufipunctatus* whereas temperature-linked predictors best explained *T. cyrtopsis* and *T. elegans* distribution. Individually, *T. cyrtopsis* were most influenced by Bio4, Bio16, and Bio15 (~~mean ± standard deviation (SD) variable importance =~~ 0.162 ±0.082); these related to environmental seasonality and summer precipitation. *Thamnophis elegans* were best predicted by the temperature variables: Bio4–5,11 (~~mean ±SD variable importance =~~ 0.243 ±0.136). *Thamnophis eques* were best predicted by Bio17, Bio15, and Bio11 (~~mean ±SD variable importance =~~ 0.326 ±0.157); these relate to overwinter temperature and precipitation during the active season.

Thamnophis marcianus were influenced by Bio15, Bio16, and Bio11 (~~mean ±SD variable importance =~~ 0.234 ±0.170); these relate to precipitation in the wettest parts of summer and overall seasonality as well as mean overwinter temperature. *Thamnophis rufipunctatus* were most influenced by Bio16, Bio11, and Bio17 (~~mean ±SD variable importance =~~ 0.318 ±0.219); these relate to mean overwinter temperature and precipitation throughout the active season.

Future projections of environmental range

We generated another 240 ensemble models encompassing all future projection scenarios (Fig S2). Both ensemble algorithms (ca and wmean) were congruent in projected range sizes (e.g., stable, gains, losses, unsuitable area) except for present range size (H = 11.7, df = 1, $p < 0.001$)—likely influenced by per pixel area of unsuitable environment (H = 4.3, df = 1, $p = 0.038$). That is, the majority of the area in present sampling frames are absent of occurrences and thus considered unsuitable. More importantly, algorithms were not different in predicted gains and losses (all $p > 0.05$). Global climate models were congruent in range projections (all $p > 0.05$) except percent gain (H = 9.0, df = 2, $p = 0.011$), though we note a related trend in per-pixel gain (H = 5.0, df = 2, $p = 0.082$). For SSPs by period scenarios, many similarities exist ($0.182 \leq p \leq 1.0$) except for percent loss (H = 11.0, df = 3, $p = 0.012$) and percent species range change (H = 7.8, df = 3, $p = 0.049$). Overall, GCMs were comparable; HAD exhibited the greatest variation in percent range change per period scenario whereas MRI appeared to be most consistent (Fig S2).

Because wmean and ca algorithms yielded equivalent results but weighted mean ensembles are most often used due to their performance [40], we use wmean hereafter to summarize our findings related to changes in projected environmental suitability (Fig S3), percent range change (Fig 3), and types of range change by area (Fig 4). All species are predicted

to have net reductions in suitable environmental range (~~mean ± standard error (SE) =~~ -37.1 ±15.5%), though *T. marcianus* is predicted to gain suitable environment (~~mean ± standard error (SE) =~~ 28.1 ±25.0%; Fig 3). The two species occurring at higher elevations are predicted to lose considerable suitable environment among all SSP scenarios (*T. elegans* ~~mean =~~ -84.5 ±~~SE~~ 2.8%; *T. rufipunctatus* ~~mean =~~ -84.5 ± ~~SE~~ 7.0%; Fig 3). *Thamnophis eques* is also likely to experience net losses of suitable environment under all SSP scenarios (-34.9 ±~~SE~~ 16.2%), except into 2090 under SSP126 (15.0 ±11.5%; Fig 3). Although there was a net negative range change for *T. cyrtopsis* (mean = -10.3 ±26.6%; Fig 3), GCMs for this species exhibited the most variation, including estimated positive (HAD) and negative (HAD, MPI, MRI) range changes. Variation exhibited by species and scenario are tabulated in Dataset S1.

Fig 3. **Estimated future changes in suitable environmental range for *Thamnophis* gartersnakes in Arizona.** Data are derived from the means of weighted-mean ensemble models of three global climate models (GCM), two shared socio-economic pathways (SSP), and two future time period scenarios per species. Bar plots depict mean change plus standard error among GCMs from baseline (0, i.e., present distributional range, 1980–2021). Positive values reflect net increases whereas negative values estimate net reductions.

Fig 4a–t. **Projected change in range sizes for *Thamnophis* gartersnakes in Arizona.** Data are modeled from weighted-means ensemble models generated from the MRI-ESM2-0 global climate model. Data columns represent near future ("2050", i.e., 2041–2060 median) and distant future ("2090", i.e., 2081–2100 median) projections under shared socio-economic pathways (SSPs) that represent most optimistic (SSP126) and pessimistic (SSP585, i.e., *status quo*) emissions-limiting models. Individual map panels for species (a–t) are shown in Fig S4.

Spatially, favorable and unfavorable area projections were evident under future scenarios, i.e., suitable environment retained or gained (Dataset S1, Fig 4). The relatively warmer and drier southern part of Arizona may become more environmentally unfavorable across time, although this phenomena may be topographically dampened by disjunct, insular mountains—the Madrean Sky Islands [99]. Predicted stability and change in suitable environment appear to be elevationally linked, in which lower, montane foothill areas around the Sky Island region reflect losses and areas ascending into higher elevations are more likely to remain stable (Figs 4,S3). Gains also appear to increase latitudinally, especially around the transition zones of the central highlands and Mogollon Rim region (Figs 4,S3).

Predicted declines in suitable range were mostly congruent among GCMs for both threatened species. Projections that follow the most optimistic emissions pathway (SSP126) appear less unfavorable to *T. eques* (-14.7 ±SE 10.7%) than under the SSP585 *status quo* pathway (-54.0 ±SE 21.7%; Fig 3, Dataset S1). Southern populations may be most affected, with large swaths anticipated to become unsuitable (Fig 4i–l). Northern populations of *T. eques,* however, may have areas of sustained or gained suitable environment under progressive climate actions (SSP126; Figs 4i–l, S3k–o). *Thamnophis rufipunctatus* may face even greater climatic challenges across time. Suitable environmental refuges are predicted to become highly reduced and localized (Figs 4q–t, S3u–y); only an average of 47.3 pixels (i.e., approx. 47 km^2 area) of suitable environment is projected to be gained (Dataset S1). By the late 21st century, all GCMs predicted a complete loss (-100%) of suitable environment for *T. rufipunctatus* under the nonaction pathway, whereas prompt, sustainable emissions reductions could still yield high range reductions (mean = -74.3 ±SE 11.5%).

Discussion

Through ensemble species distribution modeling, we provide likelihoods of influential environmental predictors on *Thamnophis* gartersnakes—a model system guild important for energy transfer between aquatic and terrestrial ecosystems. We also model predicted changes to suitable environmental ranges under various climate change scenarios. Our results show that *Thamnophis* species in semiarid regions such as Arizona are predicted to experience changes—mostly reductions ~~(all species: mean = -37.1 ±SE 15.5%)~~—to their suitable environment ranges in the future.

Modeling considerations and estimating distributional trends

Species distribution models can provide valuable information to researchers, natural resource managers, and decision makers, and cautious methodological planning is important during model development stages [100]. For example, we addressed spatial extent considerations [35], ecologically relevant occurrence thinning [79] and pseudo absence parametrization [82,84], predictor selection [35,86,100,101], training and testing data splitting and calibration [95], and ensemble modeling and evaluation [40,75]. Part of our predictor selection process included using *a priori* information from expertise and relevant literature [88]. Minimum winter temperature, maximum summer temperature, and precipitation, for instance, were found to be important environmental predictors of *Thamnophis* in other regional studies [49,90]. We found overwinter temperature, summer precipitation, and precipitation seasonality ~~(i.e., Bio11, Bio16, and Bio15, respectively)~~ among the top predictors that explained *Thamnophis* occurrence in our study. Our high performance evaluation scores suggest that our selected variables and ensemble modeling process adequately reflect aridland *Thamnophis*-environmental relationships.

Predicting suitable niche space under future projections may be best achieved by modeling under various climate-model scenarios, i.e., GCM × SSP × time frames [75,101]. We selected three GCMs that have performed well climatically (e.g., North American Monsoon) and geographically for our study region. All projected SDMs contain a certain degree of uncertainty and variation in predictions [74,101]. By evaluating multiple scenarios that bound climate change pathway extremes, such as the least and most optimistic SSPs, we reduced uncertainties that would be missed by single scenario models [69,73]. Providing central tendencies and dispersion among a range of outcomes should help guide future decision making. ~~We believe our modeling steps and results should thus convey a breadth of possibilities for potential niches available to *Thamnophis* and other similarly distributed taxa across space and time through the 21st century.~~

Extrapolating SDMs (i.e., transferability) to novel spatial and temporal environments offers additional opportunities to modelers [102,103], albeit caution and deliberation exists in transferring models to new areas [38]. Modeling methods are often refined and adjusted to original sampling parameters, different species and their traits may require different approaches, and new areas may not spatially or temporally exhibit the same abiotic and biotic attributes of the originally assessed space [102,104–106]. We summarize the relative extrapolation potential of our *Thamnophis* SDMs as follows: our models and results are likely to be most informative and applicable where focal taxa have contiguous or proximal neighboring populations (e.g., California, Utah, New Mexico, and Sonora Mexico in this study). The limited distribution of *T. rufipunctatus*, for example, extends into west-central New Mexico, where occupied habitat resembles those in Arizona [107]. We expect that the design and explanatory power of our Arizona-based models would perform comparably among the New Mexican range of *T.*

rufipunctatus. Conversely, *T. elegans* is a widely distributed species that ranges into southwestern Canadian latitudes, where habitats and bioclimatic patterns undoubtedly vary from those in Arizona [46,92,108]. That is, our *T. elegans* models could have better transferability to neighboring Utah populations than to those in Alberta. We recommend flexible model adjustment and testing as geographic × environmental distances increase and thus the potential influence of predictors can also change.

Projected changes among future scenarios

Much of the semiarid southwestern United States are trending towards hotter and drier conditions [7]. Precipitation events are likely to become more intense yet infrequent [63,64]. Even under optimistic emission pathways, the southwestern region is projected to face unprecedented drought towards the 22nd century [10]. Primary productivity of functional plant groups will likely be affected [9,65,104], with subsequent cascading effects across trophic dynamics [8,29]. In aridland systems, much biodiversity is concentrated around perennially or seasonally available surface water, such as riparian zones [109,110]. Anthropogenic expansion and water resource demands are predicted to rise across time [5], which will further exacerbate water availability [111,112]. Alteration, reduction, or depletion of aquatic networks can drastically and negatively change riparian habitat and stress biota that rely upon it [16,103,113], including *Thamnophis* [90].

Because *Thamnophis* species in our study area have varying distributions, niches, and status [52], we break down prospective summaries for each taxon under future climate scenarios. We find it interesting that *T. marcianus* is predicted to gain suitable environment, whereas all other congenerics face reduced suitability. Of assessed taxa, *T. marcianus* is the only species that is 1) common, 2) a dietary generalist), and 3) predominantly occupies various riparian wetland

systems at lower elevations (e.g., desertscrub and arid grasslands) in our study area [114]. The other four species are either rare or usually associated with habitats above desert valley floors. Our *T. maricianus* models identified a lesser degree of sensitivity to both dry season precipitation (late spring to early summer prior to monsoonal rains) and overwinter temperature. Overwinter temperature was strongly associated with taxa facing the greatest predicted losses of suitable environment, especially at higher elevations. Precipitation seasonality was among the most influential predictors for *T. marcianus*, that suggests this taxon may exhibit plasticity in adjusting for temporal variability in rainfall. Although we did not assess intrinsic traits in our analyses, our findings corroborate the adaptive variation in this species [115,116]. Given their adaptive potential, current population trends [52], and future projections from this study, *T. marcianus* may be more flexible to changing conditions than its aridland congenerics.

Precipitation during the driest season was among the most important factors for two threatened species (*T. eques* and *T. rufipunctatus*) but not for other congenerics. Overwinter temperature was also notably important; increased fluctuations in these seasonal factors may negatively affect these taxa. Seasonal relationships are not surprising given that *T. eques* is known to shift behaviors between active and inactive seasons [117], and often occupies aquatic drainages with flow regimes that are regulated by precipitation pulses [118]; and *T. rufipunctatus* life history phenology (e.g., parturition timing) are linked to summertime rain patterns and subsequent changes to aquatic habitat [46,119]. Other SDM studies for *T. eques* projected greater sensitivity to environmental extremes and, in support of our findings, that overwinter temperature is an important predictor [49,90]. In another study, summer precipitation was found to be important for both threatened gartersnakes and, in addition, maximum summer temperature for *T. eques* [90]. Our ensemble modeling differs from that study in that we found temperature to

be more important in winter than summer for both rare gartersnakes. All, however, estimated substantial declines in suitable areas, 7–62% for *T. eques* and 32–62% reductions for *T. rufipunctatus* by the end of the century [90]. Shifts in the timing, intensity, and duration of seasonal environmental patterns are likely to affect populations of these snakes.

Climatic shifts towards warmer summers and winters may negatively affect *T. elegans* the most. The top explanatory variables for this high-elevation generalist were all temperature related. Substantial projected reductions in suitable environment from our SDMs are comparable to congeneric *T. scalaris*, another common, high-elevation species in Mexico [49]. More broadly, the predictions for *T. elegans* align with findings occurring at a global scale in which range contractions and local extinctions often arise at the "warm edge" of a species range [54]; Arizona largely represents the warmer, southern extent of the widely distributed *T. elegans* [46,107,108].

Thamnophis cyrtopsis—a dietary generalist often occupying sloped, rocky riparian habitats [46,53,120,121]—presented the most variability in projected range change, resulting in either estimated percent increases or decreases pending the pathway scenario. Seasonality of predictors most influenced distribution for this species (Fig 2, Table 2). These results somewhat contrast to populations along central Mexico's Trans-Mexican Volcanic Belt, where *T. cyrtopsis* was found to be more sensitive to the extremes, including minimum winter temperature and warmest summer temperature [49]. All, however, predict a decline in suitability with increasing temperature. In our study, projected trends for *T. cyrtopsis* appear topographically linked, in that suitability favors an upward ascent (see Fig S3). This suggests that future refugia may occur in ascending habitats (e.g., Madrean evergreen woodlands), especially areas more buffered against extreme climatic shifts, and that lower foothill populations may face greater risks. Given a

sensitivity to seasonal environmental relationships [this study] and extreme disturbances [53], selective forces may pressure this taxon to ascend upward in elevation as is evident among other montane taxa [55,56,122–124]. There is only so far a taxon can advance, however, especially when reliant on riparian networks and available surface water [53].

Future directions

~~Changes in environment and climate are among the primary threats to sustaining biodiversity [125–127]. Species must either adapt to changes in place, disperse to areas with favorable environment, or go extinct [4,128,129]. Climate change has driven range shifts in numerous species, usually towards greater elevation or latitude, and has already caused substantial local extinctions around the world [54,57,129]~~. In semiarid regions such as the southwestern United States, significant shifts in upward movement and phenology of flora have already occurred [123,124]. For representative species that link transitions between terrestrial and aquatic habitats like *Thamnophis*, climatic stressors and challenges are likely to add pressure for adaptation. Common generalists like *T. cyrtopsis* and *T. marcianus* may be able to adjust to shifting and reestablished aquatic regimes (e.g., effluent-recharged Santa Cruz River; [130]), or those amenable to dispersion to more favorable areas may better cope with environmental changes in the long run. In the short term, however, extreme disturbances and rapid environmental changes can have swift, negative consequences even on generalist taxa [53,121]. As climate and subsequently habitats change, surveillance of demographic trends and environmental responses by common species are worth monitoring by natural resource managers tasked with anticipating and mitigating threats to biodiversity [5,50,131,132].

Modeling for rare taxa is understandably challenging. Species with narrow ranges may face substantial challenges if climate trends result in fragmentation or elimination of favorable

environment [73,80,103,133]. High-elevation, cold-adapted *Thamnophis* are more vulnerable to rising temperature [49]. Rising temperature is likely to adversely affect *T. rufipunctatus*—a specialist endemic to cold, clear-water riparian zones in the Gila River Watershed in Arizona and New Mexico [107]. Both threatened *T. eques* and *T. rufipunctatus* have had considerable population reductions since the late 20th century, with declines attributed to habitat loss or degradation, effects from invasive species, and disturbances such as wildfire [58,107,118,134]. Our findings of substantial reduction in suitable environment across all future scenarios are concerning and reflect additional stressors that populations may likely face. Further reductions of already small populations via environmental change heighten the risks related to demographic stochasticity—including but not limited to effects from genetic drift, inbreeding, and reduced heterozygosity [49,50,59].

A conservation-driven application of SDMs is to identify areas that comprise or may become "climate refugia" [135], i.e., species-specific safe places across time. Locating high-likelihood areas of environmental suitability under future change may be vital for sensitive taxa with limited distribution. Critical habitat assessments, for example, can be compared to SDM output. Our findings suggest that for *T. eques*, refugia may exist around their northerly, central-Arizona populations [118,136] whereas populations near the U.S. border with Mexico may face more disadvantageous climatic changes. This could further restrict connectivity potential with populations in Mexico [49,59]. For *T. rufipunctatus*, areas predicted to sustain suitable environment are likely to become highly confined, and the most optimal areas may be in central Arizona (e.g., Verde River Watershed) and extreme eastern Arizona (e.g., Upper Gila River Watershed; [107,137]). Connectivity of the latter with populations in western New Mexico (see [59]) may be imperative to mitigate heightened extinction risks for a taxon that may have little to

nowhere to go. Disjunct or questionable localities and shifting population trends represent gaps in assessing the distribution of this taxon in east-central Arizona [107].

Another SDM application geared for biodiversity or community-based strategies is "stacked" SDMs, where individual SDMs are overlayed to determine common areas of suitability [138]. Such an approach with *Thamnophis* should be carefully considered due to differences in distribution and niche rather than spatial overlap alone [52]. Applying a best strategy may be to focus on syntopic areas of mutual and stable suitable environment under future projections [139]. Approaches that consider regions with favorable microclimates better buffered against environmental stochasticity may also maximize conservation potential [140].

For proactive initiatives, conservation managers might best rigorously assess population demography, identify and manage priority habitat areas—especially where favorable under future projections, and implement practical conservation actions. Our modeling outcomes highlight potential reductions in suitability under future climate scenarios but also the importance of conserving connectivity that mitigates further loss of genetic diversity. With such, genetic rescue [141] via conservation translocations, including but not limited to *ex situ* or *in situ* stock populations, is worth strong consideration to help stave off climatic burdens of already threatened species [59,119,133,142]. We refer to Wood and others [59] for suggested population genomic augmentation strategies for threatened *T. eques* and *T. rufipunctatus* that may apply to our data here. Additional data and SDM testing refinement present opportunities to address spatiotemporal uncertainties in distribution and projections [98], especially as habitats and land-uses change, which is likely to benefit our understanding of species-environmental relationships under anthropogenic climate change.

Conclusion

Thamnophis are an important intermediate predator guild that link terrestrial and freshwater networks. Gartersnakes serve as excellent environmental bioindicators and may reflect climatic stressors induced on other biota with similar life histories reliant upon riparian systems. Our rigorous multi-scenario SDMs identified arrays of important predictors that drive the present distribution gartersnakes among Arizona's diverse landscapes and their outlook under future climate change. Our hypothesis of predicted declines in suitable environment are supported for three species, of mixed outcomes for one species, and seemingly rejected for *T. marcianus* that has a more favorable outlook. We also identify areas likely to become favorable or unfavorable for each species. This information can help guide conservation strategies now and into the future. Consideration of evidence-based conservation approaches are paramount to stave off extinction risks as the climate warms and dries.

Acknowledgments

We thank M. Bogan, M. Culver, I.M. Vela-Vargas, S. Wells, and C. Wissler for valuable feedback on an earlier version of this manuscript. M. Guéguen and R. Patin provided helpful assistance with biomod2 code formatting. We acknowledge the World Climate Research Programme, which, through its Working Group on Coupled Modelling, coordinated and promoted CMIP6.

References

1. Townsend CR, Hildrew AG. Species traits in relation to a habitat templet for river

systems. Freshw Biol. 1994;31: 265–275.

2. Gaston KJ. Global patterns in biodiversity. Nature. 2000;405: 220–227.

3. Pearson RG, Dawson TP, Berry PM, Harrison PA. SPECIES: A spatial evaluation of climate impact on the envelope of species. Ecol Modell. 2002;154: 289–300.

4. Pecl G, Araujo MB, Bell JD, Blanchard J, Bonebrake TC, Chen I-C, et al. Biodiversity redistribution under climate change: Impacts on ecosystems and human well-being. Science. 2017;355: eaai9214. doi:10.1126/science.aai9214

5. Cornford R, Spooner F, McRae L, Purvis A, Freeman R. Ongoing over-exploitation and delayed responses to environmental change highlight the urgency for action to promote vertebrate recoveries by 2030. Proc R Soc B. 2023;290: 20230464. doi:10.1098/rspb.2023.0464

6. IPCC. Summary for Policymakers. In: Pörtner H-O, Roberts D, Adams H, Adler C, Aldunce P, Ali E, et al., editors. Climate Change 2022: Impacts, Adaptation, and Vulnerability. Cambridge, UK and New York, NY, USA: Cambridge University Press; 2022. p. 33. doi:10.1017/9781009325844.001

7. Archer SR, Predick KI. Climate change and ecosystems of the Southwestern United States. Rangelands. 2008;30: 23–28. doi:10.2111/1551-501X(2008)30[23:CCAEOT]2.0.CO;2

8. Grimm NB, Chapin III FS, Bierwagen B, Gonzalez P, Groffman PM, Luo Y, et al. The impacts of climate change on ecosystem structure and function. Front Ecol Environ. 2013;11: 474–482. doi:10.1890/120282

9. Roby MC, Scott RL, Moore DJP. High vapor pressure deficit decreases the productivity and water use efficiency of rain-induced pulses in semiarid ecosystems. J Geophys Res Biogeosciences. 2020;125: e2020JG005665. doi:10.1029/2020JG005665

10. Cook BI, Ault TR, Smerdon JE. Unprecedented 21st century drought risk in the American Southwest and Central Plains. Sci Adv. 2015;1: e1400082. doi:10.1126/sciadv.1400082

11. Smith MD. An ecological perspective on extreme climatic events: a synthetic definition and framework to guide future research. J Ecol. 2011;99: 656–663. doi:10.1111/j.1365-2745.2011.01798.x

12. Stromberg JC, Lite SJ, Dixon MD. Effects of stream flow patterns on riparian vegetation of a semiarid river: implications for a changing climate. River Res Appl. 2010;26: 712–729. doi:10.1002/rra.1272

13. Pettit NE, Naiman RJ. Postfire response of flood-regenerating riparian vegetation in a semi-arid landscape. Ecology. 2007;88: 2094–2104. doi:10.1890/06-1270.1

14. Casady GM, Leeuwen WJD Van, Marsh SE. Evaluating post-wildfire vegetation regeneration as a response to multiple environmental determinants. Environ Model Assess. 2010;15: 295–307. doi:10.1007/s10666-009-9210-x

15. Dhungel S, Tarboton DG, Jin J, Hawkins CP. Potential effects of climate change on ecologically relevant streamflow regimes. River Res Appl. 2016;32: 1827–1840.

16. Bateman HL, Riddle SB. Reptile community responses to native and non-native riparian forests and disturbance along two rivers in Arizona. River Res Appl. 2020;36: 492–502. doi:10.1002/rra.3587

17. Hansen MC, Potapov P V., Moore R, Hancher M, Turubanova SA, Tyukavina A, et al. High-resolution global maps of 21st-century forest cover change. Science. 2013;342: 850–853. doi:10.1126/science.1244693

18. Jaeger KL, Olden JD, Pelland NA. Climate change poised to threaten hydrologic connectivity and endemic fishes in dryland streams. Proc Natl Acad Sci U S A. 2014;111: 13894–13899. doi:10.1073/pnas.1320890111

19. Leigh C, Datry T. Drying as a primary hydrological determinant of biodiversity in river systems: a broad-scale analysis. Ecography. 2017;40: 487–499. doi:10.1111/ecog.02230

20. Hof C, Araújo MB, Jetz W, Rahbek C. Additive threats from pathogens, climate and land-use change for global amphibian diversity. Nature. 2011;480: 516–519. doi:10.1038/nature10650

21. Cox N, Young BE, Bowles P, Fernandez M, Marin J, Rapacciuolo G, et al. A global reptile assessment highlights shared conservation needs of tetrapods. Nature. 2022;605: 285–290. doi:10.1038/s41586-022-04664-7

22. Prather CM, Pelini SL, Laws A, Rivest E, Bloch CP, Toro I Del, et al. Invertebrates, ecosystem services and climate change. Biol Rev. 2012;88: 327–348. doi:10.1111/brv.12002

23. Poloczanska ES, Brown CJ, Sydeman WJ, Kiessling W, Schoeman DS, Moore PJ, et al. Global imprint of climate change on marine life. Nat Clim Chang. 2013;3: 919–925. doi:10.1038/NCLIMATE1958

24. Pacifici M, Foden WB, Visconti P, Watson JEM, Butchart SHM, Kovacs KM, et al.

Assessing species vulnerability to climate change. Nat Clim Chang. 2015;5: 215–225. doi:10.1038/NCLIMATE2448

25. Ingram DJ, Ferreira GB, Jones KE, Mace GM. Targeting conservation actions at species threat response thresholds. Trends Ecol Evol. 2021;36: 216–226. doi:10.1016/j.tree.2020.11.004

26. Knouft JH, Ficklin DL. The potential impacts of climate change on biodiversity in flowing freshwater systems. Annu Rev Ecol Evol Syst. 2017;48: 111–133. doi:10.1146/annurev-ecolsys-110316-022803

27. Mitchell D, Maloney SK, Snelling EP, Hetem RS, Strauss WM, Fuller A. Revisiting concepts of thermal physiology: Predicting responses of mammals to climate change. J Anim Ecol. 2018;87: 956–973. doi:10.1111/1365-2656.12818

28. Bogan MT, Boersma KS, Lytle DA. Resistance and resilience of invertebrate communities to seasonal and supraseasonal drought in arid-land headwater streams. Freshw Biol. 2015;60: 2547–2558. doi:10.1111/fwb.12522

29. van de Pol M, Jenouvrier S, Cornelissen JHC, Visser ME. Behavioural, ecological and evolutionary responses to extreme climatic events: challenges and directions. Philos Trans R Soc B. 2017;372: 20160134. doi:10.1098/rstb.2016.0134

30. Franklin J. Predictive vegetation mapping: geographic modeling of biospatial patterns in relation to environmental gradients. Prog Phys Geogr. 1995;19: 474–499. doi:10.1177/030913339501900403

31. Guisan A, Zimmermann NE. Predictive habitat distribution models in ecology. Ecol

Modell. 2000;135: 147–186. doi:10.1016/S0304-3800(00)00354-9

32. Soberón J, Peterson AT. Interpretation of models of fundamental ecological niches and species' distributional areas. Biodivers Informatics. 2005;2: 1–10. doi:10.17161/bi.v2i0.4

33. Peterson AT, Soberón J. Species distribution modeling and ecological niche modeling: getting the concepts right. Nat Conserv. 2012;10: 102–107. doi:10.4322/natcon.2012.019

34. Kearney M, Porter W. Mechanistic niche modelling: combining physiological and spatial data to predict species' ranges. Ecol Lett. 2009;12: 334–350. doi:10.1111/j.1461-0248.2008.01277.x

35. Elith J, Leathwick JR. Species distribution models: Ecological explanation and prediction across space and time. Annu Rev Ecol Evol Syst. 2009;40: 677–697. doi:10.1146/annurev.ecolsys.110308.120159

36. Guillera-Arroita G, Lahoz-Monfort JJ, Elith J, Gordon A, Kujala H, Lentini PE, et al. Is my species distribution model fit for purpose? Matching data and models to applications. Glob Ecol Biogeogr. 2015;24: 276–292. doi:10.1111/geb.12268

37. Bryn A, Bekkby T, Rinde E, Gundersen H, Halvorsen R. Reliability in distribution modeling—a synthesis and step-by-step guidelines for improved practice. Front Ecol Evol. 2021;9: 658713. doi:10.3389/fevo.2021.658713

38. Yates KL, Bouchet PJ, Caley MJ, Mengersen K, Randin CF, Parnell S, et al. Outstanding challenges in the transferability of ecological models. Trends Ecol Evol. 2018;33: 790–802. doi:10.1016/j.tree.2018.08.001

39. Thuiller W, Lafourcade B, Engler R, Araújo MB. BIOMOD – a platform for ensemble

forecasting of species distributions. Ecography. 2009;32: 369–373. doi:10.1111/j.1600-0587.2008.05742.x

40. Hao T, Elith J, Arroita-Guillera G, Lahoz- Monfort JJ. A review of evidence about use and performance of species distribution modelling ensembles like BIOMOD. Divers Distrib. 2019;25: 839–852. doi:10.1111/ddi.12892

41. Sillero N, Arenas-Castro S, Enriquez-Urzelai U, Gomes Vale C, Sousa-Guedes D, Martínez-Freiría F, et al. Want to model a species niche? A step-by-step guideline on correlative ecological niche modelling. Ecol Modell. 2021;456: 109671. doi:10.1016/j.ecolmodel.2021.109671

42. Guillera-Arroita G. Modelling of species distributions range dynamics and communities under imperfect detection: advances, challenges, and opportunities. Ecography. 2017;40: 281–295.

43. Araújo MB, Anderson RP, Barbosa AM, Beale CM, Dormann CF, Early R, et al. Standards for distribution models in biodiversity assessments. Sci Adv. 2019;5: eaat4858. doi:10.1126/sciadv.aat4858

44. Schuetz JG, Langham GM, Soykan CU, Wilsey CB, Auer T, Sanchez CC. Making spatial prioritizations robust to climate change uncertainties: a case study with North American birds. Ecol Appl. 2015;25: 1819–1831.

45. Porfirio LL, Harris RMB, Lefroy EC, Hugh S, Gould SF, Lee G, et al. Improving the use of species distribution models in conservation planning and management under climate change. PLoS One. 2014;9: 1–21. doi:10.1371/journal.pone.0113749

46. Rossman DA, Ford NB, Seigel RA. The garter snakes: evolution and ecology. Norman: University of Oklahoma Press; 1996.

47. Willson JD, Winne CT. Evaluating the functional importance of secretive species: A case study of aquatic snake predators in isolated wetlands. J Zool. 2016;298: 266–273. doi:10.1111/jzo.12311

48. Hallas JM, Parchman TL, Feldman CR. Phylogenomic analyses resolve relationships among garter snakes (Thamnophis: Natricinae: Colubridae) and elucidate biogeographic history and morphological evolution. Mol Phylogenet Evol. 2022;167: 107374. doi:10.1016/j.ympev.2021.107374

49. González-Fernández A, Manjarrez J, García-Vázquez U, D'Addario M, Sunny A. Present and future ecological niche modeling of garter snake species from the Trans-Mexican Volcanic Belt. PeerJ. 2018;2018: e4618. doi:10.7717/peerj.4618

50. Griffis-Kyle KL, Mougey K, Vanlandeghem M, Swain S, Drake JC. Comparison of climate vulnerability among desert herpetofauna. Biol Conserv. 2018;225: 164–175. doi:10.1016/j.biocon.2018.06.009

51. Huey RB, Peterson CR, Arnold SJ, Porter WP. Hot rocks and not-so-hot rocks: Retreat-site selection by garter snakes and its thermal consequences. Ecology. 1989;70: 931–944. doi:10.2307/1941360

52. Holycross AT, Mitchell JC. Snakes of Arizona. 1st ed. ECO Publishing; 2020.

53. Blais BR, Johnson SL, Koprowski JL. Effects of disturbances and environmental changes on an aridland riparian generalist. PeerJ. 2023;11: e15563. doi:10.7717/peerj.15563

54. Wiens JJ. Climate-related local extinctions are already widespread among plant and animal species. PLoS Biol. 2016;14: e2001104. doi:10.1371/journal.pbio.2001104

55. Davis MA, Douglas MR, Webb CT, Collyer ML, Holycross AT, Painter CW, et al. Nowhere to go but up: Impacts of climate change on demographics of a short-range endemic (Crotalus willardi obscurus) in the sky-islands of southwestern North America. PLoS One. 2015;10: e0131067. doi:10.1371/journal.pone.0131067

56. Koprowski JL, Doumas SL, Merrick MJ, Oleson B, Posthumus EE, Jessen TG, et al. It’s lonely at the top: biodiversity at risk to loss from climate change. In: Gottfried GJ, Ffolliott PF, Gebow BS, Eskew LG, Collins LC, editors. Merging science and management in a rapidly changing world: Biodiversity and management of the Madrean Archipelago III. USDA Forest Service Proceedings RMRS-P-67; 2013. pp. 53–59.

57. Couet J, Marjakangas E-L, Santangeli A, Atle Kålås J, Lindström Å, Lehikoinen A. Short‐ lived species move uphill faster under climate change. Oecologia. 2022;198: 877–888. doi:10.1007/s00442-021-05094-4

58. USFWS. Endangered and Threatened Wildlife and Plants; Threatened Status for the Northern Mexican Gartersnake and Narrow-headed Gartersnake; Final Rule. Federal Register 79:38678–38746. 2014. Available: https://www.regulations.gov/document/FWS-R2-ES-2013-0071-0041

59. Wood DA, Emmons ID, Nowak EM, Christman BL, Holycross AT, Jennings RD, et al. Conservation genomics of the Mogollon Narrow-headed gartersnake (Thamnophis rufipunctatus) and Northern Mexican gartersnake (Thamnophis eques megalops). 2018. doi:10.3133/ofr20181141

60. Lowe CH. Arizona’s natural environment: landscapes and habitats. University of Arizona Press; 1964.

61. Forzieri G, Castelli F, Vivoni ER. Vegetation dynamics within the North American monsoon region. J Clim. 2011;24: 1763–1783. doi:10.1175/2010JCLI3847.1

62. Brown DE, Lowe CH. Biotic Communities of the Southwest. General Technical Report, Rocky Mountain Forest and Range Experiment Station, USDA Forest Service 1980 No.RM-78; 1980.

63. Georgescu M, Broadbent AM, Balling Jr RC. Effect of increased greenhouse gas concentration on mean, extreme, and timing of precipitation over Arizona (USA). Int J Climatol. 2022;42: 3776–3792. doi:10.1002/joc.7444

64. Pascale S, Boos WR, Bordoni S, Delworth TL, Kapnick SB, Murakami H, et al. Weakening of the North American monsoon with global warming. Nat Clim Chang. 2017;7: 806–812.

65. Munson SM, Bradford JB, Butterfield BJ, Gremer JR. Primary production responses to extreme changes in North American Monsoon precipitation vary by elevation and plant functional composition through time. J Ecol. 2022;110: 2232–2245. doi:10.1111/1365-2745.13947

66. Ernst CH, Ernst EM. Snakes of the United States and Canada. Washington D.C.: Smithsonian Institute Press; 2003.

67. GBIF.org. GBIF Occurrence Download. The Global Biodiversity Information Facility; 2023 [cited 3 May 2023]. doi:10.15468/DL.ANJP3W

68. Fick SE, Hijmans RJ. WorldClim 2: new 1- km spatial resolution climate surfaces for global land areas. Int J Climatol. 2017;37: 4302–4315. doi:10.1002/joc.5086

69. Salas EAL, Seamster VA, Harings NM, Boykin KG, Alvarez G, Dixon KW. Projected future bioclimate-envelope suitability for reptile and amphibian species of concern in south central USA. Herpetol Conserv Biol. 2017;12: 522–547.

70. O'Connor CD, Falk DA, Garfin GM. Projected climate-fire interactions drive forest to shrubland transition on an Arizona Sky Island. Front Environ Sci. 2020;8: 137. doi:10.3389/fenvs.2020.00137

71. Yanahan AD, Moore W. Impacts of 21st- century climate change on montane habitat in the Madrean Sky Island Archipelago. Biodivers Res. 2019;25: 1625–1638. doi:10.1111/ddi.12965

72. García-Franco JL, Gray LJ, Osprey S. The American monsoon system in HadGEM3 and UKESM1. Weather Clim Dyn. 2020;1: 349–371. doi:10.5194/wcd-1-349-2020

73. Inman RD, Esque TC, Nussear KE. Dispersal limitations increase vulnerability under climate change for reptiles and amphibians in the southwestern United States. J Wildl Manage. 2023;87: e22317. doi:10.1002/jwmg.22317

74. Wen X, Zhao G, Cheng X, Chang G, Donga X, Lin X. Prediction of the potential distribution pattern of the great gerbil (Rhombomys opimus) under climate change based on ensemble modelling. Pest Manag Sci. 2022;78: 3128–3134. doi:10.1002/ps.6939

75. Thuiller W, Guéguen M, Renaud J, Karger DN, Zimmermann NE. Uncertainty in ensembles of global biodiversity scenarios. Nat Commun. 2019;10: article 1446.

doi:10.1038/s41467-019-09519-w

76. Adler K, editor. Contributions to the History of Herpetology. Revised ed. Ithaca: Society for the Study of Amphibians and Reptiles; 2014.

77. Taggart TW, Collins JT, Schmidt CJ. Herpetological collections and collecting in Kansas. J Kansas Herpetol. 2006;17: 17–20.

78. Thuiller W, Georges D, Gueguen M, Engler R, Breiner F, Lafourcade B, et al. biomod2: Ensemble platform for species distribution modeling. 2023. Available: https://cran.r-project.org/package=biomod2

79. Aiello-Lammens ME, Boria RA, Radosavljevic A, Vilela B, Anderson RP. spThin: an R package for spatial thinning of species occurrence records for use in ecological niche models. Ecography. 2015;38: 541–545. doi:10.1111/ecog.01132

80. Sunny A, Manjarrez J, Caballero-Viñas C, Bolom-Huet R, Gómez-Ortiz Y, Domínguez-Vega H, et al. Modelling the effects of climate and land-cover changes on the potential distribution of three Earthsnakes (Genus Conopsis, Günther, 1858) in a highly anthropized area of Mexico. Sci Nat. 2023;110: 52. doi:10.1007/s00114-023-01880-7

81. Barbet-Massin M, Jiguet F, Albert CH, Thuiller W. Selecting pseudo-absences for species distribution models: How, where and how many? Methods Ecol Evol. 2012;3: 327–338. doi:10.1111/j.2041-210X.2011.00172.x

82. Li Z, Zhu Z, Wu Y. Scale dependency of pseudo-absences selection and uncertainty in climate scenarios matter when assessing potential distribution of a rare poppy plant Meconopsis punicea Maxim. under a warming climate. Glob Ecol Conserv. 2020;24:

e01353. doi:10.1016/j.gecco.2020.e01353

83. Chefaoui RM, Lobo JM. Assessing the effects of pseudo-absences on predictive distribution model performance. Ecol Modell. 2008;210: 478–486. doi:10.1016/j.ecolmodel.2007.08.010

84. Vollering J, Halvorsen R, Auestad I, Rydgren K. Bunching up the background betters bias in species distribution models. Ecography. 2019;42: 1717–1727. doi:10.1111/ecog.04503

85. Booth TH. Checking bioclimatic variables that combine temperature and precipitation data before their use in species distribution models. Austral Ecol. 2022;47: 1506–1514. doi:10.1111/aec.13234

86. Brun P, Thuiller W, Chauvier Y, Pellissier L, Wüest R, Wang Z, et al. Model complexity affects species distribution projections under climate change. J Biogeogr. 2020;47: 130–142. doi:10.1111/jbi.13734

87. Southwell D, Skroblin A, Moseby K, Southgate R, Indigo N, Backhouse B, et al. Designing a large- scale track- based monitoring program to detect changes in arid Australia. Ecol Appl. 2023;33: e2762. doi:10.1002/eap.2762

88. Austin MP, Van Niel KP. Improving species distribution models for climate change studies: Variable selection and scale. J Biogeogr. 2011;38: 1–8. doi:10.1111/j.1365-2699.2010.02416.x

89. Hansen EC, Scherer RD, Fleishman E, Dickson BG, Krolick D. Relations between environmental attributes and contemporary occupancy of threatened giant gartersnakes (Thamnophis gigas). J Herpetol. 2017;51: 274–283. doi:10.1670/16-143

90. Giermakowski JT, Hatten JR, Johnson MJ, Holmes JA, Nowak EM, Peters M. Predicting effects of climate change on riparian obligate species in the southwestern United States. Albuquerque, New Mexico: University of New Mexico; 2015. Available: http://hdl.handle.net/1928/31756

91. Hatten JR, Giermakowski JT, Holmes JA, Nowak EM, Johnson MJ, Ironside KE, et al. Identifying bird and reptile vulnerabilities to climate change in the southwestern United States. 2016. doi:10.3133/ofr20161085

92. Knapp RA. Effects of nonnative fish and habitat characteristics on lentic herpetofauna in Yosemite National Park, USA. Biol Conserv. 2005;121: 265–279. doi:10.1016/j.biocon.2004.05.003

93. Chandler HC, Jenkins CL, Bauder JM. Accounting for geographic variation in species-habitat associations during habitat suitability modeling. Ecol Appl. 2022;32: e2504. doi:10.1002/eap.2504

94. Wood SN. Fast stable restricted maximum likelihood and marginal likelihood estimation of semiparametric generalized linear models. J R Stat Soc B. 2011;73: 3–36. doi:10.1111/j.1467-9868.2010.00749.x

95. Khan S, Verma S. Ensemble modeling to predict the impact of future climate change on the global distribution of Olea europaea subsp. cuspidata. Front For Glob Chang. 2022;5: 997691. doi:10.3389/ffgc.2022.97769

96. R Core Team. R: A language and environment for statistical computing. R Foundation for Statistical Computing. Vienna, Austria: R Core Team; 2021. Available: https://www.r-project.org/

97. Baker Jr. MB. History of watershed research in the Central Arizona Highlands. Gen. Tech. Rep. RMRS-GTR-29. Fort Collins: U.S. Department of Agriculture, Forest Service, Rocky Mountain Research Station; 1999. doi:10.2737/RMRS-GTR-29

98. Bean WT, Stafford R, Brashares JS. The effects of small sample size and sample bias on threshold selection and accuracy assessment of species distribution models. Ecography. 2012;35: 250–258. doi:10.1111/j.1600-0587.2011.06545.x

99. Falk DA. Are Madrean Ecosystems Approaching Tipping Points? Anticipating Interactions of Landscape Disturbance and Climate Change. Biodivers Manag Madrean Archipel III. 2013; 40–47.

100. Sofaer HR, Jarnevich CS, Pearse IS, Smyth RL, Auer S, Cook GL, et al. Development and delivery of species distribution models to inform decision-making. Bioscience. 2019;69: 544–557. doi:10.1093/biosci/biz045

101. Noce S, Collalti A, Santini M. Likelihood of changes in forest species suitability, distribution, and diversity under future climate: the case of Southern Europe. Ecol Evol. 2017;7: 9358–9375. doi:10.1002/ece3.3427

102. Sequeira AMM, Bouchet PJ, Yates KL, Mengersen K, Caley MJ. Transferring biodiversity models for conservation: opportunities and challenges. Methods Ecol Evol. 2018;9: 1250–1264. doi:10.1111/2041-210X.12998

103. de Albuquerque FS, Bateman HL, Ryan MJ, Montgomery B. Model transferability and predicted response of a dryland anuran to climate change in the Southwest United States. J Biogeogr. 2024;51: 120–130. doi:10.1111/jbi.14733

104. Thomas KA, Stauffer BA, Jarchow CJ. Decoupling of species and plant communities of the U.S. Southwest: A CCSM4 climate scenario example. Ecosphere. 2023;14: e4414. doi:10.1002/ecs2.4414

105. Wogan GOU. Life history traits and niche instability impact accuracy and temporal transferability for historically calibrated distribution models of North American birds. PLoS One. 2016;11: e0151024. doi:10.1371/journal.pone.0151024

106. Paton RS, Matthiopoulos J. Defining the scale of habitat availability for models of habitat selection. Ecology. 2016;97: 1113–1122. doi:10.1890/14-2241.1

107. Holycross AT, Nowak EM, Christman BL, Jennings RD. Thamnophis rufipunctatus: Mogollon Narrow-headed Gartersnake. In: Holycross AT, Mitchell JC, editors. Snakes of Arizona. Rodeo, New Mexico: ECO Publishing; 2020. pp. 440–455.

108. Drost CA. Thamnophis elegans: Terrestrial Gartersnake. In: Holycross AT, Mitchell JC, editors. Snakes of Arizona. Rodeo, New Mexico: ECO Publishing; 2020. pp. 401–417.

109. Diele-Viegas LM, Figueroa RT, Vilela B, Rocha CFD. Are reptiles toast? A worldwide evaluation of Lepidosauria vulnerability to climate change. Clim Change. 2020;159: 581–599. doi:10.1007/s10584-020-02687-5

110. Naiman RJ, Décamps H, Pollock M. The role of riparian corridors in maintaining regional biodiversity. Ecol Appl. 1993;3: 209–212. doi:10.2307/1941822

111. Marshall RM, Robles MD, Majka DR, Haney JA. Sustainable water management in the southwestern United States: reality or rhetoric? PLoS One. 2010;5: e11687. doi:10.1371/journal.pone.0011687

112. Carlson MA, Lohse KA, McIntosh JC, McLain JET. Impacts of urbanization on groundwater quality and recharge in a semi-arid alluvial basin. J Hydrol. 2011;409: 196–211. doi:10.1016/j.jhydrol.2011.08.020

113. Auchincloss LC, Richards JH, Young CA, Tansey MK. Inundation depth, duration, and temperature influence Fremont cottonwood (Populus fremontii) seedling growth and survival. West North Am Nat. 2012;72: 323–333. doi:10.3398/064.072.0306

114. Ford NB. Thamnophis marcianus: Checkered Gartersnake. In: Holycross AT, Mitchell JC, editors. Snakes of Arizona. Rodeo, New Mexico: ECO Publishing; 2020. pp. 433–439.

115. Seigel RA, Ford NB, Mahrt LA. Ecology of an Aquatic Snake (Thamnophis marcianus) in a Desert Environment: Implications of Early Timing of Birth and Geographic Variation in Reproduction. Am Midl Nat. 2000;143: 453–462. doi:10.1674/0003-0031(2000)143[0453:EOAAST]2.0.CO;2

116. Myers EA, Xue AT, Gehara M, Cox CL, Davis Rabosky AR, Lemos-Espinal J, et al. Environmental heterogeneity and not vicariant biogeographic barriers generate community-wide population structure in desert-adapted snakes. Mol Ecol. 2019;28: 4535–4548. doi:10.1111/mec.15182

117. Sprague TA, Bateman HL. Influence of seasonality and gestation on habitat selection by northern Mexican gartersnakes (Thamnophis eques megalops). PLoS One. 2018;13: e0191829. doi:10.1371/journal. pone.0191829

118. Jones TR, Ryan MJ, Cotten TB, Servoss JM. Thamnophis eques: Mexican Gartersnake. In: Holycross AT, Mitchell JC, editors. Snakes of Arizona. Rodeo, New Mexico: ECO Publishing; 2020. pp. 418–432.

119. Blais BR, Wells SA, Poynter BM, Harris TR, Allard RA, Koprowski JL. Bridging conservation across the ex situ- in situ spectrum: Insights into the reproductive ecology of the threatened narrow- headed gartersnake (Thamnophis rufipunctatus). Zoo Biol. 2023;42: 429–439. doi:10.1002/zoo.21747

120. Jones KB. Habitat use and predatory behavior of Thamnophis cyrtopsis (Serpentes: Colubridae) in a seasonally variable aquatic environment. Southwest Nat. 1990;35: 115–122.

121. Jones TR, Hensley FR. Thamnophis cyrtopsis: Black-necked Gartersnake. In: Holycross AT, Mitchell JC, editors. Snakes of Arizona. Rodeo, New Mexico: ECO Publishing; 2020. pp. 388–400.

122. Vitasse Y, Ursenbacher S, Klein G, Bohnenstengel T, Chittaro Y, Delestrade A, et al. Phenological and elevational shifts of plants, animals and fungi under climate change in the European Alps. Biol Rev. 2021;96: 1816–1835. doi:10.1111/brv.12727

123. Brusca RC, Wiens JF, Meyer WM, Eble J, Franklin K, Overpeck JT, et al. Dramatic response to climate change in the Southwest: Robert Whittaker's 1963 Arizona Mountain plant transect revisited. Ecol Evol. 2013;3: 3307–3319. doi:10.1002/ece3.720

124. Crimmins TM, Crimmins MA, Bertelsen CD. Flowering range changes across an elevation gradient in response to warming summer temperatures. Glob Chang Biol. 2009;15: 1141–1152. doi:10.1111/j.1365-2486.2008.01831.x

125. Bellard C, Bertelsmeier C, Leadley P, Thuiller W, Courchamp F. Impacts of climate change on the future of biodiversity. Ecol Lett. 2012;15: 365–377. doi:10.1111/j.1461-0248.2011.01736.x

126. Bálint M, Domisch S, Engelhardt CHM, Haase P, Lehrian S, Sauer J, et al. Cryptic biodiversity loss linked to global climate change. Nat Clim Chang. 2011;1: 313–318. doi:10.1038/nclimate1191

127. Finn C, Grattarola F, Pincheira-Donoso D. More losers than winners: investigating Anthropocene defaunation through the diversity of population trends. Biol Rev. 2023; 1–17. doi:10.1111/brv.12974

128. Moritz C, Agudo R. The future of species under climate change: resilience or decline? Science. 2013;341: 504–508.

129. Dirzo R, Young HS, Galetti M, Ceballos G, Isaac NJB, Collen B. Defaunation in the Anthropocene. Science. 2014;345: 401–406. doi:10.1126/science.1251817

130. Bogan MT, Eppehimer D, Hamdhani H, Hollien K. If you build it, they will come: Rapid colonization by dragonflies in a new effluent-dependent river reach. PeerJ. 2020;8: e9856. doi:10.7717/peerj.9856

131. Lindenmayer DB, Wood JT, McBurney L, MacGregor C, Youngentob K, Banks SC. How to make a common species rare: A case against conservation complacency. Biol Conserv. 2011;144: 1663–1672. doi:10.1016/j.biocon.2011.02.022

132. Griffis-Kyle KL, Mougey K, Swain S, Drake J, Vanlandeghem M. Qualitative species vulnerability: model sensitivity to climate and spatial parameters. Clim Serv. 2021;22: 100217. doi:10.1016/j.cliser.2021.100217

133. Dubos N, Montfort F, Grinand C, Nourtier M, Deso G, Probst J-M, et al. Are narrow-ranging species doomed to extinction? Projected dramatic decline in future climate

suitability of two highly threatened species. Perspect Ecol Conserv. 2022;20: 18–28. doi:10.1016/j.pecon.2021.10.002

134. Hibbitts TJ, Painter CW, Holycross AT. Ecology of a population of the narrow-headed garter snake (Thamnophis rufipunctatus) in New Mexico: catastrophic decline of a river specialist. Southwest Nat. 2009;54: 461–467. doi:10.1894/GC-195.1

135. Keppel G, Mokany K, Wardell-Johnson GW, Phillips BL, Welbergen JA, Reside AE. The capacity of refugia for conservation planning under climate change. Front Ecol Environ. 2015;13: 106–112. doi:10.1890/140055

136. USFWS. Endangered and Threatened Wildlife and Plants; Designation of Critical Habitat for the Northern Mexican Gartersnake. Fed Regist. 2021;86: 22518–22580.

137. USFWS. Endangered and Threatened Wildlife and Plants; Designation of Critical Habitat for the Narrow-Headed Gartersnake. Fed Regist. 2021;86: 58474–58523.

138. Calabrese JM, Certain G, Kraan C, Dormann CF. Stacking species distribution models and adjusting bias by linking them to macroecological models. Glob Ecol Biogeogr. 2014;23: 99–112. doi:10.1111/geb.12102

139. Mi C, Ma L, Yang M, Li X, Meiri S, Roll U, et al. Global Protected Areas as refuges for amphibians and reptiles under climate change. Nat Commun. 2023;14: 1389. doi:10.1038/s41467-023-36987-y

140. Suggitt AJ, Wilson RJ, Isaac NJB, Beale CM, Auffret AG, August T, et al. Extinction risk from climate change is reduced by microclimatic buffering. Nat Clim Chang. 2018;8: 713–717. doi:10.1038/s41558-018-0231-9

141. Whiteley AR, Fitzpatrick SW, Funk WC, Tallmon DA. Genetic rescue to the rescue. Trends Ecol Evol. 2015;30: 42–49. doi:10.1016/j.tree.2014.10.009

142. Blais BR, Wells SA, Poynter BM, Koprowski JL, Garner MM, Allard RA. Adaptive management in a conservation breeding program: Mimicking habitat complexities facilitates reproductive success in narrow-headed gartersnakes (Thamnophis rufipunctatus). Zoo Biol. 2022;41: 346–353. doi:10.1002/zoo.21682

Supporting information

Dataset S1. Suitable environment range change output from ensemble species distribution modeling for *Thamnophis* gartersnakes in Arizona. (XLSX)

Table S1. Ensemble species distribution models for *Thamnophis* gartersnakes in Arizona, 1980–2021. Ensemble models are comprised of multiple single-algorithm models (GAM, GBM, and RF) with true skill statistics (TSS) scores >70%. Ensemble algorithms (*EM.algo*) include committee averaging (*Ca*) and weighted mean (*wmean*; [78]), evaluation metrics (*Eval.met*) include relative operating characteristic (*ROC*) and *TSS*, and reported values include ensemble model sensitivity (*sens*, i.e., true positive rate), specificity (*spec*, i.e., true negative rate), and calibration (*cal*).

Fig S1. Directional distribution trends of *Thamnophis* gartersnake occurrences in Arizona. Ellipses represent the central tendencies (mean center ±1 standard deviation in each XY coordinate) of the data. Three time periods are categorized to reflect historical change.

Fig S2. Estimated range changes across time for five *Thamnophis* gartersnake species in Arizona under three global climate models, two 20-year periods (median years 2050, 2090), and two shared socio-economic pathways (SSP). Data are derived from weighted-mean algorithms of ensemble species distribution models. Bars represent means ± standard error. Pathways include the most optimistic (SSP126) and pessimistic (SSP585) emissions-limiting models.

Fig S3a–y. Environmental suitability maps for *Thamnophis* gartersnakes in Arizona. Projections based on weighted-means ensemble models generated from the MRI-ESM2-0 global climate model. Data columns represent present (1980–2021), near future ("2050", i.e., 2041–2060 median), and distant future ("2090", i.e., 2081–2100 median) projections of two future shared socio-economic pathways (SSP) that represent optimistic (SSP126) and pessimistic (SSP585, i.e., status quo) emissions-limiting models.

Fig S4. Individual panels (a–t) from 'Fig 4 Projected change in range sizes for *Thamnophis* gartersnakes in Arizona.' Data are modeled from weighted-means ensemble models generated from the MRI-ESM2-0 global climate model. Changes based on present time modeling (1980–2021). Color values: yellow = suitable, stable; blue = suitable gained; red = lost, unsuitable; gray = unsuitable.

Figure 1

Click here to access/download;Figure;Fig_1.tif

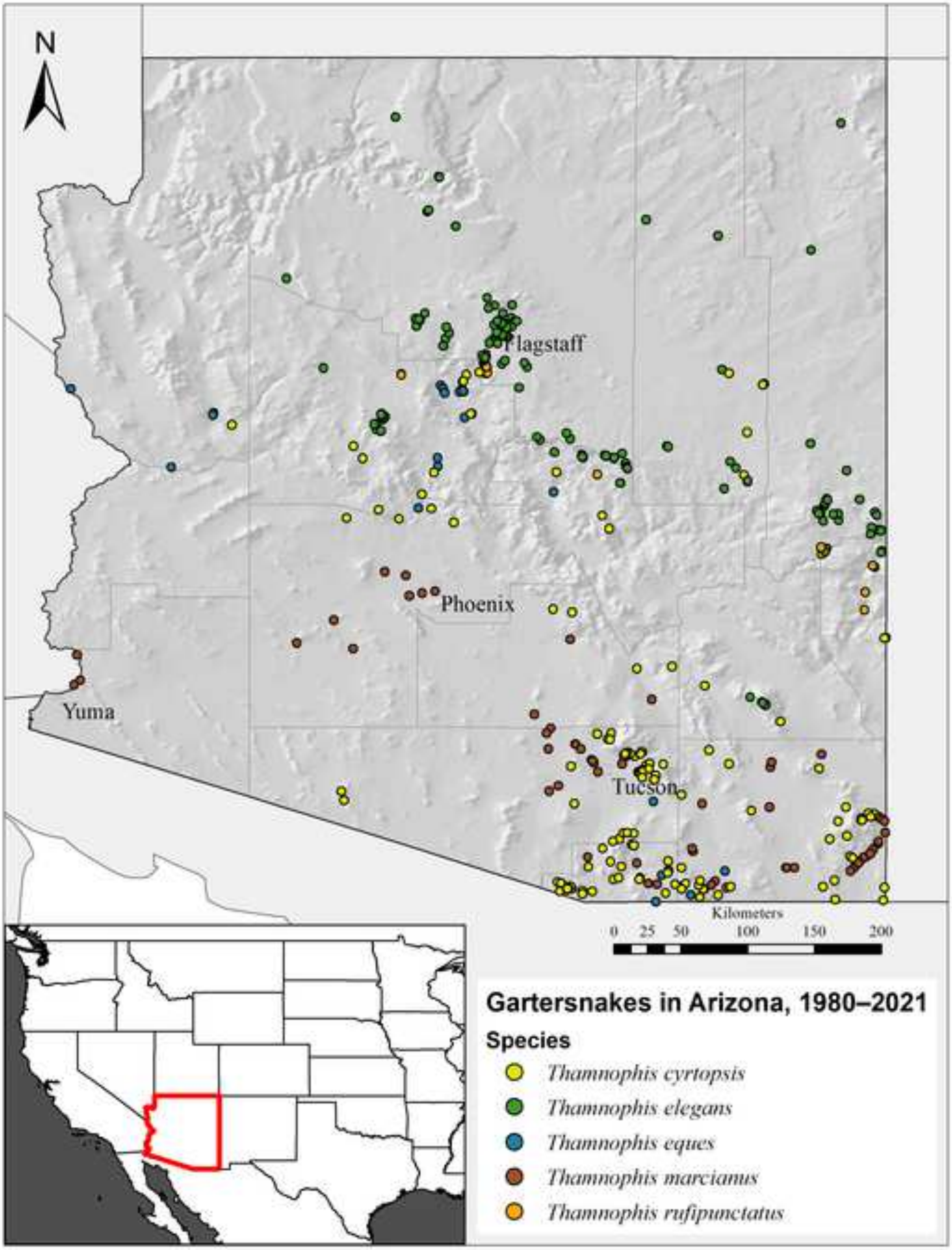

Figure 2

Click here to access/download;Figure;Fig_2.tiff

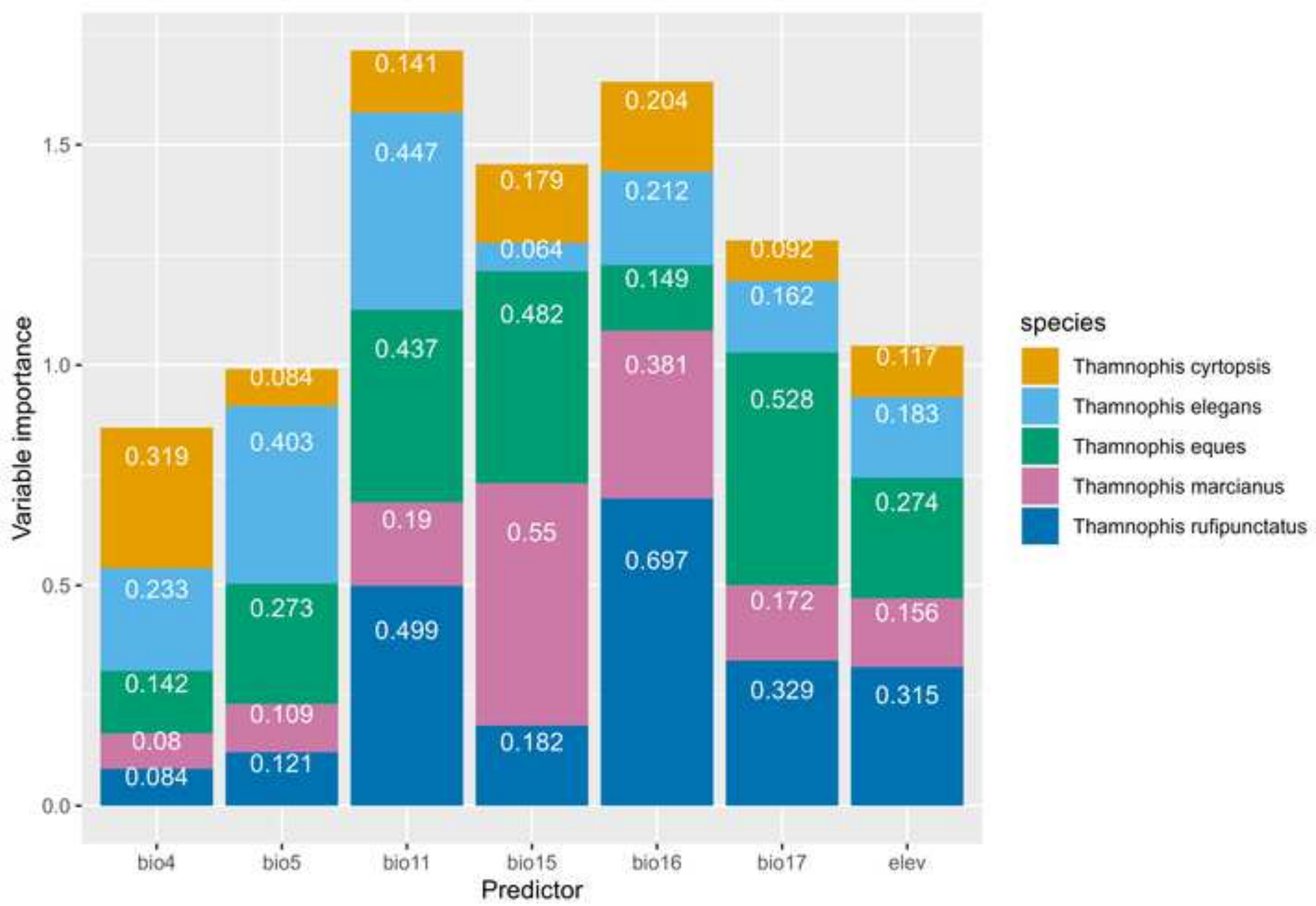

Figure 3

Click here to access/download;Figure;Fig_3.tiff

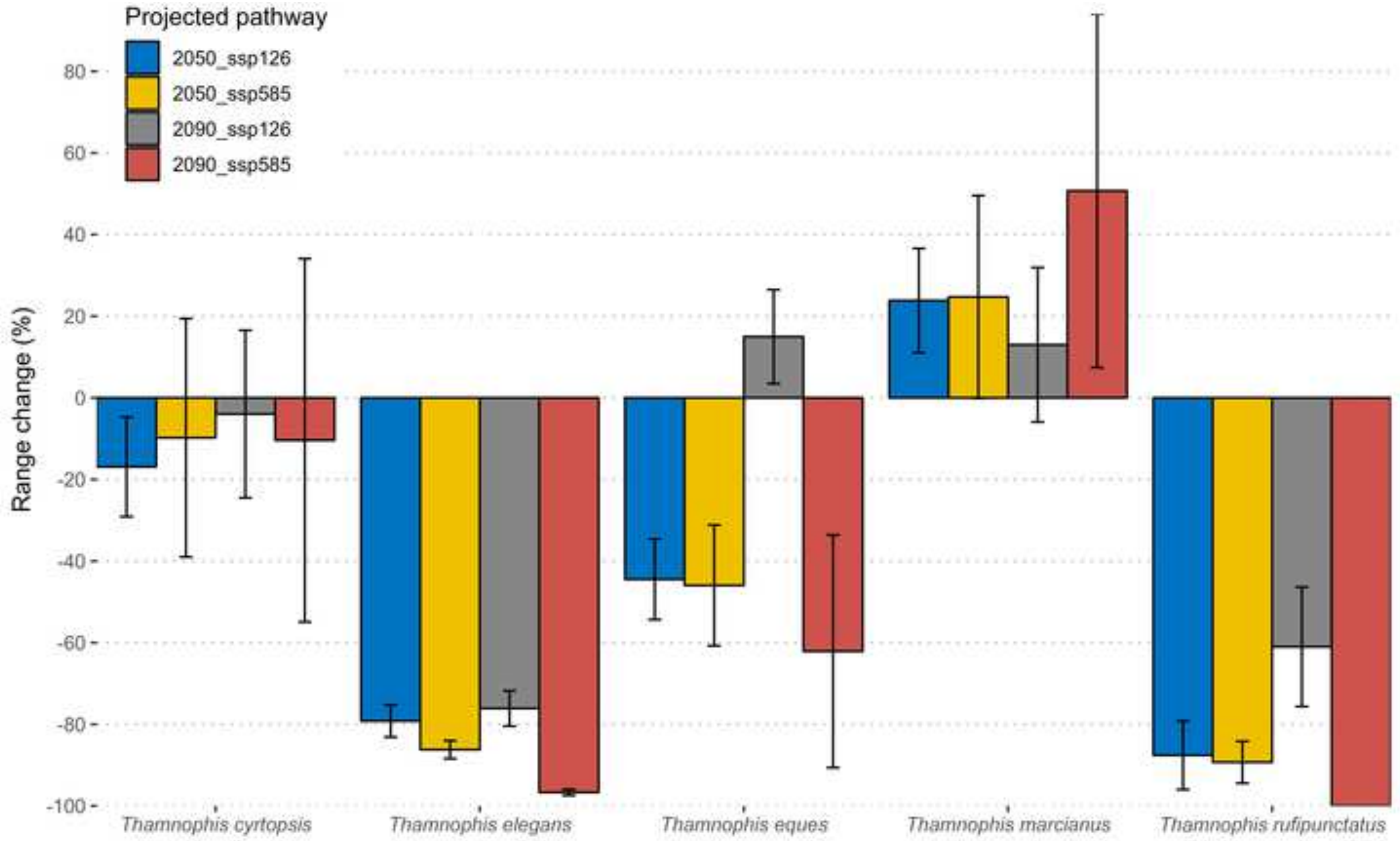

Figure 4

Click here to access/download;Figure;Fig_4.tif

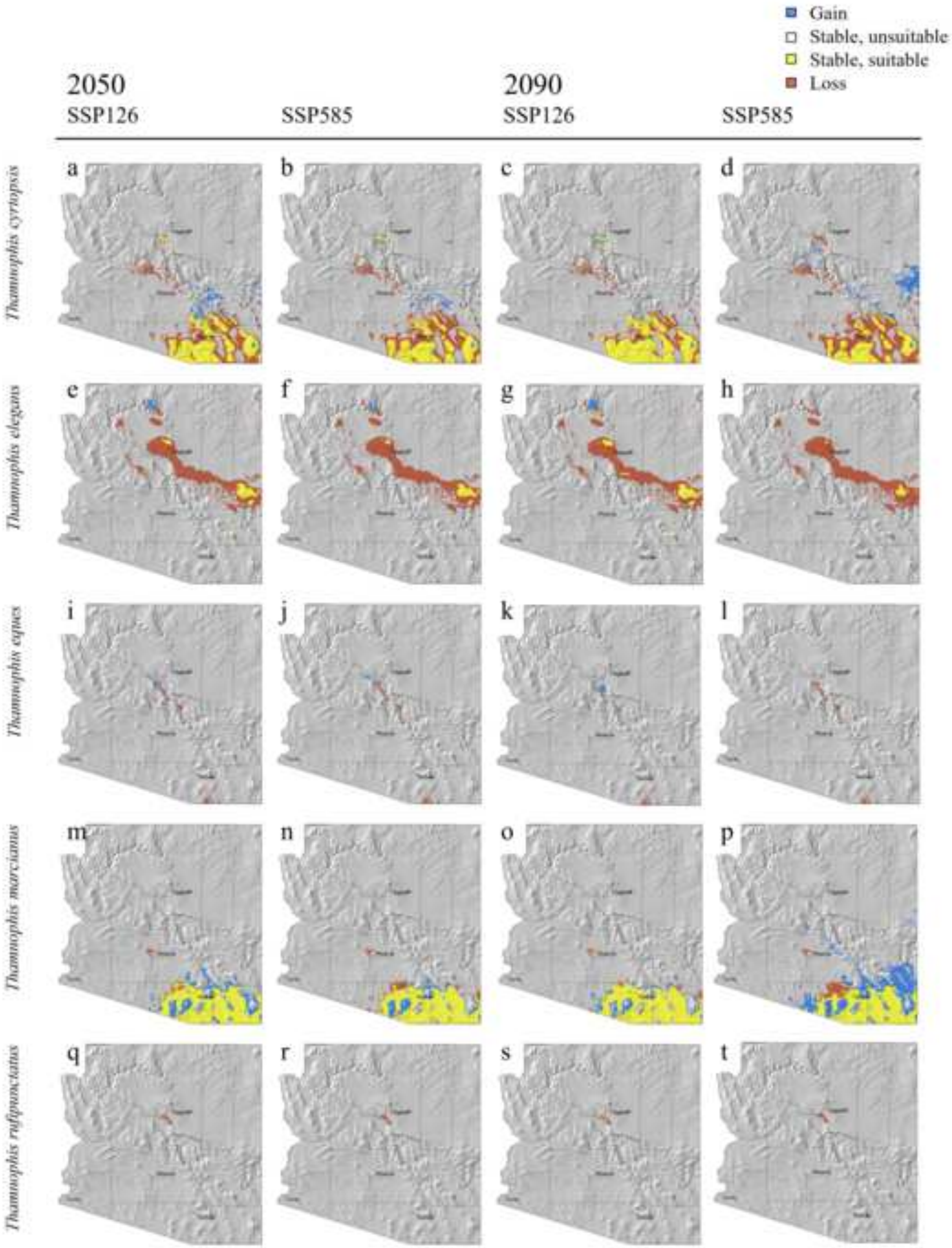

Table S1

Click here to access/download
Supporting Information
Table_S1.pdf

Fig S1

Click here to access/download
Supporting Information
Fig_S1.pdf

Fig S2

Click here to access/download
Supporting Information
Fig_S2.pdf

Fig S3

Click here to access/download
Supporting Information
Fig_S3.pdf

Fig S4

Click here to access/download
Supporting Information
Fig_S4.pdf

DatasetS1

Click here to access/download
Supporting Information
Dataset_S1.xlsx
