# Supplementary material for: Modeling a hot, dry future: Substantial range reductions in suitable environment projected under climate change for a semiarid riparian predator guild
Source: PLoS One. 2024 May 6;19(5):e0302981. doi: 10.1371/journal.pone.0302981 (PMC11073737; doi:10.1371/journal.pone.0302981)
Supplement: Attachment — Submitted filename: PONE-D-23-43716_Response-to-Reviewers.docx [file pone.0302981.s008.docx]

Dear Academic Editor Singh,

We are grateful to Academic Editor, Randeep Singh and the anonymous reviewers that provided additional feedback on our first manuscript revision. With such, our revision addresses their concerns and better translates the most important findings, implications, and focus for the future. Specifically, how findings herein can highlight conservation concerns for other taxa with similar life histories in changing systems.

Below, we address (in red text) each applicable comment, concern, and suggestion made by reviewers. Where appropriate, we add updated line numbers that correspond to the (clean) revision. We are grateful for the opportunity and believe the manuscript is now suitable for publication in *PLOS ONE*.

Reviewers' comments:

Reviewer's Responses to Questions

**Comments to the Author**

1. Is the manuscript technically sound, and do the data support the conclusions?

The manuscript must describe a technically sound piece of scientific research with data that supports the conclusions. Experiments must have been conducted rigorously, with appropriate controls, replication, and sample sizes. The conclusions must be drawn appropriately based on the data presented.

Reviewer #1: Partly

Reviewer #2: Yes

2. Has the statistical analysis been performed appropriately and rigorously?

Reviewer #1: Yes

Reviewer #2: Yes

3. Have the authors made all data underlying the findings in their manuscript fully available?

The [PLOS Data policy](http://www.plosone.org/static/policies.action#sharing) requires authors to make all data underlying the findings described in their manuscript fully available without restriction, with rare exception (please refer to the Data Availability Statement in the manuscript PDF file). The data should be provided as part of the manuscript or its supporting information, or deposited to a public repository. For example, in addition to summary statistics, the data points behind means, medians and variance measures should be available. If there are restrictions on publicly sharing data—e.g. participant privacy or use of data from a third party—those must be specified.

Reviewer #1: Yes

Reviewer #2: Yes

4. Is the manuscript presented in an intelligible fashion and written in standard English?

PLOS ONE does not copyedit accepted manuscripts, so the language in submitted articles must be clear, correct, and unambiguous. Any typographical or grammatical errors should be corrected at revision, so please note any specific errors here.

Reviewer #1: No

Our manuscript was written in standard English. We revised throughout for brevity, clarity, and to eliminate any ambiguous terminology. Without details, we are uncertain of any specific points in question.

Reviewer #2: Yes

5. Review Comments to the Author

Please use the space provided to explain your answers to the questions above. You may also include additional comments for the author, including concerns about dual publication, research ethics, or publication ethics. (Please upload your review as an attachment if it exceeds 20,000 characters)

Reviewer #1: Dear Editor
The manuscript about new records should be including comprehensive data on distribution patterns, ecology and taxonomy of target taxon. Moreover, biodiversity approach provide motivation for readers as well conservation managers. Accordingly, the manuscript should be improved to qualify a scientific manuscript including:

We include a literature review on the known distribution of our study subjects (*Thamnophis* in Arizona) in our ‘Study System’ section (lines 92-107). We also assessed and disseminate these data in graphic and tabular forms (Figs. 1, S1; Tables 1). The central premise of this study is determining environmental factors driving present-time distributions (i.e., species-environmental relationships) and forecast how future climate change might affect the spatiotemporal range of suitable environment of focal taxa. Broader ecology and taxonomy of the focal taxa digresses from this paper’s focus, but we added references to comprehensive works of those topics (e.g., Mitchell and Holycross 2020; lines 106-107).

We also revised our Discussion to offer focus more concisely on conserving biodiversity through important guilds such as *Thamnophis* in vulnerable aridland riparian systems. We respond to specific comments or topic points below.

General comments
Scientific names should be written based on taxonomical guidelines (Genus, species and authors)

We followed the PLOS ONE guidelines and referenced recent publications in the journal for application of scientific names, both in-text and Table 1.

The table, images and graphs can be complete the puzzle of study

We agree – the figures, tables, and supporting files directly support our central findings and help readers to visualize the data, such as the projected changes in suitable environment by area (Fig 4)

Introduction. The author(s) should be improve it including:
Comprehensive literature review

Our revised Introduction comprises a comprehensive literature review of our paper‘s scope (species distribution modeling and forecasting for conservation purposes)

The hypothesis, justification, necessity and aims of the study

Our purpose of study, objectives (3), applicable hypotheses, and broader implications are presented in the final paragraph of the Introduction section (lines 72-88). We synthesize hypotheses and findings throughout the Discussion.

Material and Methods should be classified to
Study area: Geography, geomorohology, geology, climate and pedology of area.

Please see the subsection “*Study area and system*” (lines 92-107) in Methods. There, we included a brief overview of relevant characteristics, such as climate and range of biotic communities among our study system. Other disciplines, such as geology and pedology, are outside the scope of this climatic SDM paper but we cite more comprehensive summaries of our study area (see Lowe 1964; Brown & Lowe 1980; Line 95)

Discussion
Discussion is weak. Accordingly must be improved significantly.
Comparison of results by last studies
Detailed analysis
The main achievements must be described by conservation and biodiversity approach
What are the main and key results of study?

We have revised our Discussion for clarity and brevity. We point out our main conclusions in the first paragraph of the Discussion (lines 322-325) and elaborate in appropriate subsections thereafter as they relate to our detailed analyses and results. Because much of this paper is about projecting changes to suitable environment in the future, there is a limit to comparing results to other studies. However, we do compare our work to other authors’ parallel SDM estimates of future changes, including those with taxa in common. We advance SDM science by assessing multiple combinations of models across various emissions pathways, which reduces uncertainty over single model algorithms as well as provides conservation practitioners with more bounded estimates of future environmental changes. We include comparisons to other SDM projection studies on lines 407-414; 419-422; 428-432, for example. Our “Future directions” section (lines 440-488) summarizes the broader biodiversity conservation implications made from our research. We revised to make our main findings clear.

Best Regards

Reviewer #2: The authors have predicted the changes in the distribution and range of suitable habitats for five species of gartersnakes by the species occurrence data during the past periods until present period and the environmental variables and using several predictive models. Data provided are valuable. However, it can be written in a better way and more concentrated, which I have specified in the attached file.

We have revised throughout, especially the Discussion, for clarity and brevity while retaining key findings of the study and the broad conservation implications therein. Responses to individual remarks are below.

6. PLOS authors have the option to publish the peer review history of their article ([what does this mean?](https://journals.plos.org/plosone/s/editorial-and-peer-review-process#loc-peer-review-history)). If published, this will include your full peer review and any attached files.


If you choose “no”, your identity will remain anonymous but your review may still be made public.


**Do you want your identity to be public for this peer review?** For information about this choice, including consent withdrawal, please see our [Privacy Policy](https://www.plos.org/privacy-policy).

Reviewer #1: No

Reviewer #2: No

***Line Comments by Reviewer #2***

Line 17-18: ‘strikethrough’

We have revised the statement for brevity and clarity (Lines 16-18)

Line 18: ‘strikethrough’

We have revised the statement for brevity and clarity (Lines 16-18)

Line 19-24: Please summarize

We agree that the statement could be simplified and have made such changes (lines 19-24)

Line 25: Do you mean during future scenarios? Specify

We revised for clarity (lines 24-25)

Line 25-27: Do you mean predictive variables in precent or future modeling? Please specify

This was helpful for clarity; we revised to state that the findings represented present relationships (lines 22-23), which can then be used to project (forecast) onto future climate scenarios.

Line 28: 37% {rephrasing}

Rephrased for brevity (Line 27)

Line 32: Please describe your results in more detail and summarize the importance of your findings at the end of the abstract at lines 32 to 38.

We focused on the most important findings of our study (lines 24-27) and summarized the big picture conclusion (Lines 27-37)

Line 123-125: ???Please rewrite

We cleaned up this entire paragraph for clarity (Lines 141-157)

Line 201-207: Please transfer it to the discussion section

We moved the statement in part to the Discussion (Lines 382-383) and deleted extraneous material.

Line 217: What do you mean? Do you mean data cleaning?

We rephased to “After spatially thinning contemporary occurrences to 1/km^2^, …” (line 208), which corresponds to the data thinning of ‘present period’ we presented in the Methods (lines 161-163)

Line 246: Please use the full names of variables instead of abbreviations

We revised throughout to include variable names for ease of reading

Line 246-247: ‘strikethrough’

We reject this suggestion to eliminate the callout to statistic reference and measure of uncertainty because we use various statistics and measures throughout and feel it may become unclear without specifying the test/measure; and PLOS ONE requires clear, detailed statistical reporting.

Line 249: ‘strikethrough’

We reject this suggestion to eliminate the callout to statistic reference and measure of uncertainty because we use various statistics and measures throughout and feel it may become unclear without specifying the test/measure; and PLOS ONE requires clear, detailed statistical reporting.

Line 250: ‘strikethrough’

We reject this suggestion to eliminate the callout to statistic reference and measure of uncertainty because we use various statistics and measures throughout and feel it may become unclear without specifying the test/measure; and PLOS ONE requires clear, detailed statistical reporting.

Line 252-253: ‘strikethrough’

We reject this suggestion to eliminate the callout to statistic reference and measure of uncertainty because we use various statistics and measures throughout and feel it may become unclear without specifying the test/measure; and PLOS ONE requires clear, detailed statistical reporting.

Line 255: ‘strikethrough’

We reject this suggestion to eliminate the callout to statistic reference and measure of uncertainty because we use various statistics and measures throughout and feel it may become unclear without specifying the test/measure and; PLOS ONE requires clear, detailed statistical reporting.

Line 275-280: ‘strikethrough’ {multiple}

We reject these suggestions to eliminate the callout to statistic reference and measure of uncertainty because we use various statistics and measures throughout and feel it may become unclear without specifying the test/measure; and PLOS ONE requires clear, detailed statistical reporting.

Line 326: There is no need to refer to numbers and results in the discussion

We removed result values here and inspected the Discussion for consistency

Line 338-339: ‘strikethrough’

We removed the text per recommendation

Line 349-352: ‘strikethrough’

We rephrased the statement and moved to a better location for relevance (lines 325-327)

Line 383-398: please summerize 383 to 398

We revised throughout the “*Projected changes among future scenarios*” (lines 371-449) section for brevity and clarity but retained many key points of synthesis due to species in our study having varying distributions, niches, and conservation status (see justification on lines 365-367, 385-387). We feel this information is critical for conservation managers of our focal taxa but are also applicable to other taxa that have similar life histories or occupy important aridland riparian systems and the conservation practitioners.

Line 439-443: ‘strikethrough’

We removed this redundant text
